# Multimodal measurement approach to identify individuals with mild cognitive impairment: study protocol for a cross-sectional trial

**DOI:** 10.1136/bmjopen-2020-046879

**Published:** 2021-05-25

**Authors:** Bernhard Grässler, Fabian Herold, Milos Dordevic, Tariq Ali Gujar, Sabine Darius, Irina Böckelmann, Notger G Müller, Anita Hökelmann

**Affiliations:** 1 Institute of Sport Science, Faculty of Humanities, Otto von Guericke University Magdeburg, Magdeburg, Germany; 2 Department of Neuroprotection, German Centre for Neurodegenerative Diseases Site Magdeburg, Magdeburg, Germany; 3 Occupational Medicine, Otto von Guericke University Medical Faculty, Magdeburg, Germany; 4 Department of Neurology, Otto von Guericke University Medical Faculty, Magdeburg, Germany

**Keywords:** cardiology, mental health, physiology, dementia, neurophysiology

## Abstract

**Introduction:**

The diagnosis of mild cognitive impairment (MCI), that is, the transitory phase between normal age-related cognitive decline and dementia, remains a challenging task. It was observed that a multimodal approach (simultaneous analysis of several complementary modalities) can improve the classification accuracy. We will combine three noninvasive measurement modalities: functional near-infrared spectroscopy (fNIRS), electroencephalography and heart rate variability via ECG. Our aim is to explore neurophysiological correlates of cognitive performance and whether our multimodal approach can aid in early identification of individuals with MCI.

**Methods and analysis:**

This study will be a cross-sectional with patients with MCI and healthy controls (HC). The neurophysiological signals will be measured during rest and while performing cognitive tasks: (1) Stroop, (2) N-back and (3) verbal fluency test (VFT). Main aims of statistical analysis are to (1) determine the differences in neurophysiological responses of HC and MCI, (2) investigate relationships between measures of cognitive performance and neurophysiological responses and (3) investigate whether the classification accuracy can be improved by using our multimodal approach. To meet these targets, statistical analysis will include machine learning approaches.

This is, to the best of our knowledge, the first study that applies simultaneously these three modalities in MCI and HC. We hypothesise that the multimodal approach improves the classification accuracy between HC and MCI as compared with a unimodal approach. If our hypothesis is verified, this study paves the way for additional research on multimodal approaches for dementia research and fosters the exploration of new biomarkers for an early detection of nonphysiological age-related cognitive decline.

**Ethics and dissemination:**

Ethics approval was obtained from the local Ethics Committee (reference: 83/19). Data will be shared with the scientific community no more than 1 year following completion of study and data assembly.

**Trial registration number:**

ClinicalTrials.gov, NCT04427436, registered on 10 June 2020, https://clinicaltrials.gov/ct2/show/study/NCT04427436.

**Strengths and limitations of this study**

- This study will be the first to use a multimodal measuring approach to determine neurophysiological responses of elderly with mild cognitive impairment and healthy controls.
- Differences between these two groups will be investigated with electroencephalography, functional near-infrared spectroscopy and heart rate variability at resting state and while performing cognitive tasks.
- This study will provide valuable information about certain neurophysiological parameters that are promising for an early identification of people who are at a higher risk of an overly age-related decline in cognitive performance.
- We hypothesise that this multimodal approach improves the classification accuracy between elderly with mild cognitive impairment and healthy controls as compared with a unimodal or a bimodal approach.
- Since this study is the first of its kind, it has an exploratory character with a relatively small sample size, but it should provide a basic concept for large-scale studies.

## Introduction

Mild cognitive impairment (MCI) is considered as a prodromal stage of (or transition phase to) dementia. The most common cause of dementia is Alzheimer’s disease (AD).^1^ It is estimated that approximately 50 million people are currently suffering from AD worldwide.^2^ AD is a neurological disease with specific neuropathological (eg, amyloid-plaques, neurofibrillary tangles) and neurochemical features (eg, neurotransmitter deficits). On the behavioural level, AD is characterised by deficits of higher cortical functions such as memory, decision-making, visuospatial abilities, executive functioning and language.^3^ Individuals who suffer from dementia require a high level of care and support, and, thus, the disease entails a substantial burden for the healthcare systems.^4^ For instance, in Germany, the annual costs are estimated at 42.6 billion Euros,^5^ whereas the global costs are considered to have crossed the US$1 trillion threshold in 2018.^6^ Given that the number of individuals with dementia will increase further as a consequence of the demographic change, these annual healthcare expenditures are expected to grow, too.

In contrast to patients with dementia, patients with MCI have only marginal limitations in activities of daily living but show some deteriorations in specific cognitive domains.^7^ A person is considered to suffer from MCI if the following criteria are fulfilled: memory complaint, normal activities of daily functioning, normal general cognitive function, abnormal memory for age and not demented.^8^ There are four types of MCI: amnestic MCI single domain, amnestic MCI multiple domain, nonamnestic MCI single domain and nonamnestic MCI multiple domain.^9^ Patients with amnestic MCI show impairments in the performance of neuropsychological tests of episodic memory. Patients with nonamnestic MCI show impairments in cognitive domains other than memory, such as executive functions, language or visuospatial abilities.^8^ For a correct detection of MCI, a careful and comprehensive neuropsychological test battery covering multiple cognitive domains is an important criterion.^8^ Therefore, a correct detection of MCI by clinical data, regardless of whether single-domain and multiple-domain MCI are present, is relevant for our investigation. Hence, individuals with amnestic and nonamnestic MCI, based on a comprehensive clinical and neuropsychological assessment, without differentiating between single and multiple domain MCI, will be considered in our investigation. As these MCI are often not evident on a behavioural level, individuals with MCI are difficult to identify. During the stage of MCI, reduction of lifestyle-related risk factors, such as physical inactivity, may slow down or postpone the progression of this neurological syndrome.^10–12^ So far, no drug has been approved for the symptomatic or causal treatment of MCI.^13^ Nevertheless, an early diagnosis of MCI is indispensable for a better understanding of the neurobiological mechanisms mediating the transition of the inexorable age-related cognitive decline to dementia. This, in turn, may allow for a timely initiation of preventive strategies slowing, postponing or, at best, counteracting the transition to a serious manifestation of dementia.

The diagnosis of MCI based on clinical features alone is often challenging and relatively unreliable. Therefore, the identification of biomarkers that aid an early identification of neurodegenerative processes has become a focus of current research.^14^ Neuroimaging methods such as MRI or positron emission tomography (PET) are popular tools in the investigation of neurodegenerative diseases. However, these methods have several disadvantages. They are expensive, have limited eligibility and the participant is not allowed to move. Additionally, the use of radioisotopes in PET results in exposure to ionising radiation, which involves detrimental health risks.^15 16^ Biomarkers in the cerebrospinal fluid (CSF), such as Aβ42, constitute another approach for identifying AD pathology.^1^ However, drawing CSF is stressful for the patient, can entail complications and does not necessarily yield unequivocal results. Thus, it is necessary to investigate other measurement methods able to detect a pathological cognitive decline timely while avoiding the above-mentioned limitations.

fNIRS is an optical neuroimaging technique that is based on the theory of neurovascular coupling and optical spectroscopy.^17^ fNIRS allows the noninvasive investigation of cortical haemodynamic changes associated with brain activity.^18^ In this regard, it is assumed that neuronal activity enhances the regional cerebral blood flow to the neuronal tissue at work which, in turn, leads to a local increase in the concentration of oxygenated haemoglobin (oxyHb) and decrease in deoxygenated haemoglobin (deoxyHb).^17^ Since oxyHb and deoxyHb have different light absorption spectra, the relative activity-dependent concentration changes of oxyHb and deoxyHb can be determined and allow us to make conclusions about cortical brain activity.^16^ More details on physiological and methodological background of fNIRS can be found in the referenced literature.^17 19 20^

The advantages of fNIRS are its portability, tolerance to motion artefacts (compared with EEG and MRI), higher temporal resolution (compared with MRI), low costs (compared with MRI and PET), it is easy to use and more participants are eligible than for MRI (eg, no restriction due to claustrophobia or metallic implants). The disadvantages are its lower spatial resolution (compared with MRI), low penetration depth, lack of standardisation in data analysis and, due to the delay in the haemodynamic response, there is a time delay in the signal curve, too.^16^

fNIRS has become a frequently used technique for the investigation of neurological diseases such as AD.^21–23^ There is evidence in the literature showing that fNIRS offers a great potential to become a valuable tool to identify individuals with a higher risk of developing dementia as changes in cortical haemodynamic obtained during a standardised cognitive test allow to discern between healthy and diseased individuals.^24–31^ Young participants showed a stronger activation in the left hemisphere in the more difficult task conditions. Elderly participants showed no lateralisation and a decreased activation in the difficult task condition. This phenomenon is called ‘Hemispheric Asymmetric Reduction in OLD adults’ model.^32^

Electroencephalogram (EEG) is another technique for quantifying neurophysiological processes. EEG measures electrical activity provoked by the firing of cortical neurons within the brain. EEG is an optimal tool for recording the complex dynamic neural activity due to the excellent temporal resolution in milliseconds and reasonable spatial resolution.^33^ The potential to detect predementia AD/MCI condition has already been shown.^34–37^

In addition to the most commonly used method of frequency analysis, it is also recommended to apply other more sophisticated EEG methods, like brain mapping, connectivity analysis or the analysis of event-related potentials (ERPs), which can lead to a further specification of the diagnosis of dementia.^38–42^

The specific neural activity in response to a certain stimulus can be measured by ERPs. The most common component we can find in response to sensory, cognitive or motor stimuli are P100, P200, P300, P600, N100 and N400, which are used in cognitive science.^43^ Importantly, it is not sufficient to study the cortical modulation in isolation. Cortical functions are influenced by functional integration. EEG is an optimal technique to understand the communication between different areas of the brain by functional and effective connectivity analysis. Functional connectivity is defined as the correlation between different areas of the brain. Effective connectivity is defined as direct or indirect influence that one neural system exerts on the other neural system. The connectivity can be investigated by sources and EEG channels.^44–46^ Beside connectivity, it is also possible to construct the brain map from the recording of electrical potentials with electrode distribution over the skull. The brain mapping can be helpful to identify activated structures of the cortex during task execution.^47 48^

ECG will be the third assessment method. In particular, we will determine the heart rate variability (HRV) from the ECG. The HRV describes the beat-to-beat variation of subsequent NN intervals. These fluctuations in the heart rate are regarded as an indicator of the functional state of the autonomic nervous system (ANS) and is related to psychophysiological aspects such as self-regulation on a cognitive, emotional, social and health level.^49 50^ HRV is the result of the complex interaction between sympathetic and parasympathetic influences.^51 52^ A relatively low HRV indicates an increased sympathetic state, an abnormal regulation and inadequate adaptation of the cardiovascular system and is a sign of depletion or pathology. Hence, HRV is a promising marker to diagnose pathological states^52^ and an optimal HRV is critical to health and well-being.^53^

HRV measurements provide the advantages that they are easy to use, not expensive, portable, noninvasive, and the recordings are relatively easy to interpret. However, on the downside, HRV values are strongly influenced by individual differences, there is a relatively large number of influencing factors that have to be controlled for, and there is still no generally accepted consensus concerning data acquisition and data processing.^49 54 55^

Measures of HRV have been used to index vagal activity.^56^ Root mean square of successive differences (RMSSD) between adjacent normal NN intervals, percentage of successive normal NN intervals differing more than 50 ms (pNN50) and power in high (0.15–0.40 Hz) frequencies (HF) are supposed to reflect vagally mediated HRV.^49 53 57^ According to the neurovisceral integration model, neural structures responsible for affective, cognitive and physiological regulation are associated with vagally mediated cardiac function.^58^ Especially the prefrontal cortex is associated with HRV measures as it is connected with the amygdala and cardiovascular system.^59^ Vagally mediated HRV is supposed to be linked with ‘a set of neural structures that have been implicated in cognitive, especially executive function’.^58^ A positive connection between resting HRV and cognitive functioning has already been demonstrated.^60–64^ In a recent meta-analysis, the effect size (ES) magnitude of HRV measures in the evaluation of autonomic dysfunction in older people with dementia was investigated.^65^ Although the small ES does not support the use of HRV as a single biomarker to diagnose dementia, the results suggest autonomic dysfunction in dementia. Since biological processes such as ANS activity are complex and nonlinear, several authors suggest nonlinear HRV measures.^66–70^ In this context, sample entropy recorded at rest, was related to a better cognitive performance, but traditional time or frequency indices were not.^69^ The sensitivity and reliability of nonlinear measures such as Poincaré and detrended fluctuation analysis for mental effort tasks could be proved in healthy seniors.^67^ In a recent study, D2, the RRI dimension correlation, could be better related to the mental workload than time or frequency indices.^66^

Cognition relies on complex neurophysiological processes. In particular, the solving of a cognitive task is associated with task-related changes in cerebral haemodynamics, cerebral electrical activity and changes in the ANS. These task-related neurophysiological changes can be assessed by fNIRS, EEG and HRV^71^ separately (unimodal approach) or simultaneously (multimodal approach). A clear advantage of the multimodal assessment approach of task-related neurophysiological changes (eg, cognition-related brain activity) is its ability to reduce and/or to compensate for inherent limitations of a single measurement modality (eg, artefacts that are reflected in only one modality).^72^ As single tests, they might be not accurate enough for differentiating healthy controls (HC) from cognitively impaired participants. EEG, fNIRS and HRV provide complementary information about different neurophysiological systems which, in turn, foster an improvement of the classification accuracy. Indeed, cognitive neuroscience research now focuses on the simultaneous acquisition by noninvasive modalities to improve their performance and information content.^15^ Combined fNIRS-EEG,^73^ EEG-HRV^74 75^ and fNIRS-HRV^76^ measurements were already applied in different fields of brain research. It was observed that classification accuracy can be improved when using different measurement modalities simultaneously.^77–84^ However, so far and to the best of our knowledge, there is no published study available, which uses the above-described multimodal approach to investigate the neurophysiological cognition-related differences between healthy older individuals and older individuals with MCI.

The primary aim of this study is to investigate the difference between HC and cognitively impaired participants in neurophysiological signals. We will use fNIRS, EEG and ECG since they are noninvasive and provide some advantages compared with MRI, PET and measures of CSF. As the most of the currently available studies used, to the best of our knowledge, only two different measurement modalities (mostly EEG and fNIRS), we hypothesise that our approach improves classification accuracy considerably. The second aim of this study is to investigate possible neurobehavioral relationships between measures of cognitive performance and measures of fNIRS, EEG and HRV. The third aim of this study is to systematically compare the classification accuracy of uni-, bi- and multimodal measurement approaches. By saying that, we wish to emphasise that this study has an explorative character aiming to identify cognition-related neurophysiological parameters that are promising for MCI detection. This study should provide a basic concept for further studies using a multimodal measurement approach and promote research for a better understanding of the neurobiological mechanisms leading to dementia.

## Methods and analysis

### Study design

This cross-sectional study will be conducted by a multidisciplinary team of researchers from Department of Sport Science at the Otto von Guericke University Magdeburg, the Medical Faculty of the Otto von Guericke University Magdeburg and the German Center for Neurodegenerative Diseases. The study protocol was approved by the Ethics Committee of the Otto von Guericke University Magdeburg (reference: 83/19) and is in accordance with the Declaration of Helsinki. This study was registered in ClinicalTrials.gov on the 10 June 2020.

### Participants

This study has an explorative character as there is, to the best of our knowledge, no comparable study available, which used measures from multiple modalities (eg, EEG, fNIRS, HRV) to differentiate between individuals suffering from MCI and HC. Thus, we considered studies comparing individuals with MCI and HC using, at least, one measurement modality^29 85 86^ for our sample size estimation. In this context, the following calculations have been performed.

Based on the means of oxyHb of individuals with MCI and HC during a cognitive task in the study of Yang *et al*,^29^ a sample size of 15 participants in each group will be needed to achieve a statistical power of 80%. Based on the HF nu values during standing position in one HRV study,^85^ a sample size of 49 participants in each group will be needed to achieve a statistical power of 80%. Finally, based on the latency of P300 during an event-related task in one EEG study,^85^ a sample size of 34 participants in each group will be needed to achieve a statistical power of 80%. All sample sizes were calculated a priori by using G*Power V.3.1.^87^ Furthermore, studies that had applied EEG, fNIRS and ECG simultaneously in a cohort of healthy subjects^71 82 88^ used sample sizes varying between 11 and 25 subjects. Given the explorative character of this study and the intention to pave the way for future investigations with a larger sample size, we chose a relatively small sample with 30 MCI subjects and 30 HC constituting a tempered and conservative estimate to detect possible neurophysiological effects and/or trends.

This study will involve patients with MCI who have been diagnosed by an experienced neurologist, based on standardised clinical and neuropsychological criteria.^89^ Healthy participants will be recruited by advertisements in local newspapers. Interested individuals will be informed about the aim of the study and first be screened by telephone to check for general eligibility according to our criteria. The two groups will be age, gender, handedness and education matched. At the beginning of the study, each participant will provide a personally signed and dated informed consent document indicating that the individual has been informed of all pertinent aspects of the study. Participants must be native German-speaking adults who are between 55 and 80 years old. All participants will be financially rewarded to compensate for their participation.

Exclusion criteria are:

- Other neurological diseases (ie, epilepsy, multiple sclerosis).
- Known severe cardiac diseases (ie, history of heart disease, severe cardiac insufficiency, heart failure, cardiac pacemaker, valvular defect, with or without stent implantation, heart attack).
- Stroke.
- Mental diseases (ie, schizophrenia, depression).
- Orthopaedic diseases (ie, bone fracture in last 6 months, symptomatic slipped disc).
- Muscular diseases (ie, myositis, tendovaginitis).
- Severe endocrinologic diseases (ie, manifest hypothyroidism or hyperthyroidism, adiposity (BMI >30), juvenile-onset diabetes).
- Injury or surgery in last 6 months.
- Use of illegal intoxicants or alcohol abuse (more than three times per week).
- Uncorrected poor eyesight or hearing.
- Colour blindness/red-green weakness.
- Pregnancy or breastfeeding.
- Using one of the following medications: betablocker, angiotensin-converting-enzyme inhibitor, antiarrhythmic drugs, neuroleptics, narcotic analgesics, benzodiazepines and psychoactive medications.

At the beginning of the study, all participants will be screened by using the CERAD (Consortium to establish a registry for Alzheimers’s Disease) test battery.^90^ This cognitive test battery evaluates the performance in semantic verbal fluency, word retrieval, constructional praxis, visual memory, verbal memory, global cognition (mini mental state examination) and motor speed (trail making test A). The CERAD has been shown to be a valid and reliable assessment tool to identify individuals with cognitive impairments.^91^

### Assessments

Participants will attend three visits within 1 week and undergo the following assessments:

1. Clinical assessment: CERAD and medical examination, taking blood sample (ie, Apolipoprotein 4 and brain-derived neurotrophic factor).
2. Sociodemographic assessment: questionnaires.
3. Neurophysiological and neuropsychological assessment: EEG/fNIRS/ECG simultaneously at resting state and during cognitive tasks.

### Sociodemographic assessment

Sociodemographic data, family history of AD/dementia and lifestyle factors of the participants will be obtained before the measurements via several questionnaires. We will record medication, together with measurements of height, weight, educational and physical activity level. The level of physical activity will be assessed via the questionnaire German-PAQ50+.^92^ It will allow for this factor to be considered as a covariate. The health-related quality of life will be measured by the 36-Item Short Form Health Survey.^93^ Sleep quality will be assessed via the Pittsburgh Sleep Quality Index.^94^ Finally, the Food Frequency Questionnaire will be applied to assess the dietary habit of the participants.^95^

### Neurophysiological and neuropsychological assessment

Research staff collecting data are blinded concerning the cognitive status (MCI or HC) of the participant to avoid bias. EEG, fNIRS and ECG will be recorded simultaneously at resting state for 10 min. The measurement standards for the resting state measurement follow the recommendations of Laborde *et al*.^49^ Accordingly, the participants are asked to sit on a comfortable chair with their knees bent at a 90° angle and their hands on their thighs. Furthermore, they are advised to relax, breath normally and move as little as possible. As recommended in the literature, the participants will rest in the above-described position for at least 5 min before the baseline recordings are obtained.^49^ Subsequently, the participants complete three cognitive tasks while their neurophysiological signals are measured. The cognitive tasks are Stroop, N-back and a VFT. This procedure is comparable to the study of Yang *et al*^29^ but in addition to this study, (1) we will conduct the assessment of EEG and ECG and (2) we will use modified versions of the cognitive tasks. Afterwards, a second resting-state measurement will be conducted (recovery phase).^49^

According to the multistage concept in psychophysiology of Fahrenberg, objective data of the cognitive performance, objective physiological data and subjective data of the personal feeling will be collected.^96^ The latter will be recorded by the NASA-TLX questionnaire (National Aeronautics and Space Administration - Task Load Index). The NASA-TLX provides an overall workload score with six dimensions: mental demands, physical demands, temporal demands, own performance, effort and frustration.^97^ The overall workload is the weighted average of these dimensions. After the cognitive tests, participants will be instructed to rate each dimension on a visual scale from 1 to 20 points. It is an easy to use tool and its results can be compared with participants’ performance and their neurophysiological responses. The NASA-TLX is a widely used tool with a high reliability and validity.^98^

#### Cognitive tasks

During the whole assessment, the participants are sitting in front of a computer screen on a comfortable chair and are asked to avoid head movements as much as possible. Before the experiment, the participants will be briefed on the task instructions and experimental design. The instructions are presented on a printed paper and explained verbally by the investigator. Each task block begins with a 5 s instruction cue that informs the participant about the task condition. All tasks will be administered via computer, which allows to measure reaction times and responses of the participants exactly. To synchronise all signals, temporal triggers are delivered simultaneously to EEG and fNIRS systems via the software Presentation (Neurobehavioral Systems, USA).

##### Stroop

A computerised version of the Stroop test will be applied.^99^ This widely used test requires the executive functions inhibition and cognitive control. These are crucial for completion of complex cognitive tasks and everyday activities.^100^ The classic Stroop test elicits a conflict situation since the meaning of a colour-word and the ink colour do not match. Behavioural responses to these incongruent stimuli are usually slower and less accurate than responses to congruent stimuli. The behavioural difference between incongruent and congruent stimuli is called the Stroop effect, which has been used as an index of cognitive control.^101^ Since the prefrontal cortex plays a predominant role in cognitive control^102^ and has shown to be affected by MCI,^23^ fNIRS optodes will be placed on the prefrontal cortex.

**Figure 1:**
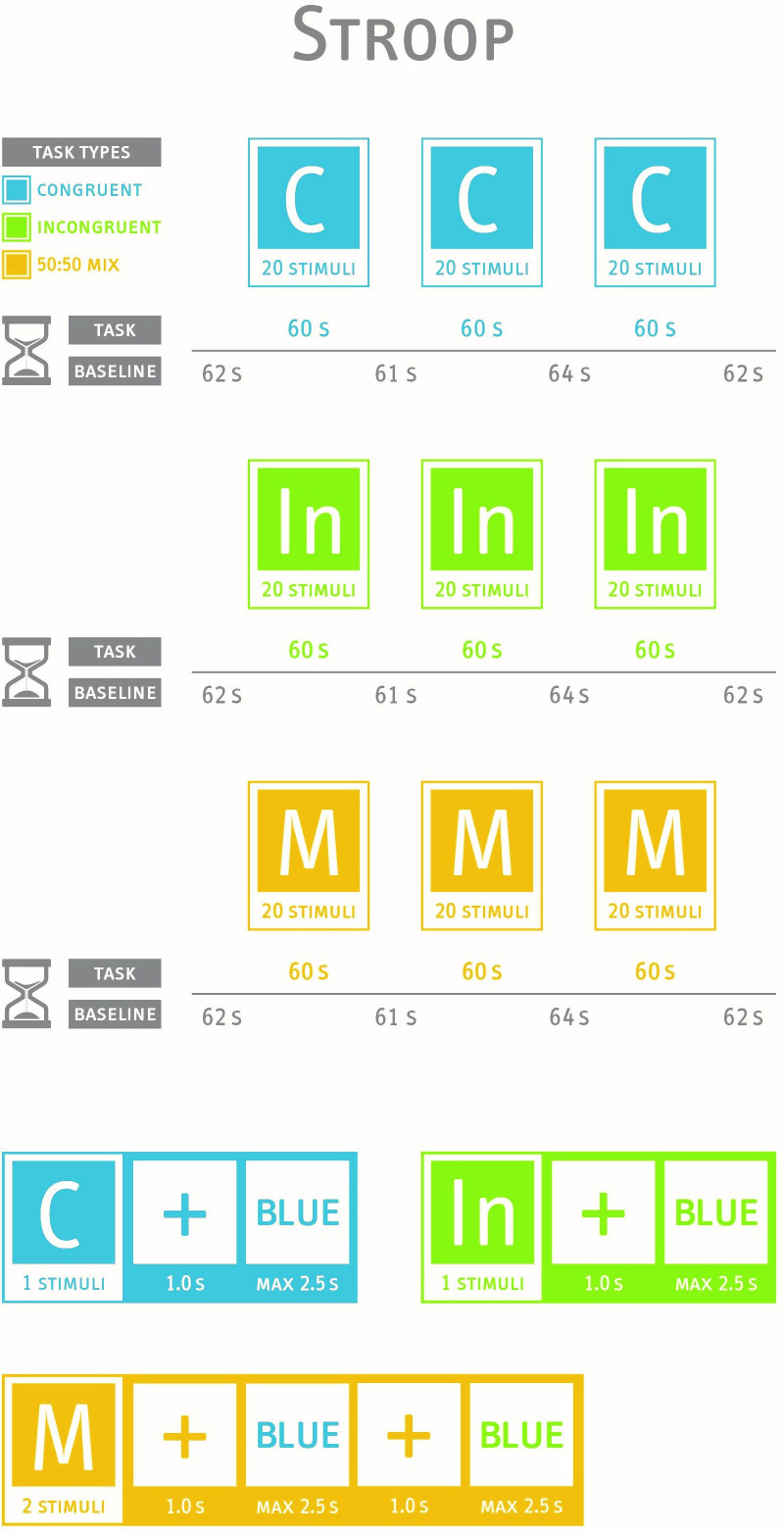
Description of the Stroop paradigm. C, congruent condition; In, incongruent condition; M, mixed condition; s, seconds.

Our Stroop test includes three experimental conditions (pure congruent, pure incongruent and mixed congruent and incongruent stimuli) with three blocks in each condition (see figure 1). Prior to each block, a baseline measurement having nearly the same length as the task blocks will be applied in order to assess a baseline for the haemodynamic activity. During the baseline measurement, participants are requested to sit still and relax. According to the recent recommendations,^17^ the duration of the baseline measurement should not be a multiple of 10 s to avoid the overlap with the Mayer waves. In each block, colour-words are consecutively presented in the middle of the computer screen. A black background was chosen to avoid overstraining the eyes. Each colour has a corresponding button. Four different colour-words will appear: ‘RED’, ‘GREEN’, ‘BLUE’ or ‘YELLOW’ in German language. In the congruent condition, the meaning of the colour-word and the ink colour matches. In the incongruent condition, the colour-word is printed in an incongruent ink colour, for example, the word ‘RED’ is presented in blue colour. Participants are instructed to identify the colour of the word by pressing the appropriate button and not reacting on the meaning of the word. In this example, the participant must press the button for the ink colour (blue). Participants should react as fast and as correct as possible. In the third block, congruent and incongruent stimuli are presented. Participants are instructed to react on the colour of the word. Although the typical clinical version of the Stroop tests includes a pure block of neutral (congruent) stimuli and a pure block of incongruent stimuli, some authors criticise this pure-block design since it has some limitations.^100^ Therefore, a mixed block design with congruent stimuli within an incongruent condition demanding the cognitive function goal-maintenance capacities will be applied. The congruent stimuli within this condition promote the inappropriate but more automatically response of reading the word and produce larger Stroop effects than pure incongruent blocks.^100^ This mixed design was already used in some studies.^103–107^ Hence, we will use a pure incongruent and a mixed block. The mixed block consists of 50% congruent and 50% incongruent stimuli. The participants should react in both variants to the colour of the word. Prior to task, the participants will be adequately familiarised with the test by performing a sufficient number of practice trials.

##### N-back

**Figure 2:**
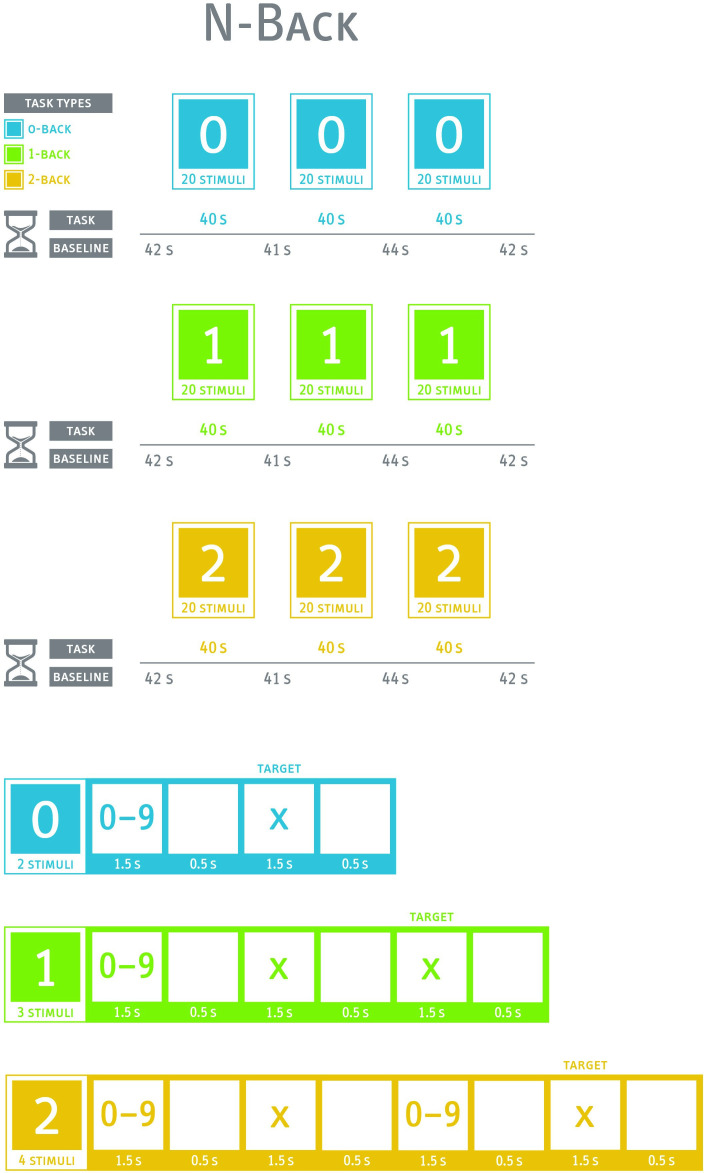
Description of N-back paradigm. 0, 0-back; 1, 1-back; 2, 2-back; s, seconds.

The N-back was first introduced by Kirchner^108^ and is a frequently used task to measure working memory capacity. In our study, three conditions (0-back, 1-back and 2-back) will be used (see figure 2). Single-digit numbers with a presentation time of 1500 ms are presented consecutively in three blocks in the middle of the screen. The interstimulus interval is 500 ms. As in the Stroop test, a baseline measurement will be included prior to each task block and after the last block.

In the 0-back condition, the participants are asked to press the target button only when the number ‘7’ appears. All other numbers must be ignored. In the 1-back condition, the participants are asked to press the target button only when two identical numbers appear in succession. In the 2-back condition, the participants are asked to press the target button only when the current number matches the second-last number displayed before. In all three conditions, 25% of stimuli are targets. As in the Stroop test, participants will be instructed to react as fast and as correct as possible. Prior to task, the participants will be adequately familiarised with the test by completing a sufficient number of practice trials.

##### Verbal fluency test

**Figure 3:**
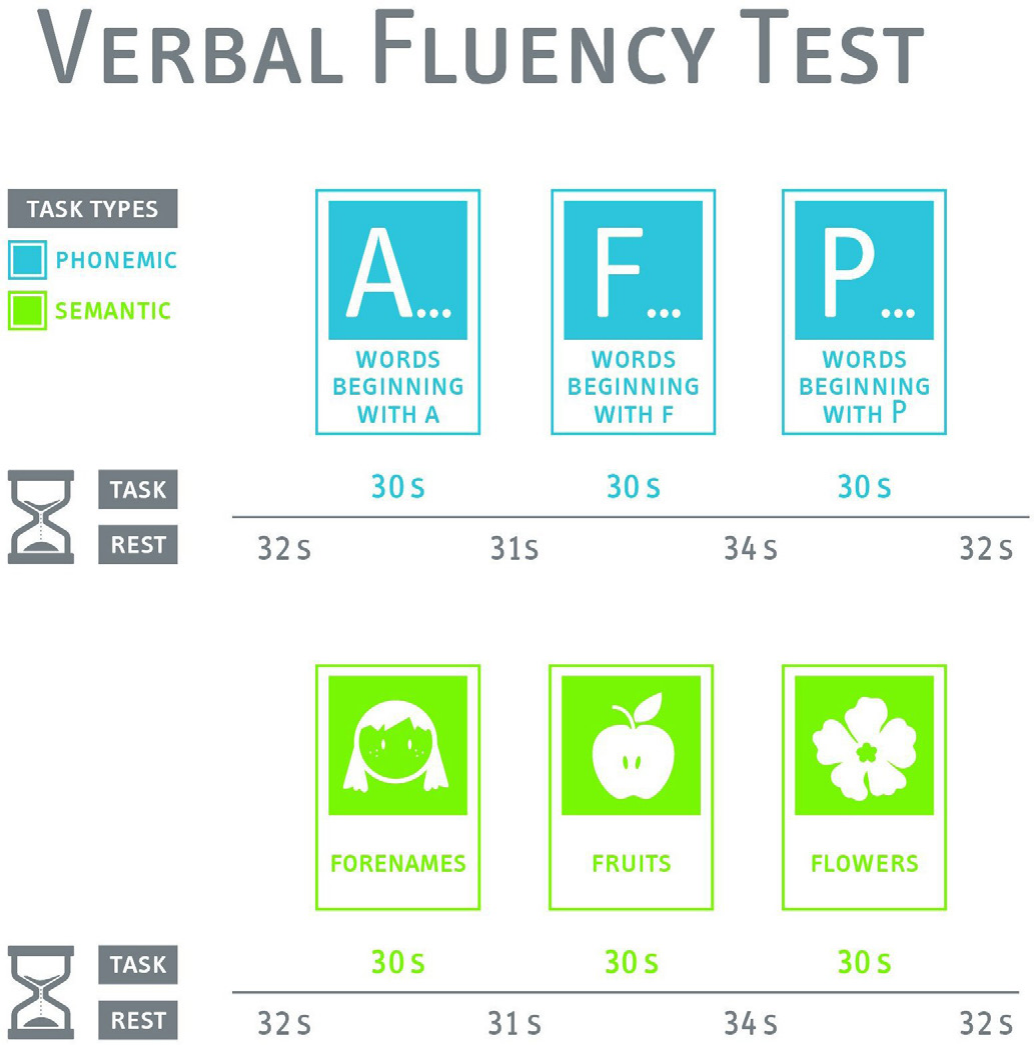
Description of verbal fluency test paradigm. s, seconds.

Verbal fluency is a cognitive domain that worsens with the development of AD.^109^ Therefore, we plan to use a VFT, which is based on the ‘Regensburger Wortflüssigkeitstest’.^110^ This test is frequently used in fNIRS studies investigating neurological diseases.^24 30 109 111^ Based on these studies, our test consists of two conditions. Both conditions will be presented three times in a row for 30 s with a resting block for 31–34 s between each block. See figure 3 for a detailed description of the VFT paradigm. During the resting blocks, the participants are requested to sit still and avoid speaking. We use a phonological (letter) and a semantic (category) condition. The instructions will be displayed on a screen to avoid instruction bias. Participants are requested to avoid movements during this test.

In the phonological condition, the participants are instructed to pronounce as many German words (nouns, verbs, adjectives) as possible beginning with a specific letter (‘A’, ‘F’, ‘P’) without using proper names, numbers, repetitions or words in different forms or with different endings. In the semantic condition, participants are instructed to pronounce as many words as possible belonging to a specific category (‘forenames’, ‘fruits’, ‘flowers’).

#### Neurophysiological assessment

##### fNIRS

**Figure 4:**
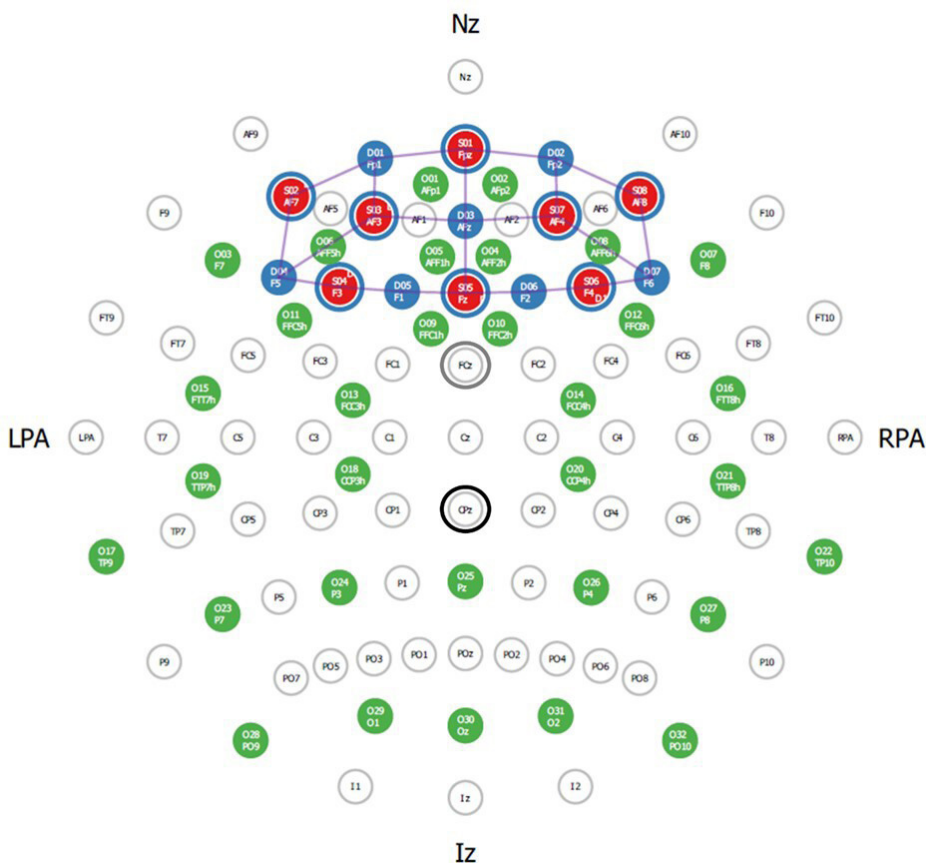
Visualization of the positions of the EEG electrodes and fNIRS optodes. IZ, inion; LPA, left preauricular point; NZ, nasion; RPA, right preauricular point.

In this study, a portable continuous wave fNIRS system (NIRSport, NIRx Medical Technologies, Glen Head, New York, USA) will be used to record cortical haemodynamics with a frequency of 10.2 Hz. The fNIRS-system consists of the following: eight light sources which emit light at wavelengths of 760 and 850 nm, eight light detectors and a short-distance detector bundle (NIRx Medical Technologies, Glen Head, New York, USA), which allows to quantify changes in extracerebral layer (ie, blood flow in the scalp). As shown in figure 4, the fNIRS optodes will be positioned according to the 10–20 EEG system^112^ by using a standardised cap (EasyCap GmbH, Herrsching, Germany). We will perform a virtual and probabilistic spatial registration using the software fOLD (fNIRS Optodes’ Location Decider)^113^ and the Broadmann atlas^114^ to assign the fNIRS measurement channels with long source-detector separation to specific brain regions (see online supplemental additional file 1 for detailed overview).

10.1136/bmjopen-2020-046879.supp1 Supplementary data

The data processing of fNIRS follows recent recommendations^17 115 116^ and will be conducted using the latest version of the ‘Homer’ software package.^117^ In brief, we will conduct the following processing steps: (1) exclude noisy channels by using enPruneChannels function, (2) convert raw light intensity changes into changes in optical density by using hmrIntensity2OD function, (3) perform motion artefact correction by using sophisticated filter methods (eg, wavlet filter—hmrMotionCorrectWavelet filtering function),^118^ (4) perform a correction for physiological artefacts such as heart beat and instrumental noise by using hmrBandpassFilt function, (5) convert optical density data of both wavelengths via the modified Beer-Lambert law into concentration changes of oxyHb and deoxyHb by using the hmrOD2Conc function and an individually calculated differential path length factor,^119^ (6) correct for extracerebral blood flow by using hmrDeconvHRRF_DriftSS function^120 121^ and (7) perform a baseline correction and calculation of block averages for oxyHb and deoxyHb changes over all trials and for each measurement channel by using hmrBlockAvg function. In the final step, the preprocessed time series of oxyHb and deoxyHb are exported and the cognition-related changes in these two chromophores are used for further statistical analysis.

##### EEG

EEG data will be acquired by the Brain Vision wireless MOVE system (Brain Products GmbH, Munich, Germany) along with Brain Vision Recorder V.1.21.0102 (Brain Products GmbH, Munich, Germany). The system will be triggered by the computer running the Presentation software. The EEG/fNIRS cap will be customised according to the 10–20 system with the provision of fNIRS optodes and 34 EEG channel slim-active electrodes (AFp1, AFp2, AFF5h, AFF1h, AFF2h, AFF6h, F7, F8, FFC5h, FFC1h, FFC2h, FFC6h, FTT7h, FCC3h, FCC4h, FTT8h, TTP7h, CCP3h, CCP4h, TTP8h, TP9, TP10, P7, P3, Pz, P4, P8, Po9, O1, Oz, PO10, Fz (reference) and ground).^80^ The electrode location will be customised in the Brain Vision Recorder workspace according to the EEG elastic cap channel position in consideration of Theta and Phi values.^122^ The impedance will be kept below 5 KΩ before EEG data recording. The cognitive tasks will be recorded following a baseline measurement of 10 min. The EEG data will be recorded at 1.000 Hz. The outcome of the results will be focused on time and frequency domain of ERPs, power spectrum analysis, source localisation and connectivity.

**Figure 5:**
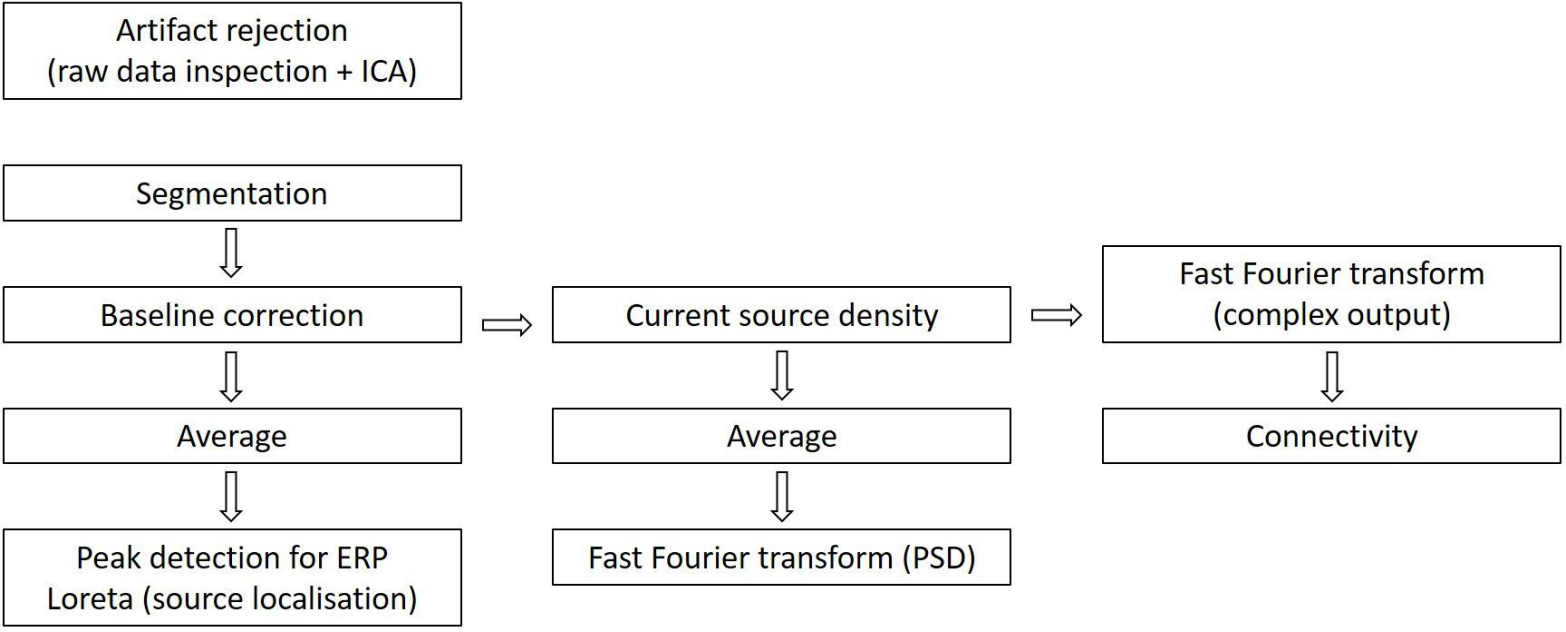
EEG data processing pipeline. ICA, independent component analysis; Loreta, low-resolution electromagnetic tomography analysis.

The EEG recorded data will be analysed by the Brain Vision Analyzer V.2.2.0 (Brain Products GmbH, Munich, Germany). The EEG postprocessing will be divided into two blocks, whereas P100, P200, P300, P600, N100 and N400 ERPs,^2^ power spectral density (PSD), connectivity and source localisation will be done for the N-back and Stroop, and PSD will be done for the VFT. The prerecorded EEG data will be resampled at 256 Hz and will be rereferenced to mean mastoids.^123^ The referenced data will be filtered for ERPs high pass at 0.1 Hz and low pass at 40 Hz with 50 Hz notch filter. For PSD, connectivity and source localisation, a low-pass filter of 85 Hz will be used. The EEG data processing pipeline after rereferencing and filtering is shown in figure 5.

##### ECG

We will record the ECG of the participants during the experimental session with an ECG medilog AR12 plus (Schiller, Baar, Switzerland). This device consists of three channels with a sampling rate of 1.000 Hz. By using this device, we can record the NN intervals in millisecond range, which enables us to accurately calculate the HRV and to precisely identify cardiac arrhythmia. The raw ECG data will be uploaded to the Medilog Darwin Analysis Software package and analysed using the Kubios premium V.3.3 software package (University of Kuopio, Finland). The HRV analysis will focus on the following parameters: root mean square of successive differences beetween adjacent NN intervals (RMSSD), percentage of adjacent NN intervals differing more than 50 ms (pNN50) and power in HF (0.15–0.4 Hz). Further time and frequency domain parameters^124 125^ will be analysed as well. In addition, we will also consider nonlinear HRV parameters to study the nonlinear dynamic properties that influence heart rate.^66^ Due to the relatively long latency of the cardiac system,^53^ the HRV (as the fNIRS parameters) will be averaged over each block of the three cognitive tasks. Following the recommendations of Laborde *et al*^49^ we plan to record and analyse the HRV at three timepoints:

- At resting state before the cognitive tasks,
- During the cognitive tasks for each block.
- After the cognitive tasks at resting state in the recovery phase.

The baseline measurement before the cognitive tasks will serve as assessment of the vagal tone of the participants. The assessment of the vagal tone is important because ‘vagally mediated HRV may serve to index the functional capacity of a set of brain structures that support the effective and efficient performance of cognitive executive function tasks including working memory and inhibitory control’.^58^ The ‘phasic HRV’ will be evaluated through comparing the HRV at rest with the values recorded during the cognitive tasks.^49^ It shows how the ANS reacts and how the participant adapts to cognitive demands. Finally, the HRV will be recorded after the cognitive tasks at resting state (recovery phase). This experimental design allows for investigation of tonic HRV for each of the three measurement points (baseline, during and after the cognitive tasks). Furthermore, we can assess the phasic HRV because we will be able to detect the change between baseline and event (‘reactivity’), the change between task and post-event (‘recovery’) and the change between baseline and post-event.^49^

### Statistical analysis

The current study is designed to investigate biomarkers that can identify participants with an increased risk for cognitive deterioration. Thereto, several biomarkers will be evaluated, as possible candidates, for an early identification of MCI. We are interested in the transition state between healthy ageing and MCI. Consequently, our statistical analysis will include:

- Analysis of the differences between HC and MCI with respect to cognitive performance and physiological parameters.
- Correlational analysis between cognitive performance and physiological parameters.
- Machine-learning-based logistic regression approach for classification into HC and MCI.

To investigate effects of group, t-test for normal distributed variables will be used, in other cases, the Mann-Whitney U test. Correction for multiple comparisons will be taken into account with false discovery rate.^126^ To investigate the correlations, we will use Pearson or Spearman correlation analysis. To control for effects of age, gender, regular physical activity and education as confounders, partial correlation will be applied. Table 1 shows the measured physiological parameters of all three cognitive tasks. Concerning ECG analysis, we will also measure the HRV in resting state before (baseline) and after the cognitive tasks (recovery).

**Table 1:** Physiological parameters of each device in all three cognitive tasks

Cognitive task	Physiological measurement device	Main parameters
Stroop (congruent and incongruent)	ECG	RMSSD, pNN50, HF (absolute and nu), non-linear parameters (SD1, SD2, ApEn, SampEn, DFA, D2, RPA, MSE)
	fNIRS	oxyHb, deoxyHb, totHb
	EEG	ERPs, PSD, connectivity and source localisation
N-back (0-, 1- and 2-back)	ECG	RMSSD, pNN50, HF (absolute and nu), non-linear parameters (SD1, SD2, ApEn, SampEn, DFA, D2, RPA, MSE)
	fNIRS	oxyHb, deoxyHb, totHb
	EEG	ERPs, PSD, connectivity and source localisation
VFT (semantic and phonological)	ECG	RMSSD, pNN50, HF (absolute and nu), non-linear parameters (SD1, SD2, ApEn, SampEn, DFA, D2, RPAn, MSE)
	fNIRS	oxyHb, deoxyHb, totHb
	EEG	PSD

ApEn, approximate entropy; D2, correlation dimension; deoxyHb, deoxygenated haemoglobin; DFA, detrended fluctuation analysis; ERPs, event related potentials; HF, high frequency power in absolute and normalised units (nu) [0.15–0.4 Hz]; MSE, mutliscale entropy; oxyHb, oxygenated haemoglobin; pNN50, NN50 divided by the total number of NN intervals; PSD, power spectral density; RMSSD, root mean square of the successive differences between adjacent normal RR intervals; RPAn, recurrence plot analysis; SampEn, sample entropy; SD1, in Poincaré plot, the standard deviation perpendicular to the line-of-identity; SD2, in Poincaré plot, the standard deviation along the line-of-identity; totHb, total haemoglobin.

The main aim of our study is to develop EEG/fNIRS/HRV measures that discriminate among HC and MCI and show that this multimodal measuring approach is promising and accurate for identification of MCI. For that purpose, we will use and adapt the machine-learning algorithm already used for bimodal approaches.^77–79 81 82 84^ In a first step, after preprocessing and artefact correction, parameters from the three modalities will be extracted and the most discriminating ones playing a role in the development of neurodegenerative diseases will be selected. Principal component analysis can help to reduce parameters to a manageable amount by removing components with the highest variance.^127^ Following parameter extraction, multimodal combining algorithm will be used for classification. To classify the signals, support vector machine (SVM) will be applied. SVM is one of the most commonly used supervised classifiers in the field of pattern recognition and has been widely adopted in many brain signal studies.^72 77–79 81 128^ SVM can define two or more classes by constructing an optimal hyperplane maximising the margin of separation between the closest data points belonging to different classes. SVM can be used in linear as well as in non-linear classification scenarios based on the kernel trick.^129^

Another aim of the study is to compare the performance of unimodal, bimodal and multimodal systems. For that reason, classification should be performed separately using different kinds of parameter sets for comparison: EEG-only, fNIRS-only, HRV-only, bimodal (EEG +fNIRS, EEG +HRV, fNIRS +HRV) and a multimodal parameter set (EEG +fNIRS +HRV).

### Data management and safety

All participants will be assigned a code independently from their group allocation ensuring their anonymity. In order to guarantee the security of all data, the personal information and the data of this study will be collected and handled exclusively by the involved researchers. All data and participants’ information will be handled according to the institutional data management policy of the University of Magdeburg. The original documents will be kept by the main investigators of the study at the Otto von Guericke University Magdeburg and the German Center for Neurodegenerative Diseases.

### Patient and public involvement

Subjects will be involved in the study as we will evaluate their acceptability for our measurement procedure. We will collect feedback on the study procedure from the participants. We will continue to work with stakeholders including the medical faculty and companies working in the field of neurodegeneration and dementia.

## Summary

Due to the demographic change and the increase in the individual life expectancy, the worldwide economic healthcare costs to treat individuals suffering from dementia will increase considerably. Currently, there is no treatment available, which would allow to heal this neurological disease. Consequently, researchers now focus more on an early diagnosis of preclinical stages of AD (eg, MCI) in order to initiate preventive actions timely. In this regard, neurophysiological signals could be promising biomarkers of such preclinical AD stages because they are, unlike behavioural performance, deemed to be less affected by learning or practice effects and provide insights into possible compensatory processes being not readily observable at the behavioural level. Hence, physiological signals can be helpful to detect changes in cognitive performance more precisely.^130^ In this context, it is assumed that the investigation of new neurophysiological biomarkers, which allows to identify MCI more easily and accurately, are necessary to better understand and monitor the disease progression.^11^

In line with the previous mentioned assumptions, the aim of this study is threefold. At first, this investigation aims to determine the differences in neurophysiological responses of HC and MCI participants. The second aim is to investigate possible neurobehavioral relationships between measures of cognitive performance and neurophysiological responses. The third aim is to elucidate whether a multimodal measurement approach can help to identify individuals with MCI more accurately and reliable than a unimodal or bimodal approach. Thereto, three complementary measuring modalities, namely, fNIRS, EEG and ECG/HRV, will be used simultaneously to assess different neurophysiological responses, which are associated with cognitive processes. To the best of our knowledge, this is the first study using these three modalities simultaneously in patients with MCI and cognitive HC. Given the explorative character of our study, our sample consists of 30 healthy controls (HC) and 30 patients with MCI whose neurophysiological signals will be recorded at a resting state and while performing three established cognitive tasks (Stroop, N-back and a VFT). We are aiming to detect certain neurophysiological parameters that are promising for an early identification of people who are at a higher risk of an overly age-related decline in cognitive performance (ie, MCI detection).

In this regard, we hypothesise that the multimodal approach improves the classification accuracy between patients with HC and MCI as compared with a unimodal or a bimodal approach. If our hypothesis is verified, this study will pave the way for further research on multimodal measurement approaches for dementia research. Such upcoming research will use a larger sample size to examine the noninvasive biomarkers characterising an early detection of nonphysiological decline in cognitive performance in more detail.

## Ethics and dissemination

Ethics approval was obtained for the study from the Ethics Committee of the Otto von Guericke University (reference: 83/19), and informed consent for participation will be obtained from all participants. Data will be shared with the scientific community no more than 1 year following completion of study and data assembly.

## Supplementary Material

Reviewer comments

Author's manuscript

## References

[1] Alzheimers Dement. 2020 Alzheimer’s disease facts and figures 2020. 10.1002/alz.12068 32157811

[2] WHO. Risk reduction of cognitive decline and dementia: WHO guidelines. Geneva: World Health Organization, 2019. 31219687

[3] Dilling H, . Internationale Klassifikation psychischer Störungen: ICD-10 Kapitel V (F); klinisch-diagnostische Leitlinien. 6., vollst. überarb. Aufl. unter Berücksichtigung der Änderungen entsprechend ICD-10-GM 2004/2008. Bern: Huber, 2008. http://d-nb.info/988424061/04

[4] Leicht H, Heinrich S, Heider D, et al. Net costs of dementia by disease stage. Acta Psychiatr Scand 2011;124:384–95. 10.1111/j.1600-0447.2011.01741.x 21838738

[5] Wallesch C-W, Förstl H, Bartels C. Demenzen. 3, unveränderte Auflage. Stuttgart, New York: Georg Thieme Verlag, 2017.

[6] Wimo A, Guerchet M, Ali G-C, et al. The worldwide costs of dementia 2015 and comparisons with 2010. Alzheimers Dement 2017;13:1–7. 10.1016/j.jalz.2016.07.150 27583652 PMC5232417

[7] Etgen T, Sander D, Bickel H, et al. Mild cognitive impairment and dementia: the importance of modifiable risk factors. Dtsch Arztebl Int 2011;108:743–50. 10.3238/arztebl.2011.0743 22163250 PMC3226957

[8] Petersen RC, Smith GE, Waring SC, et al. Mild cognitive impairment: clinical characterization and outcome. Arch Neurol 1999;56:303–8. 10.1001/archneur.56.3.303 10190820

[9] Petersen RC. Mild cognitive impairment as a diagnostic entity. J Intern Med 2004;256:183–94. 10.1111/j.1365-2796.2004.01388.x 15324362

[10] Dubois B, Hampel H, Feldman HH, et al. Preclinical Alzheimer’s disease: definition, natural history, and diagnostic criteria. Alzheimers Dement 2016;12:292–323. 10.1016/j.jalz.2016.02.002 27012484 PMC6417794

[11] Jongsiriyanyong S, Limpawattana P. Mild cognitive impairment in clinical practice: a review article. Am J Alzheimers Dis Other Demen 2018;33:500–7. 10.1177/1533317518791401 30068225 PMC10852498

[12] Kivipelto M, Mangialasche F, Ngandu T. Lifestyle interventions to prevent cognitive impairment, dementia and Alzheimer disease. Nat Rev Neurol 2018;14:653–66. 10.1038/s41582-018-0070-3 30291317

[13] Yiannopoulou KG, Papageorgiou SG. Current and future treatments in Alzheimer disease: an update. J Cent Nerv Syst Dis 2020;12:1179573520907397. 10.1177/1179573520907397 32165850 PMC7050025

[14] Atri A. The Alzheimer’s disease clinical spectrum: diagnosis and management. Med Clin North Am 2019;103:263–93. 10.1016/j.mcna.2018.10.009 30704681

[15] Annavarapu RN, Kathi S, Vadla VK. Non-Invasive imaging modalities to study neurodegenerative diseases of aging brain. J Chem Neuroanat 2019;95:54–69. 10.1016/j.jchemneu.2018.02.006 29474853

[16] Pinti P, Tachtsidis I, Hamilton A, et al. The present and future use of functional near-infrared spectroscopy (fNIRS) for cognitive neuroscience. Ann N Y Acad Sci 2020;1464:5–29. 10.1111/nyas.13948 30085354 PMC6367070

[17] Herold F, Wiegel P, Scholkmann F, et al. Applications of functional near-infrared spectroscopy (fNIRS) neuroimaging in Exercise–Cognition science: a systematic, Methodology-Focused review. JCM 2018;7:466. 10.3390/jcm7120466 PMC6306799 30469482

[18] Agbangla NF, Audiffren M, Albinet CT. Use of near-infrared spectroscopy in the investigation of brain activation during cognitive aging: a systematic review of an emerging area of research. Ageing Res Rev 2017;38:52–66. 10.1016/j.arr.2017.07.003 28755870

[19] Fantini S, Ruesch A, Kainerstorfer JM. Noninvasive optical studies of the brain. : Shi L, Alfano RR, . Neurophotonics and biomedical spectroscopy. Amsterdam: Elsevier, 2018: 25–52.

[20] Scholkmann F, Kleiser S, Metz AJ, et al. A review on continuous wave functional near-infrared spectroscopy and imaging instrumentation and methodology. Neuroimage 2014;85:6–27. 10.1016/j.neuroimage.2013.05.004 23684868

[21] Chou P-H, Lan T-H. The role of near-infrared spectroscopy in Alzheimer’s disease. Journal of Clinical Gerontology and Geriatrics 2013;4:33–6. 10.1016/j.jcgg.2013.01.002

[22] Obrig H. NIRS in clinical neurology — a ‘promising’ tool? Neuroimage 2014;85:535–46. 10.1016/j.neuroimage.2013.03.045 23558099

[23] Yeung MK, Chan AS. Functional near-infrared spectroscopy reveals decreased resting oxygenation levels and task-related oxygenation changes in mild cognitive impairment and dementia: a systematic review. J Psychiatr Res 2020;124:58–76. 10.1016/j.jpsychires.2020.02.017 32120065

[24] Katzorke A, Zeller JBM, Müller LD, et al. Decreased hemodynamic response in inferior frontotemporal regions in elderly with mild cognitive impairment. Psychiatry Res Neuroimaging 2018;274:11–18. 10.1016/j.pscychresns.2018.02.003 29472145

[25] Yeung MK, Sze SL, Woo J, et al. Altered frontal lateralization underlies the category fluency deficits in older adults with mild cognitive impairment: a near-infrared spectroscopy study. Front Aging Neurosci 2016;8:59. 10.3389/fnagi.2016.00059 27065857 PMC4809883

[26] Vermeij A, van Beek AHEA, Olde Rikkert MGM, et al. Effects of aging on cerebral oxygenation during working-memory performance: a functional near-infrared spectroscopy study. PLoS One 2012;7:e46210. 10.1371/journal.pone.0046210 23029437 PMC3460859

[27] Nguyen T, Kim M, Gwak J, et al. Investigation of brain functional connectivity in patients with mild cognitive impairment: a functional near‐infrared spectroscopy (fNIRS) study. J Biophotonics 2019;12:e201800298. 10.1002/jbio.201800298 30963713

[28] Niu H-J, Li X, Chen Y-J, et al. Reduced frontal activation during a working memory task in mild cognitive impairment: a non-invasive near-infrared spectroscopy study. CNS Neurosci Ther 2013;19:125–31. 10.1111/cns.12046 23279823 PMC6493442

[29] Yang D, Hong K-S, Yoo S-H, et al. Evaluation of neural degeneration biomarkers in the prefrontal cortex for early identification of patients with mild cognitive impairment: an fNIRS study. Front Hum Neurosci 2019;13:317. 10.3389/fnhum.2019.00317 31551741 PMC6743351

[30] Yap KH, Ung WC, Ebenezer EGM, et al. Visualizing hyperactivation in neurodegeneration based on prefrontal oxygenation: a comparative study of mild Alzheimer’s disease, mild cognitive impairment, and healthy controls. Front Aging Neurosci 2017;9:287. 10.3389/fnagi.2017.00287 28919856 PMC5585736

[31] Yoo S-H, Hong K-S. Hemodynamics analysis of patients with mild cognitive impairment during working memory tasks. Conf Proc IEEE Eng Med Biol Soc 2019:4470–3. 10.1109/EMBC.2019.8856956 31946858

[32] Cabeza R. Hemispheric asymmetry reduction in older adults: the HAROLD model. Psychol Aging 2002;17:85–100. 10.1037/0882-7974.17.1.85 11931290

[33] Michel CM, Murray MM. Towards the utilization of EEG as a brain imaging tool. Neuroimage 2012;61:371–85. 10.1016/j.neuroimage.2011.12.039 22227136

[34] Hampel H, Lista S, Teipel SJ. Perspective on future role of biological markers in clinical therapy trials of Alzheimer’s disease: a long-range point of view beyond 2020. Biochem Pharmacol 2014;88:426–49. 24275164 10.1016/j.bcp.2013.11.009

[35] Haroutunian V, Hoffman LB, Beeri MS, et al. Is there a neuropathology difference between mild cognitive impairment and dementia? Dialogues Clin Neurosci 2009;11:171–9. 19585952 10.31887/DCNS.2009.11.2/vharoutunian PMC3073531

[36] Jelic V, Kowalski J. Evidence-Based evaluation of diagnostic accuracy of resting EEG in dementia and mild cognitive impairment. Clin EEG Neurosci 2009;40:129–42. 10.1177/155005940904000211 19534305

[37] Scheff SW, Price DA, Schmitt FA, et al. Hippocampal synaptic loss in early Alzheimer’s disease and mild cognitive impairment. Neurobiol Aging 2006;27:1372–84. 10.1016/j.neurobiolaging.2005.09.012 16289476

[38] Al-Qazzaz NK, Ali SHBM, Ahmad SA, et al. Role of EEG as biomarker in the early detection and classification of dementia. Sci World J 2014;2014:906038 10.1155/2014/906038 25093211 PMC4100295

[39] Choi J, Ku B, You YG, et al. Resting-State prefrontal EEG biomarkers in correlation with MMSE scores in elderly individuals. Sci Rep 2019;9:10468. 10.1038/s41598-019-46789-2 31320666 PMC6639387

[40] Gouw AA, Stam CJ. Electroencephalography in the differential diagnosis of dementia. Epileptologie 2016;33:173 https://research.vumc.nl/en/publications/electroencephalography-in-the-differential-diagnosis-of-dementia

[41] Horvath A, Szucs A, Csukly G. EEG and ERP biomarkers of Alzheimer rsquo S disease a critical review. Frontiers in Bioscience 2018;23:183–220. 10.2741/4587 28930543

[42] Yang S, Bornot JMS, Wong-Lin K, et al. M/EEG-based bio-markers to predict the MCI and Alzheimer’s disease: a review from the ML perspective. IEEE Trans Biomed Eng 2019;66:2924–35. 10.1109/TBME.2019.2898871 30762522

[43] Sur S, Sinha VK. Event-Related potential: an overview. Ind Psychiatry J 2009;18:70–3. 10.4103/0972-6748.57865 21234168 PMC3016705

[44] Sakkalis V. Review of advanced techniques for the estimation of brain connectivity measured with EEG/MEG. Comput Biol Med 2011;41:1110–7. 10.1016/j.compbiomed.2011.06.020 21794851

[45] Sarmukadam K, Sharpley CF, Bitsika V, et al. A review of the use of EEG connectivity to measure the neurological characteristics of the sensory features in young people with autism. Rev Neurosci 2019;30:497–510. 10.1515/revneuro-2018-0070 30269108

[46] Schoffelen J-M, Gross J. Source connectivity analysis with MEG and EEG. Hum Brain Mapp 2009;30:1857–65. 10.1002/hbm.20745 19235884 PMC6870611

[47] Schapkin SA, Raggatz J, Hillmert M. Eeg correlates of cognitive load in a multiple choice reaction task. Acta Neurobiol Exp 2020;80:76–89. 32214277

[48] Schomer DL, Lopes da Silva FH, . Niedermeyer’s electroencephalography: Basic principles, clinical applications, and related fields. 7th edn. New York: Oxford University Press, 2017.

[49] Laborde S, Mosley E, Thayer JF. Heart rate variability and cardiac vagal tone in psychophysiological research - Recommendations for experiment planning, data analysis, and data reporting. Front Psychol 2017;8:213. 10.3389/fpsyg.2017.00213 28265249 PMC5316555

[50] McCraty R, Shaffer F. Heart rate variability: new perspectives on physiological mechanisms, assessment of self-regulatory capacity, and health risk. Glob Adv Health Med 2015;4:46–61. 10.7453/gahmj.2014.073 PMC4311559 25694852

[51] Billman GE. The LF/HF ratio does not accurately measure cardiac sympatho-vagal balance. Front Physiol 2013;4. 10.3389/fphys.2013.00026 PMC3576706 23431279

[52] Draghici AE, Taylor JA. The physiological basis and measurement of heart rate variability in humans. J Physiol Anthropol 2016;35:22. 10.1186/s40101-016-0113-7 27680542 PMC5039876

[53] Shaffer F, McCraty R, Zerr CL. A healthy heart is not a metronome: an integrative review of the heart's anatomy and heart rate variability. Front Psychol 2014;5:1040. 10.3389/fpsyg.2014.01040 25324790 PMC4179748

[54] Fatisson J, Oswald V, Lalonde F. Influence diagram of physiological and environmental factors affecting heart rate variability: an extended literature overview. Heart Int 2016;11:heartint.500023. 10.5301/heartint.5000232 PMC5056628 27924215

[55] Sammito S, Böckelmann I. Factors influencing heart rate variability. ICFJ 2016;6. 10.17987/icfj.v6i0.242 PMC11333334 39165281

[56] Thayer JF, Yamamoto SS, Brosschot JF. The relationship of autonomic imbalance, heart rate variability and cardiovascular disease risk factors. Int J Cardiol 2010;141:122–31. 10.1016/j.ijcard.2009.09.543 19910061

[57] Thayer JF, Lane RD. A model of neurovisceral integration in emotion regulation and dysregulation. J Affect Disord 2000;61:201–16. 10.1016/S0165-0327(00)00338-4 11163422

[58] Thayer JF, Hansen AL, Saus-Rose E, et al. Heart rate variability, prefrontal neural function, and cognitive performance: the neurovisceral integration perspective on self-regulation, adaptation, and health. Ann Behav Med 2009;37:141–53. 10.1007/s12160-009-9101-z 19424767

[59] Thayer JF, Lane RD. Claude Bernard and the heart–brain connection: further elaboration of a model of neurovisceral integration. Neurosci Biobehav Rev 2009;33:81–8. 10.1016/j.neubiorev.2008.08.004 18771686

[60] Forte G, Favieri F, Casagrande M. And cognitive function: a systematic review. Front Neurosci 2019;13:710. 31354419 10.3389/fnins.2019.00710 PMC6637318

[61] Eggenberger P, Annaheim S, Kündig KA, et al. Heart rate variability mainly relates to cognitive executive functions and improves through Exergame training in older adults: a secondary analysis of a 6-month randomized controlled trial. Front Aging Neurosci 2020;12:197. 10.3389/fnagi.2020.00197 32760267 PMC7373948

[62] Grässler B, Hökelmann A, Cabral RH. Resting heart rate variability as a possible marker of cognitive decline. Kinesiology 2020;52:72–84. 10.26582/k.52.1.9

[63] Zeki Al Hazzouri A, Haan MN, Deng Y, et al. Reduced heart rate variability is associated with worse cognitive performance in elderly Mexican Americans. Hypertension 2014;63:181–7. 10.1161/HYPERTENSIONAHA.113.01888 24144650 PMC4045649

[64] Kim DH, Lipsitz LA, Ferrucci L, et al. Association between reduced heart rate variability and cognitive impairment in older disabled women in the community: Women’s health and aging study I. J Am Geriatr Soc 2006;54:1751–7. 10.1111/j.1532-5415.2006.00940.x 17087704 PMC2276586

[65] da Silva VP, Ramalho Oliveira BR, Tavares Mello RG, et al. Heart rate variability indexes in dementia: a systematic review with a quantitative analysis. Curr Alzheimer Res 2018;15:80–8. 10.2174/1567205014666170531082352 28558638

[66] Delliaux S, Delaforge A, Deharo J-C, et al. Mental workload alters heart rate variability, lowering non-linear dynamics. Front Physiol 2019;10:565. 10.3389/fphys.2019.00565 31156454 PMC6528181

[67] Mukherjee S, Yadav R, Yung I, et al. Sensitivity to mental effort and test-retest reliability of heart rate variability measures in healthy seniors. Clin Neurophysiol 2011;122:2059–66. 10.1016/j.clinph.2011.02.032 21459665 PMC3132243

[68] Sassi R, Cerutti S, Lombardi F, et al. Advances in heart rate variability signal analysis: joint position statement by the e-Cardiology ESC Working group and the European heart rhythm association co-endorsed by the Asia Pacific heart rhythm Society. Europace 2015;17:1341–53. 10.1093/europace/euv015 26177817

[69] Young H, Benton D. We should be using nonlinear indices when relating heart-rate dynamics to cognition and mood. Sci Rep 2015;5:16619. 10.1038/srep16619 26565560 PMC4643265

[70] Fernandes de Godoy M, Department of Cardiology and Cardiovascular Surgery, S鉶 Jose do Rio Preto Medical School, Famerp, Brazil. Nonlinear analysis of heart rate variability: a comprehensive review. J Cardiol Ther 2016;3:528–33. 10.17554/j.issn.2309-6861.2016.03.101-4

[71] Bari V, Calcagnile P, Molteni E, et al. From neurovascular coupling to neurovascular cascade: a study on neural, autonomic and vascular transients in attention. Physiol Meas 2012;33:1379–97. 10.1088/0967-3334/33/8/1379 22828121

[72] Ha U, Lee Y, Kim H. A wearable EEG-HEG-HRV multimodal system with simultaneous monitoring of tES for mental health management. IEEE Trans Biomed Circuits Syst 2015;9:758–66. 26742142 10.1109/TBCAS.2015.2504959

[73] Chiarelli AM, Zappasodi F, Di Pompeo F, et al. Simultaneous functional near-infrared spectroscopy and electroencephalography for monitoring of human brain activity and oxygenation: a review. Neurophotonics 2017;4:1. 10.1117/1.NPh.4.4.041411 PMC5566595 28840162

[74] Alba G, Vila J, Rey B, et al. The relationship between heart rate variability and electroencephalography functional connectivity variability is associated with cognitive flexibility. Front Hum Neurosci 2019;13:64. 10.3389/fnhum.2019.00064 30858800 PMC6397840

[75] Hillmert M, Bergmüller A, Minow A. Psychophysiologische Beanspruchungskorrelate während kognitiver Belastung. Zbl Arbeitsmed 2020:1–15.

[76] Brugnera A, Adorni R, Compare A, et al. Cortical and autonomic patterns of emotion experiencing during a recall task. J Psychophysiol 2018;32:53–63. 10.1027/0269-8803/a000183

[77] Aghajani H, Garbey M, Omurtag A. Measuring mental workload with EEG+fNIRS. Front Hum Neurosci 2017;11:359. 10.3389/fnhum.2017.00359 28769775 PMC5509792

[78] Ahn JW, Ku Y, Kim HC. A novel wearable EEG and ECG recording system for stress assessment. Sensors 2019;19:1991. 10.3390/s19091991 PMC6539530 31035399

[79] Al-Shargie F, Tang TB, Kiguchi M. Stress assessment based on decision fusion of EEG and fNIRS signals. IEEE Access 2017;5:19889–96. 10.1109/ACCESS.2017.2754325

[80] Li R, Nguyen T, Potter T, et al. Dynamic cortical connectivity alterations associated with Alzheimer’s disease: An EEG and fNIRS integration study. Neuroimage 2019;21:101622. 10.1016/j.nicl.2018.101622 30527906 PMC6411655

[81] Li R, Potter T, Huang W, et al. Enhancing performance of a hybrid EEG-fNIRS system using channel selection and early temporal features. Front Hum Neurosci 2017;11:462. 10.3389/fnhum.2017.00462 28966581 PMC5605645

[82] Liu Y, Ayaz H, Shewokis PA. Multisubject “learning” for mental workload classification using concurrent EEG, fNIRS, and physiological measures. Front Hum Neurosci 2017;11:389. 10.3389/fnhum.2017.00389 28798675 PMC5529418

[83] Morioka H, Kanemura A, Morimoto S, et al. Decoding spatial attention by using cortical currents estimated from electroencephalography with near-infrared spectroscopy prior information. Neuroimage 2014;90:128–39. 10.1016/j.neuroimage.2013.12.035 24374077

[84] Shin J, Kwon J, Im C-H. A ternary hybrid EEG-NIRS brain-computer interface for the classification of brain activation patterns during mental arithmetic, motor imagery, and idle state. Front Neuroinform 2018;12:5. 10.3389/fninf.2018.00005 29527160 PMC5829061

[85] Nicolini P, Ciulla MM, Malfatto G, et al. Autonomic dysfunction in mild cognitive impairment: evidence from power spectral analysis of heart rate variability in a cross-sectional case-control study. PLoS ONE 2014;9:e96656. 10.1371/journal.pone.0096656 24801520 PMC4011966

[86] Lai C-L, Lin R-T, Liou L-M, et al. The role of event-related potentials in cognitive decline in Alzheimer’s disease. Clin Neurophysiol 2010;121:194–9. 10.1016/j.clinph.2009.11.001 20005164

[87] Faul F, Erdfelder E, Lang A-G, et al. G*Power 3: a flexible statistical power analysis program for the social, behavioral, and biomedical sciences. Behav Res Method 2007;39:175–91. 10.3758/BF03193146 17695343

[88] Ahn S, Nguyen T, Jang H, et al. Exploring neuro-physiological correlates of drivers’ mental fatigue caused by sleep deprivation using simultaneous EEG, ECG, and fNIRS data. Front Hum Neurosci 2016;10:219. 10.3389/fnhum.2016.00219 27242483 PMC4865510

[89] Sperling RA, Aisen PS, Beckett LA, et al. Toward defining the preclinical stages of Alzheimer’s disease: recommendations from the National Institute on Aging-Alzheimer’s association workgroups on diagnostic guidelines for Alzheimer’s disease. Alzheimers Dement 2011;7:280–92. 10.1016/j.jalz.2011.03.003 21514248 PMC3220946

[90] Morris JC, Mohs RC, Rogers H, et al. Consortium to establish a Registry for Alzheimer’s disease (CERAD) clinical and neuropsychological assessment of Alzheimer’s disease. Psychopharmacol Bull 1988;24:641–52. 3249766

[91] Barth S, Schönknecht P, Pantel J, et al. Neuropsychologische profile in Der Demenzdiagnostik: Eine Untersuchung MIT Der CERAD-NP-Testbatterie. Fortschr Neurol Psychiatr 2005;73:568–76. 10.1055/s-2004-830249 16217697

[92] Huy C, Schneider S. Instrument für die Erfassung der physischen Aktivität bei Personen im mittleren und höheren Erwachsenenalter. Entwicklung, Prüfung und Anwendung des “German-PAQ-50+”. Z Gerontol Geriatr 2008;41:209–16. 10.1007/s00391-007-0474-y 18327696

[93] Ware JE. Sf-36 health survey update. Spine 2000;25:3130–9. 10.1097/00007632-200012150-00008 11124729

[94] Buysse DJ, Reynolds CF, Monk TH, et al. The Pittsburgh sleep quality index: a new instrument for psychiatric practice and research. Psychiatry Res 1989;28:193–213. 10.1016/0165-1781(89)90047-4 2748771

[95] Haftenberger M, Heuer T, Heidemann C, et al. Relative validation of a food frequency questionnaire for national health and nutrition monitoring. Nutr J 2010;9:36. 10.1186/1475-2891-9-36 20840739 PMC2945984

[96] Fahrenberg J, Schönpflug W. Methoden Der Aktivierungsforschung. Bern, Stuttgart, Wien: Hans Huber, 1969.

[97] Hancock PA, . Human mental workload. Amsterdam: North-Holland, 1988.

[98] Longo L. On the reliability, validity and sensitivity of three mental workload assessment techniques for the evaluation of instructional designs: a case study in a Third-level course. in: proceedings of the 10th International Conference on computer supported education. SCITEPRESS - Science and Technology Publications, 2018: 166–78.

[99] Stroop JR. Studies of interference in serial verbal reactions. Journal of Psychol 1935;18:643–62. 10.1037/h0054651

[100] Bélanger S, Belleville S, Gauthier S. Inhibition impairments in Alzheimer’s disease, mild cognitive impairment and healthy aging: effect of Congruency proportion in a Stroop task. Neuropsychologia 2010;48:581–90. 10.1016/j.neuropsychologia.2009.10.021 19879885

[101] Sun J, Sun B, Zhang L, et al. Correlation between hemodynamic and electrophysiological signals dissociates neural correlates of conflict detection and resolution in a Stroop task: a simultaneous near-infrared spectroscopy and event-related potential study. J Biomed Opt 2013;18:96014. 10.1117/1.JBO.18.9.096014 19895124

[102] Mansouri C, Kashou NH. Spatial sensitivity of near-infrared spectroscopic brain imaging based on three-dimensional Monte Carlo modeling. Conf Proc IEEE Eng Med Biol Soc 2009:1457–60. 10.1109/IEMBS.2009.5332919 19963751

[103] Badzakova-Trajkov G, Barnett KJ, Waldie KE, et al. An Erp investigation of the Stroop task: the role of the cingulate in attentional allocation and conflict resolution. Brain Res 2009;1253:139–48. 10.1016/j.brainres.2008.11.069 19084509

[104] Egner T, Hirsch J. The neural correlates and functional integration of cognitive control in a Stroop task. Neuroimage 2005;24:539–47. 10.1016/j.neuroimage.2004.09.007 15627596

[105] Compton RJ, Huber E, Levinson AR, et al. Is “conflict adaptation” driven by conflict? Behavioral and EEG evidence for the underappreciated role of congruent trials. Psychophysiology 2012;49:583–9. 10.1111/j.1469-8986.2012.01354.x 22332754

[106] Satish P, Muralikrishnan K, Balasubramanian K. Heart rate variability changes during stroop color and word test among genders. Indian J Physiol Pharmacol 2015;59:9–15. 26571978

[107] Baumert A, Buchholz N, Zinkernagel A, et al. Causal underpinnings of working memory and Stroop interference control: testing the effects of anodal and cathodal tDCS over the left DLPFC. Cogn Affect Behav Neurosci 2020;20:34–48. 10.3758/s13415-019-00726-y 31183619 PMC7012981

[108] Kirchner WK. Age differences in short-term retention of rapidly changing information. J Exp Psychol 1958;55:352–8. 10.1037/h0043688 13539317

[109] Herrmann MJ, Langer JBM, Jacob C, et al. Reduced prefrontal oxygenation in Alzheimer disease during verbal fluency tasks. Am J Geriatr Psychiatry 2008;16:125–35. 10.1097/JGP.0b013e3180cc1fbc 17998307

[110] Aschenbrenner S, Tucha O, Lange KW. Regensburger Wortflüssigkeits-Test: RWT. Handanweisung. Göttingen: Hogrefe Verl. für Psychologie, 2000.

[111] Heinzel S, Metzger FG, Ehlis A-C, et al. Age and vascular burden determinants of cortical hemodynamics underlying verbal fluency. PLoS One 2015;10:e0138863. 10.1371/journal.pone.0138863 26394050 PMC4578891

[112] Jurcak V, Tsuzuki D, Dan I. 10/20, 10/10, and 10/5 systems revisited: their validity as relative head-surface-based positioning systems. Neuroimage 2007;34:1600–11. 10.1016/j.neuroimage.2006.09.024 17207640

[113] Zimeo Morais GA, Balardin JB, Sato JR. fNIRS optodes’ location Decider (fOLD): a toolbox for probe arrangement guided by brain regions-of-interest. Sci Rep 2018;8:3341. 10.1038/s41598-018-21716-z 29463928 PMC5820343

[114] Rorden C, Brett M. Stereotaxic display of brain lesions. Behav Neurol 2000;12:191–200. 10.1155/2000/421719 11568431

[115] Tachtsidis I, Scholkmann F. False positives and false negatives in functional near-infrared spectroscopy: issues, challenges, and the way forward. Neurophotonics 2016;3:030401. 10.1117/1.NPh.3.3.030401 27054143 PMC4791590

[116] Pinti P, Scholkmann F, Hamilton A, et al. Current status and issues regarding pre-processing of fNIRS neuroimaging data: an investigation of diverse signal filtering methods within a general linear model framework. Front Hum Neurosci 2018;12:505. 10.3389/fnhum.2018.00505 30687038 PMC6336925

[117] Huppert TJ, Diamond SG, Franceschini MA, et al. HomER: a review of time-series analysis methods for near-infrared spectroscopy of the brain. Appl Opt 2009;48:D280–98. 10.1364/AO.48.00D280 19340120 PMC2761652

[118] Brigadoi S, Ceccherini L, Cutini S, et al. Motion artifacts in functional near-infrared spectroscopy: a comparison of motion correction techniques applied to real cognitive data. Neuroimage 2014;85:181–91. 10.1016/j.neuroimage.2013.04.082 23639260 PMC3762942

[119] Scholkmann F, Wolf M. General equation for the differential pathlength factor of the frontal human head depending on wavelength and age. J Biomed Opt 2013;18:105004. 10.1117/1.JBO.18.10.105004 24121731

[120] Jahani S, Fantana AL, Harper D, et al. fNIRS can robustly measure brain activity during memory encoding and retrieval in healthy subjects. Sci Rep 2017;7:9533. 10.1038/s41598-017-09868-w 28842618 PMC5572719

[121] von Lühmann A, Li X, Müller K-R, et al. Improved physiological noise regression in fNIRS: a multimodal extension of the general linear model using temporally embedded canonical correlation analysis. Neuroimage 2020;208:116472. 10.1016/j.neuroimage.2019.116472 31870944 PMC7703677

[122] Kropotov ID. Quantitative EEG, event-related potentials and neurotherapy. 1st edn. Amsterdam, Boston, London: Elsevier/Academic, 2009. http://site.ebrary.com/lib/alltitles/docDetail.action?docID=10254769

[123] Lei X, Liao K. Understanding the influences of EEG reference: a large-scale brain network perspective. Front Neurosci 2017;11:205. 10.3389/fnins.2017.00205 28450827 PMC5390022

[124] Malik M. Heart rate variability: standards of measurement, physiological interpretation, and clinical use. Circulation 1996;93:1043–65. 8598068

[125] Sammito S, Thielmann B, Seibt R, et al. Guideline for the application of heart rate and heart rate variability in occupational medicine and occupational science. ASUI 2015;2015. 10.17147/ASUI.2015-06-09-03 PMC11089808 38741189

[126] Chen S-Y, Feng Z, Yi X. A general introduction to adjustment for multiple comparisons. J Thorac Dis 2017;9:1725–9. 10.21037/jtd.2017.05.34 28740688 PMC5506159

[127] Dahne S, Bieszmann F, Samek W, et al. Multivariate machine learning methods for fusing multimodal functional neuroimaging data. Proc IEEE Inst Electr Electron Eng 2015;103:1507–30. 10.1109/JPROC.2015.2425807

[128] Hong K-S, Khan MJ, Hong MJ. Feature extraction and classification methods for hybrid fNIRS-EEG brain-computer interfaces. Front Hum Neurosci 2018;12:246. 10.3389/fnhum.2018.00246 30002623 PMC6032997

[129] Murty MN, Raghava R. Support vector machines and perceptrons. Cham: Springer International Publishing, 2016.

[130] Ranchet M, Morgan JC, Akinwuntan AE, et al. Cognitive workload across the spectrum of cognitive impairments: a systematic review of physiological measures. Neurosci Biobehav Rev 2017;80:516–37. 10.1016/j.neubiorev.2017.07.001 28711663

