# Supplementary material for: Multimodal measurement approach to identify individuals with mild cognitive impairment: study protocol for a cross-sectional trial
Source: BMJ Open. 2021 May 25;11(5):e046879. doi: 10.1136/bmjopen-2020-046879 (PMC8154928; doi:10.1136/bmjopen-2020-046879)
Supplement: Supplementary data [file bmjopen-2020-046879supp001.pdf]

Position	Source	Detector	X (mm)	Y (mm)	Z (mm)	Landmark	Specificity (%)	Distance (mm)
F4-F2	F4	F2	30	40	41	9 - Dorsolateral prefrontal cortex	68,36664315	30
						46 - Dorsolateral prefrontal cortex	22,39597886	
						8 - Includes Frontal eye fields	5,568764573	
F4-F6	F4	F6	46	38	24	45 - pars triangularis Broca's area	70,67253504	30
						46 - Dorsolateral prefrontal cortex	23,4516285	
AF8-F6	AF8	F6	48	46	5	45 - pars triangularis Broca's area	43,87699256	33
						46 - Dorsolateral prefrontal cortex	43,17605645	
						47 - Inferior prefrontal gyrus	5,07367444	
AF8-Fp2	AF8	Fp2	34	59	-2	10 - Frontopolar area	31,0841122	30
						11 - Orbitofrontal area	30,472822	
						46 - Dorsolateral prefrontal cortex	20,4016318	
AF4-F2	AF4	F2	22	52	33	9 - Dorsolateral prefrontal cortex	51,5248078	44
						46 - Dorsolateral prefrontal cortex	26,41541438	
						10 - Frontopolar area	18,40538603	
AF4-F6	AF4	F6	40	50	16	46 - Dorsolateral prefrontal cortex	47,36091434	45
						45 - pars triangularis Broca's area	30,60393436	
						10 - Frontopolar area	18,97467131	
AF4-Fp2	AF4	Fp2	25	63	9	10 - Frontopolar area	68,78124608	28
						11 - Orbitofrontal area	21,56708753	

						46 - Dorsolateral prefrontal cortex	6,609548215	
AF4-AFz	AF4	AFz	13	61	24	10 - Frontopolar area	72,46582734	36
						9 - Dorsolateral prefrontal cortex	16,97337784	
						46 - Dorsolateral prefrontal cortex	7,97503285	
Fpz-Fp2	Fpz	Fp2	13	67	0	10 - Frontopolar area	54,45817975	31
						11 - Orbitofrontal area	44,84702617	
Fpz-Afz	Fpz	AFz	1	64	14	10 - Frontopolar area	87,47712753	40
						9 - Dorsolateral prefrontal cortex	5,080149043	
Fpz-Fp1	Fpz	Fp1	-12	67	0	10 - Frontopolar area	54,49683736	30
						11 - Orbitofrontal area	44,89777168	
AF3-AFz	AF3	AFz	-12	62	23	10 - Frontopolar area	75,76225222	36
						9 - Dorsolateral prefrontal cortex	14,5484687	
						46 - Dorsolateral prefrontal cortex	8,053792322	
AF3-Fp1	AF3	Fp1	-24	63	9	10 - Frontopolar area	69,63399373	27
						11 - Orbitofrontal area	20,11557888	
						46 - Dorsolateral prefrontal cortex	8,793079404	
AF3-F5	AF3	F5	-39	50	17	46 - Dorsolateral prefrontal cortex	49,33992409	44
						45 - pars triangularis Broca's area	32,11689876	
						10 - Frontopolar area	16,03613047	

AF3-F1	AF3	F1	-23	52	32	9 - Dorsolateral prefrontal cortex	48,43964622	44
						46 - Dorsolateral prefrontal cortex	32,04248075	
						10 - Frontopolar area	16,88767141	
AF7-Fp1	AF7	Fp1	-33	59	-2	11 - Orbitofrontal area	32,71174196	30
						46 - Dorsolateral prefrontal cortex	25,27140585	
						10 - Frontopolar area	25,12699295	
						47 - Inferior prefrontal gyrus	13,10460546	
AF7-F5	AF7	F5	-47	46	6	45 - pars triangularis Broca's area	48,7862379	33
						46 - Dorsolateral prefrontal cortex	43,20372761	
F3-F5	F3	F5	-46	39	26	45 - pars triangularis Broca's area	72,56408005	29
						46 - Dorsolateral prefrontal cortex	21,83837451	
F3-F1	F3	F1	-31	39	41	9 - Dorsolateral prefrontal cortex	66,60628263	29
						46 - Dorsolateral prefrontal cortex	24,84615413	
Fz-F2	Fz	F2	10	41	50	9 - Dorsolateral prefrontal cortex	68,93306313	29
						8 - Includes Frontal eye fields	28,89129929	
Fz-AFz	Fz	AFz	2	50	39	9 - Dorsolateral prefrontal cortex	61,76828691	40
						10 - Frontopolar area	20,26301996	
						8 - Includes Frontal eye fields	12,14994939	
						32 - Dorsal anterior cingulate cortex	5,083608014	

Fz-F1	Fz	F1	-9	41	50	9 - Dorsolateral prefrontal cortex	63,16113601	29
						8 - Includes Frontal eye fields	34,73195285	

Please note that this output was generated using FOLD-software (G. A. Zimeo Morais, J. B. Balardin, and J. R. Sato, “fNIRS Optodes' Location Decider (fOLD): A toolbox for probe arrangement guided by brain regions-of-interest,” Scientific reports, vol. 8, no. 1, p. 3341, 2018.).
