# Supplementary material for: Multimodal measurement approach to identify individuals with mild cognitive impairment: study protocol for a cross-sectional trial
Source: BMJ Open. 2021 May 25;11(5):e046879. doi: 10.1136/bmjopen-2020-046879 (PMC8154928; doi:10.1136/bmjopen-2020-046879)
Supplement: Author's manuscript [file bmjopen-2020-046879.draft_revisions.pdf]

BMJ Open is committed to open peer review. As part of this commitment we make the peer review history of every article we publish publicly available.

When an article is published we post the peer reviewers' comments and the authors' responses online. We also post the versions of the paper that were used during peer review. These are the versions that the peer review comments apply to.

The versions of the paper that follow are the versions that were submitted during the peer review process. They are not the versions of record or the final published versions. They should not be cited or distributed as the published version of this manuscript.

BMJ Open is an open access journal and the full, final, typeset and author-corrected version of record of the manuscript is available on our site with no access controls, subscription charges or pay-per-view fees (http://bmjopen.bmj.com).

If you have any questions on BMJ Open's open peer review process please email info.bmjopen@bmj.com

BMJ Open

Multimodal measurement approach to identify individuals with mild cognitive impairment: study protocol for a cross-sectional trial

Journal:	*BMJ Open*
Manuscript ID	bmjopen-2020-046879
Article Type:	Protocol
Date Submitted by the Author:	11-Nov-2020
Complete List of Authors:	Grässler, Bernhard; Otto von Guericke Universitat Magdeburg, Institute for Sport Science<br>Herold, Fabian; German Centre for Neurodegenerative Diseases Site Magdeburg, Research Group Neuroprotection<br>Dordevic, Milos; German Centre for Neurodegenerative Diseases Site Magdeburg, Research Group Neuroprotection<br>Gujar, Tariq; Otto von Guericke Universitat Magdeburg, Institute for Sport Science<br>Darius, Sabine; Otto von Guericke University Medical Faculty, Occupational Medicine<br>Böckelmann, Irina; Otto von Guericke University Medical Faculty, Occupational Medicine<br>Müller, Notger; German Centre for Neurodegenerative Diseases Site Magdeburg, Research Group Neuroprotection<br>Hökelmann, Anita; Otto von Guericke Universitat Magdeburg, Institute for Sport Science
Keywords:	CARDIOLOGY, MENTAL HEALTH, Physiology < NATURAL SCIENCE DISCIPLINES, Dementia < NEUROLOGY, Neurophysiology < NEUROLOGY

SCHOLARONE™
Manuscripts

I, the Submitting Author has the right to grant and does grant on behalf of all authors of the Work (as defined in the below author licence), an exclusive licence and/or a non-exclusive licence for contributions from authors who are: i) UK Crown employees; ii) where BMJ has agreed a CC-BY licence shall apply, and/or iii) in accordance with the terms applicable for US Federal Government officers or employees acting as part of their official duties; on a worldwide, perpetual, irrevocable, royalty-free basis to BMJ Publishing Group Ltd ("BMJ") its licensees and where the relevant Journal is co-owned by BMJ to the co-owners of the Journal, to publish the Work in this journal and any other BMJ products and to exploit all rights, as set out in our licence.

The Submitting Author accepts and understands that any supply made under these terms is made by BMJ to the Submitting Author unless you are acting as an employee on behalf of your employer or a postgraduate student of an affiliated institution which is paying any applicable article publishing charge ("APC") for Open Access articles. Where the Submitting Author wishes to make the Work available on an Open Access basis (and intends to pay the relevant APC), the terms of reuse of such Open Access shall be governed by a Creative Commons licence – details of these licences and which Creative Commons licence will apply to this Work are set out in our licence referred to above.

Other than as permitted in any relevant BMJ Author's Self Archiving Policies, I confirm this Work has not been accepted for publication elsewhere, is not being considered for publication elsewhere and does not duplicate material already published. I confirm all authors consent to publication of this Work and authorise the granting of this licence.

For peer review only

Multimodal measurement approach to identify individuals with mild cognitive impairment: study protocol for a cross-sectional trial

Bernhard Grässler (corresponding author)
Institute for Sport Science, Otto von Guericke University
bernhard.graessler@ovgu.de

Fabian Herold
Research Group Neuroprotection, German Center for Neurodegenerative Diseases (DZNE), Magdeburg, Germany

Milos Dordevic
Research Group Neuroprotection, German Center for Neurodegenerative Diseases (DZNE), Magdeburg, Germany

Tariq Ali Gujar
Institute for Sport Science, Otto von Guericke University

For peer review only - http://bmjopen.bmj.com/site/about/guidelines.xhtml

Magdeburg, Germany

Sabine Darius

Occupational Medicine, Faculty of Medicine, Otto von Guericke University

Magdeburg, Germany

Irina Böckelmann

Occupational Medicine, Faculty of Medicine, Otto von Guericke University

Magdeburg, Germany

Notger G. Müller

Research Group Neuroprotection, German Center for Neurodegenerative Diseases (DZNE), Magdeburg, Germany

Department of Neurology, Medical Faculty, Otto von Guericke University

Center for Behavioral Brain Sciences, Magdeburg, Germany

Magdeburg, Germany

Anita Hökelmann

Institute for Sport Science, Otto-von-Guericke University

Magdeburg, Germany

For peer review only - http://bmjopen.bmj.com/site/about/guidelines.xhtml

Word count

5870

ABSTRACT

Introduction

The diagnosis of mild cognitive impairment (MCI), i.e., the transitory phase between normal age-related cognitive decline and dementia, remains a challenging task. It was observed that a multimodal approach (simultaneous analysis of several complementary modalities) can improve the classification accuracy. We will combine three non-invasive measurement modalities: functional near-infrared spectroscopy (fNIRS), electroencephalography (EEG) and heart rate variability (HRV) via electrocardiography (ECG). Our aim is to explore neurophysiological correlates of cognitive performance and whether our multimodal approach can aid in early identification of individuals with MCI.

Methods and analysis

This study will be a cross-sectional with MCI patients (MCI) and healthy controls (HC). The neurophysiological signals will be measured during rest and while performing cognitive tasks: (i) Stroop, (ii) N-back and (iii) verbal fluency test. Main aims of statistical analysis are to (1) determine the differences in neurophysiological responses of HC and MCI, (2) investigate relationships between measures of cognitive performance and neurophysiological responses and (3) investigate whether the

For peer review only - http://bmjopen.bmj.com/site/about/guidelines.xhtml

For peer review only

classification accuracy can be improved by using our multimodal approach. To meet these targets, statistical analysis will include machine learning approaches.

This is, to the best of our knowledge, the first study that applies simultaneously these three modalities in MCI and HC. We hypothesize that the multimodal approach improves the classification accuracy between HC and MCI as compared to a unimodal approach. If our hypothesis is verified, this study paves the way for additional research on multimodal approaches for dementia research and fosters the exploration of new biomarkers for an early detection of non-physiological age-related cognitive decline.

Ethics and dissemination

Ethics approval was obtained from the local Ethics Committee (reference: 83/19). Data will be shared with the scientific community no more than 1 year following completion of study and data assembly.

Trial registration

ClinicalTrials.gov, NCT04427436, registered on 10 June 2020, https://clinicaltrials.gov/ct2/show/study/NCT04427436.

Keywords

Multimodal measurement approach, mild cognitive impairment (MCI) detection, neurodegenerative diseases, electroencephalography (EEG), functional near-infrared spectroscopy (fNIRS), heart rate variability (HRV), electrocardiography (ECG)

For peer review only - http://bmjopen.bmj.com/site/about/guidelines.xhtml

Strengths and limitations of this study

- This study will be the first to use a multimodal measuring approach to determine neurophysiological responses of elderly with mild cognitive impairment and healthy controls.
- Differences between these two groups will be investigated with electroencephalography, functional near-infrared spectroscopy and heart rate variability at resting state and while performing cognitive tasks.
- This study will provide valuable information about certain neurophysiological parameters which are promising for an early identification of people which are at a higher risk of an overly age-related decline in cognitive performance.
- We hypothesize that this multimodal approach improves the classification accuracy between elderly with mild cognitive impairment and healthy controls as compared to a unimodal or a bimodal approach.
- Since this study is the first of its kind, it has an exploratory character with a relatively small sample size, but it should provide a basic concept for large-scale studies.

1. INTRODUCTION

Mild cognitive impairment is considered as a prodromal stage of (or transition phase to) dementia. The most common cause of dementia is Alzheimer's disease (AD)[1]. It is estimated that approximately 50 million people are currently suffering from AD worldwide[2]. AD is a neurological disease with specific neuropathological (e.g., amyloid-plaques, neurofibrillary tangles) and neurochemical features (e.g., neurotransmitter deficits). On the behavioural level, AD is characterized by deficits of higher cortical functions such as memory, decision-making, visuospatial abilities, executive functioning, and language[3]. Individuals who suffer from dementia require a high level of care and support, and thus the disease entails a substantial burden for the health care systems[4]. For instance, in Germany, the annual costs are estimated at 42.6 billion Euros[5] whereas the global costs are considered to have

For peer review only

For peer review only - http://bmjopen.bmj.com/site/about/guidelines.xhtml

crossed the $1 trillion threshold in 2018[6]. Given that the number of individuals with dementia will increase further as a consequence of the demographic change, these annual health care expenditures are expected to grow, too.

In contrast to patients with dementia, patients with mild cognitive impairment (MCI) have only marginal limitations in activities of daily living, but show some deteriorations in specific cognitive domains[7]. A person is considered to suffer from MCI if the following criteria are fulfilled: memory complaint, normal activities of daily functioning, normal general cognitive function, abnormal memory for age, and not demented[8]. There are four types of MCI: amnestic MCI single domain, amnestic MCI multiple domain, non-amnestic MCI single domain and non-amnestic MCI multiple domain[9]. MCI patients with the amnestic subtype are at increased risk for later dementia of the Alzheimer's type compared to people with normal cognition[10]. As these mild cognitive impairments are often not evident on a behavioural level, individuals with MCI are difficult to identify. During the stage of MCI, reduction of lifestyle-related risk factors, such as physical inactivity, may slow down or postpone the progression of this neurological syndrome[11-13]. So far, no drug has been approved for the symptomatic or causal treatment of MCI[14]. Nevertheless, an early diagnosis of MCI is indispensable for a better understanding of the neurobiological mechanisms mediating the transition of the inexorable age-related cognitive decline to dementia. This, in turn, may allow for a timely initiation of preventive strategies slowing, postponing or, at best, counteracting the transition to a serious manifestation of dementia.

The diagnosis of MCI based on clinical features alone is often challenging and relatively unreliable. Therefore, the identification of biomarkers that aid an early identification of neurodegenerative processes has become a focus of current research[15]. Neuroimaging methods such as magnetic resonance imaging (MRI) or positron emission tomography (PET) are popular tools in the investigation of neurodegenerative diseases. However, these methods have several disadvantages. They are expensive, have limited eligibility and the participant is not allowed to move. Additionally, the use of radioisotopes in PET results in exposure to ionizing radiation which involves detrimental health

For peer review only - http://bmjopen.bmj.com/site/about/guidelines.xhtml

risks[16-17]. Biomarkers in the cerebrospinal fluid (CSF), such as Aβ42, constitute another approach for identifying AD pathology[1]. However, drawing CSF is stressful for the patient, can entail complications and does not necessarily yield unequivocal results. Thus, it is necessary to investigate other measurement methods able to detect a pathological cognitive decline timely while avoiding the above-mentioned limitations.

Functional near-infrared spectroscopy (fNIRS) is an optical neuroimaging technique that is based on the theory of neurovascular coupling and optical spectroscopy[18]. fNIRS allows the non-invasive investigation of cortical hemodynamic changes associated with brain activity[19]. In this regard, it is assumed that neuronal activity enhances the regional cerebral blood flow to the neuronal tissue at work which, in turn, leads to a local increase in the concentration of oxygenated haemoglobin (oxyHb) and decrease of deoxygenated haemoglobin (deoxyHb)[18]. Since oxyHb and deoxyHb have different light absorption spectra, the relative activity-dependent concentration changes of oxyHb and deoxyHb can be determined and allow us to make conclusions about cortical brain activity[17]. More details on physiological and methodological background of fNIRS can be found in the referenced literature[18, 20-21].

The advantages of fNIRS are its portability, tolerance to motion artefacts (compared to EEG and MRI), higher temporal resolution (compared to MRI), low costs (compared to MRI and PET), it is easy to use and more participants are eligible than for MRI (e.g., no restriction due to claustrophobia or metallic implants). The disadvantages are its lower spatial resolution (compared to MRI), low penetration depth, lack of standardization in data analysis and, due to the delay in the hemodynamic response, there is a time delay in the signal curve, too[17].

fNIRS has become a frequently used technique for the investigation of neurological diseases such as AD[23-25]. There is evidence in the literature showing that fNIRS offers a great potential to become a valuable tool to identify individuals with a higher risk of developing dementia as changes in cortical hemodynamic obtained during a standardized cognitive test allow to discern between healthy and

For peer review only - http://bmjopen.bmj.com/site/about/guidelines.xhtml

diseased individuals[26-33]. Young participants showed a stronger activation in the left hemisphere in the more difficult task conditions. Elderly participants showed no lateralization and a decreased activation in the difficult task condition. This phenomenon is called “Hemispheric Asymmetric Reduction in OLD adults” (HAROLD) model[34].

Electroencephalogram (EEG) is another technique for quantifying neurophysiological processes. EEG measures electrical activity provoked by the firing of cortical neurons within the brain. EEG is an optimal tool for recording the complex dynamic neural activity due to the excellent temporal resolution in milliseconds and reasonable spatial resolution[35]. The potential to detect pre-dementia AD/MCI condition has already been shown[36-39].

In addition to the most commonly used method of frequency analysis, it is also recommended to apply other more sophisticated EEG methods, like brain mapping, connectivity analysis or the analysis of event-related potentials, which can lead to a further specification of the diagnosis of dementia[40-44].

The specific neural activity in response to a certain stimulus can be measured by event-related potentials (ERPs). The most common component we can find in response to sensory, cognitive, or motor stimuli are P100, P200, P300, P600, N100 and N400, which are used in cognitive science (see for review, [45]). Importantly, it is not sufficient to study the cortical modulation in isolation. Cortical functions are influenced by functional integration. EEG is an optimal technique to understand the communication between different areas of the brain by functional and effective connectivity analysis. Functional connectivity is defined as the correlation between different areas of the brain. Effective connectivity is defined as direct or indirect influence that one neural system exerts on the other neural system. The connectivity can be investigated by sources and EEG channels[46-48]. Beside connectivity, it is also possible to construct the brain map from the recording of electrical potentials with electrode distribution over the skull. The brain mapping can be helpful to identify activated structures of the cortex during task execution[49-50].

For peer review only - http://bmjopen.bmj.com/site/about/guidelines.xhtml

Electrocardiography (ECG) will be the third assessment method. In particular, we will determine the heart rate variability (HRV) from the ECG. The HRV describes the beat-to-beat variation of subsequent NN-intervals. These fluctuations in the heart rate are regarded as an indicator of the functional state of the autonomic nervous system (ANS) and is related to psychophysiological aspects such as self-regulation on a cognitive, emotional, social, and health level[51-52]. HRV is the result of the complex interaction between sympathetic and parasympathetic influences[53-54]. A relatively low HRV indicates an increased sympathetic state, an abnormal regulation and inadequate adaptation of the cardiovascular system, and is a sign of depletion or pathology. Hence, HRV is a promising marker to diagnose pathological states[54] and an optimal HRV is critical to health and well-being[55].

HRV measurements provide the advantages that they are easy to use, not expensive, portable, non-invasive and the recordings are relatively easy to interpret. However, on the downside HRV values are strongly influenced by individual differences, there is a relatively large number of influencing factors that have to be controlled for, and there is still no generally accepted consensus concerning data acquisition and data processing[51, 56-57].

Measures of HRV have been used to index vagal activity[58]. Root mean square of successive differences between adjacent normal NN intervals (RMSSD), percentage of successive normal NN intervals differing more than 50 ms (pNN50) and power in high (0.15–0.40 Hz) frequencies (HF) are supposed to reflect vagally mediated HRV[51, 55, 59]. According to the neurovisceral integration model, neural structures responsible for affective, cognitive and physiological regulation are associated with vagally mediated cardiac function[60]. Especially the prefrontal cortex is associated with HRV measures as it is connected with the amygdala and cardiovascular system[61]. Vagally mediated HRV is supposed to be linked with “a set of neural structures that have been implicated in cognitive, especially executive function”[60]. A positive connection between resting HRV and cognitive functioning has already been demonstrated[62-66]. In a recent meta-analysis, the effect size (ES) magnitude of HRV measures in the evaluation of autonomic dysfunction in older people with dementia was investigated[67]. Although the small ES does not support the use of HRV as a single biomarker to

For peer review only - http://bmjopen.bmj.com/site/about/guidelines.xhtml

diagnose dementia, the results suggest autonomic dysfunction in dementia. Since biological processes such as ANS activity are complex and non-linear, several authors suggest non-linear HRV measures[68-72]. In this context, Sample Entropy recorded at rest, was related to a better cognitive performance, but traditional time or frequency indices were not[71]. The sensitivity and reliability of non-linear measures such as Poincaré and detrended fluctuation analysis (DFA) for mental effort tasks could be proved in healthy seniors[69]. In a recent study, D2, the RRI dimension correlation, could be better related to the mental workload than time or frequency indices[68].

Cognition relies on complex neurophysiological processes. In particular, the solving of a cognitive task is associated with task-related changes in cerebral hemodynamics, cerebral electrical activity and changes in the ANS. These task-related neurophysiological changes can be assessed by fNIRS, EEG and HRV[73] separately (unimodal approach) or simultaneously (multimodal approach). A clear advantage of the multimodal assessment approach of task-related neurophysiological changes (e.g., cognition-related brain activity) is its ability to reduce and/or to compensate for inherent limitations of a single measurement modality (e.g., artefacts that are reflected in only one modality)[74]. As single tests, they might be not accurate enough for differentiating healthy controls from cognitive impaired participants. EEG, fNIRS, and HRV provide complementary information about different neurophysiological systems which, in turn, foster an improvement of the classification accuracy. Indeed, cognitive neuroscience research now focuses on the simultaneous acquisition by non-invasive modalities to improve their performance and information content[16]. Combined fNIRS-EEG[75], EEG-HRV[76-77] and fNIRS-HRV[78] measurements were already applied in different fields of brain research. It was observed that classification accuracy can be improved when using different measurement modalities simultaneously[79-86]. However, so far and to the best of our knowledge, there is no published study available which uses the above-described multimodal approach to investigate the neurophysiological cognition-related differences between healthy older individuals and older individuals with MCI.

The primary aim of this study is to investigate the difference between healthy controls and cognitively impaired participants in neurophysiological signals. We will use fNIRS, EEG and ECG since they are non-

For peer review only - http://bmjopen.bmj.com/site/about/guidelines.xhtml

invasive and provide some advantages compared to MRI, PET and measures of cerebrospinal fluid. As the most of the currently available studies used, to the best of our knowledge, only two different measurement modalities (mostly EEG and fNIRS), we hypothesize that our approach improves classification accuracy considerably. The second aim of this study is to investigate possible neurobehavioral relationships between measures of cognitive performance and measures of fNIRS, EEG, and HRV. The third aim of this study is to systematically compare the classification accuracy of uni-, bi- and multimodal measurement approaches. By saying that, we wish to emphasize that this study has an explorative character aiming to identify cognition-related neurophysiological parameters that are promising for MCI detection. This study should provide a basic concept for further studies using a multimodal measurement approach and promote research for a better understanding of the neurobiological mechanisms leading to dementia.

2. METHODS AND ANALYSIS

2.1. Study design

This cross-sectional study will be conducted by a multidisciplinary team of researchers from Department of Sport Science at the Otto von Guericke University Magdeburg, the Medical Faculty of the Otto von Guericke University Magdeburg and the German Center for Neurodegenerative Diseases. The study protocol was approved by the Ethics Committee of the Otto von Guericke University Magdeburg (reference: 83/19) and is in accordance with the Declaration of Helsinki. This study was registered in ClinicalTrials.gov (NCT04427436) on the 10th June 2020.

For peer review only - http://bmjopen.bmj.com/site/about/guidelines.xhtml

2.2. Participants

Sample size is based on a previous fNIRS-study using the N-back task[30] and was calculated a priori by using G*Power 3.1[87]. Based on their reported t- and p-values with respect to the means of oxyHb of both groups during the cognitive task, a sample size of 15 participants in each group will be needed to achieve a statistical power of 80 %. Assuming an attrition rate of 20 %, the estimated sample size is at least 36 participants, 18 per group. To consider drop-outs due to technical problems, we plan to measure at least 20 participants per group.

This study will involve MCI patients who have been diagnosed by an experienced neurologist, based on standardized clinical, neuropsychological and imaging criteria[88]. Healthy participants will be recruited by advertisements in local newspapers. Interested individuals will be informed about the aim of the study and first be screened by telephone to check for general eligibility according to our criteria. The two groups will be age, gender-, handedness- and education-matched. At the beginning of the study, each participant will provide a personally signed and dated informed consent document indicating that the individual has been informed of all pertinent aspects of the study. Participants must be native German-speaking adults who are between 55 and 80 years old. All participants will be financially rewarded to compensate for their participation.

Exclusion criteria are:

- other neurological diseases (i.e., epilepsy, multiple sclerosis)
- severe cardiac diseases (i.e., severe cardiac insufficiency, cardiac pacemaker, valvular defect)
- mental diseases (i.e., schizophrenia, depression)
- orthopaedic diseases (i.e., bone fracture in last six months, symptomatic slipped disc)
- muscular diseases (i.e., myositis, tendovaginitis)
- severe endocrinologic diseases (i.e., manifest hypothyroidism or hyperthyroidism, adiposity [BMI >30], juvenile-onset diabetes)
- injury or surgery in last six months

For peer review only

For peer review only - http://bmjopen.bmj.com/site/about/guidelines.xhtml

- use of illegal intoxicants or alcohol abuse
- uncorrected poor eyesight or hearing
- colour blindness / red-green weakness
- pregnancy or breastfeeding
- using one of the following medications: neuroleptics, narcotic analgesics, benzodiazepines and psychoactive medications

At the beginning of the study, all participants will be screened by using the CERAD test battery[89]. This cognitive test battery evaluates the performance in semantic verbal fluency, word retrieval, constructional praxis, visual memory, verbal memory, global cognition (Mini Mental State Examination [MMSE]) and motor speed (Trail Making Test A). The CERAD has been shown to be a valid and reliable assessment tool to identify individuals with cognitive impairments[90].

For peer review only

2.3. Assessments

Participants will attend three visits within one week and undergo the following assessments:

1. clinical assessment: CERAD and medical examination, taking blood sample [i.a. BDNF (brain-derived neurotrophic factor)]
2. sociodemographic assessment: questionnaires
3. neurophysiological and neuropsychological assessment: EEG/fNIRS/ECG simultaneously at resting state and during cognitive tasks

2.4. Neurophysiological and neuropsychological assessment

Research staff collecting data are blinded concerning the cognitive status (MCI or HC) of the participant to avoid bias. EEG, fNIRS and ECG will be recorded simultaneously at resting state for ten minutes. The

For peer review only - http://bmjopen.bmj.com/site/about/guidelines.xhtml

measurement standards for the resting state measurement follows the recommendations of Laborde et al.[51]. Accordingly, the participants are asked to sit on a comfortable chair with their knees bent at a 90° angle and their hands on their thighs. Furthermore, they are advised to relax, breath normally and move as little as possible. As recommended in the literature, the participants will rest in the above-described position for at least five minutes before the baseline recordings are obtained[51]. Subsequently, the participants complete three cognitive tasks while their neurophysiological signals are measured. The cognitive tasks are Stroop, N-back and a verbal fluency test (VFT). This procedure is comparable to the study of Yang et al.[31] but in addition to this study (i) we will conduct the assessment of EEG and ECG and (ii) we will use modified versions of the cognitive tasks. Afterwards, a second resting-state measurement will be conducted (recovery phase)[51].

According to the multistage concept in psychophysiology of Fahrenberg, objective data of the cognitive performance, objective physiological data and subjective data of the personal feeling will be collected[91]. The latter will be recorded by the NASA-TLX questionnaire. The NASA-TLX provides an overall workload score with six dimensions: mental demands, physical demands, temporal demands, own performance, effort, and frustration[92]. The overall workload is the weighted average of these dimensions. After the cognitive tests, participants will be instructed to rate each dimension on a visual scale from 1 to 20 points. It is an easy to use tool and its results can be compared with participants` performance and their neurophysiological responses. The NASA-TLX is a widely used tool with a high reliability and validity[93].

2.4.1. Cognitive tasks

During the whole assessment, the participants are sitting in front of a computer screen on a comfortable chair and are asked to avoid head movements as much as possible. Before the experiment, the participants will be briefed on the task instructions and experimental design. The instructions are presented on a printed paper and explained verbally by the investigator. Each task

For peer review only

block begins with a 5 s instruction cue that informs the participant about the task condition. All tasks will be administered via computer which allows to measure reaction times and responses of the participants exactly. To synchronize all signals, temporal triggers are delivered simultaneously to EEG and fNIRS systems via the software Presentation (Neurobehavioral Systems Inc, USA).

2.4.1.1. Stroop

A computerized version of the Stroop test will be applied[94]. This widely used test requires the executive functions inhibition and cognitive control. These are crucial for completion of complex cognitive tasks and everyday activities[95]. The classic Stroop test elicits a conflict situation since the meaning of a colour-word and the ink colour do not match. Behavioural responses to these incongruent stimuli are usually slower and less accurate than responses to congruent stimuli. The behavioural difference between incongruent and congruent stimuli is called the Stroop effect, which has been used as an index of cognitive control[96]. Since the prefrontal cortex plays a predominant role in cognitive control]97] and have shown to be affected by MCI[25], fNIRS optodes will be placed on the prefrontal cortex.

Our Stroop test includes three experimental conditions (pure congruent, pure incongruent and mixed congruent and incongruent stimuli) with three blocks in each condition (see Figure 1). Prior to each block, a baseline measurement having nearly the same length as the task blocks will be applied in order to assess a baseline for the hemodynamic activity. During the baseline measurement, participants are requested to sit still and relax. According to recent recommendations[18], the duration of the baseline measurement should not be a multiple of 10 s to avoid the overlap with the Mayer waves. In each block, colour-words are consecutively presented in the middle of the computer screen. A black background was chosen to avoid overstraining the eyes. Each colour has a corresponding button. Four different colour-words will appear: “RED”, “GREEN”, “BLUE” or “YELLOW” in German language. In the congruent condition, the meaning of the colour-word and the ink colour match. In the incongruent condition, the colour-word is printed in an incongruent ink colour, e.g., the word "RED" is presented

For peer review only - http://bmjopen.bmj.com/site/about/guidelines.xhtml

in blue colour. Participants are instructed to identify the colour of the word by pressing the appropriate button and not reacting on the meaning of the word. In this example, the participant must press the button for the ink colour (blue). Participants should react as fast and as correct as possible. In the third block, congruent and incongruent stimuli are presented. Participants are instructed to react on the colour of the word. Although the typical clinical version of the Stroop tests include a pure block of neutral (congruent) stimuli and a pure block of incongruent stimuli, some authors criticise this pure-block design since it has some limitations[95]. Instead, a mixed block design with congruent stimuli within an incongruent condition challenge goal-maintenance capacities because the congruent stimuli promote the inappropriate but more automatically response of reading the word and produce larger Stroop effects than pure incongruent blocks[95]. This mixed design was already used in some studies[98-102]. Hence, we will use a pure incongruent and a mixed block. The mixed block consists of 50 % congruent and 50 % incongruent stimuli. The participants should react in both variants to the colour of the word. Prior to task, the participants will be adequately familiarized with the test by performing a sufficient number of practice trials.

For peer review only

For peer review only - http://bmjopen.bmj.com/site/about/guidelines.xhtml

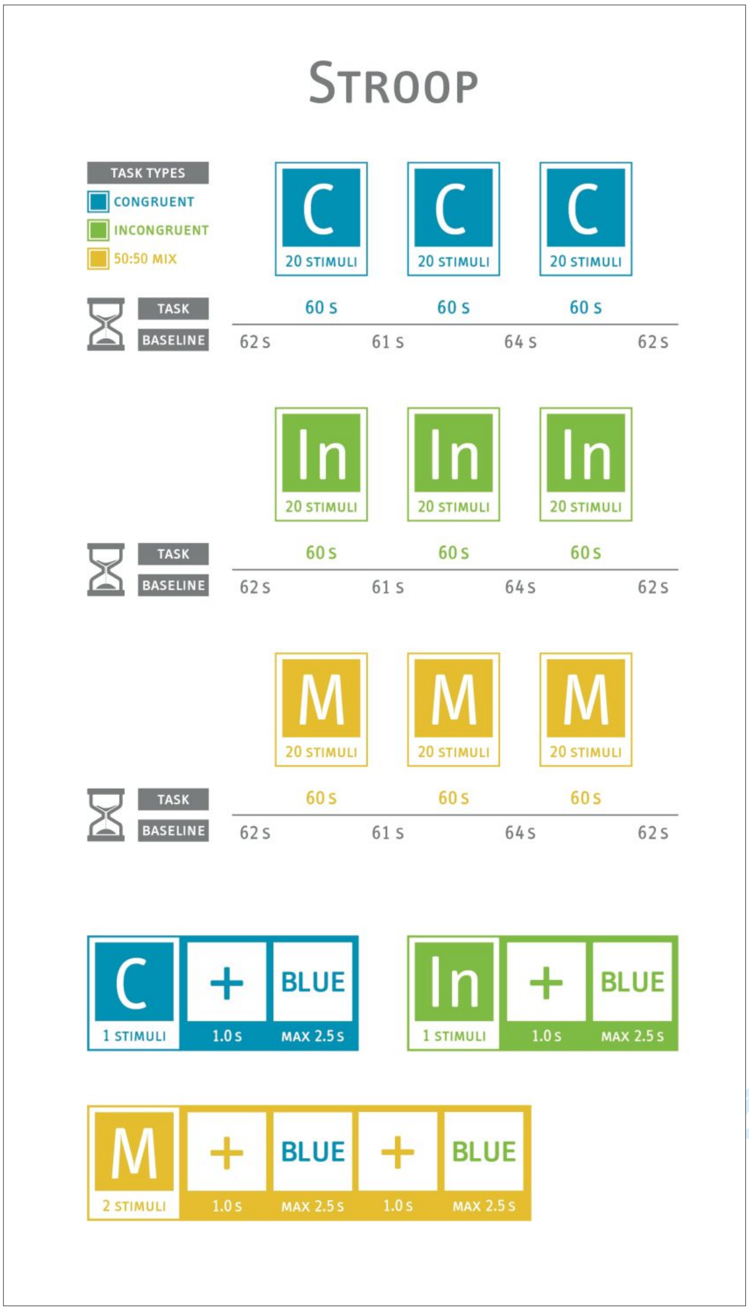

Figure 1: Paradigm of the Stroop test.

2.4.1.2. N-back

The N-back was first introduced by Kirchner[103] and is a frequently used task to measure working memory capacity. In our study, three conditions (0-, 1- und 2-back) will be used (see Figure 2). Single-digit numbers with a presentation time of 1500 ms are presented consecutively in three blocks in the

For peer review only - http://bmjopen.bmj.com/site/about/guidelines.xhtml

middle of the screen. The interstimulus interval is 500 ms. As in the Stroop test, a baseline measurement will be included prior to each task block and after the last block.

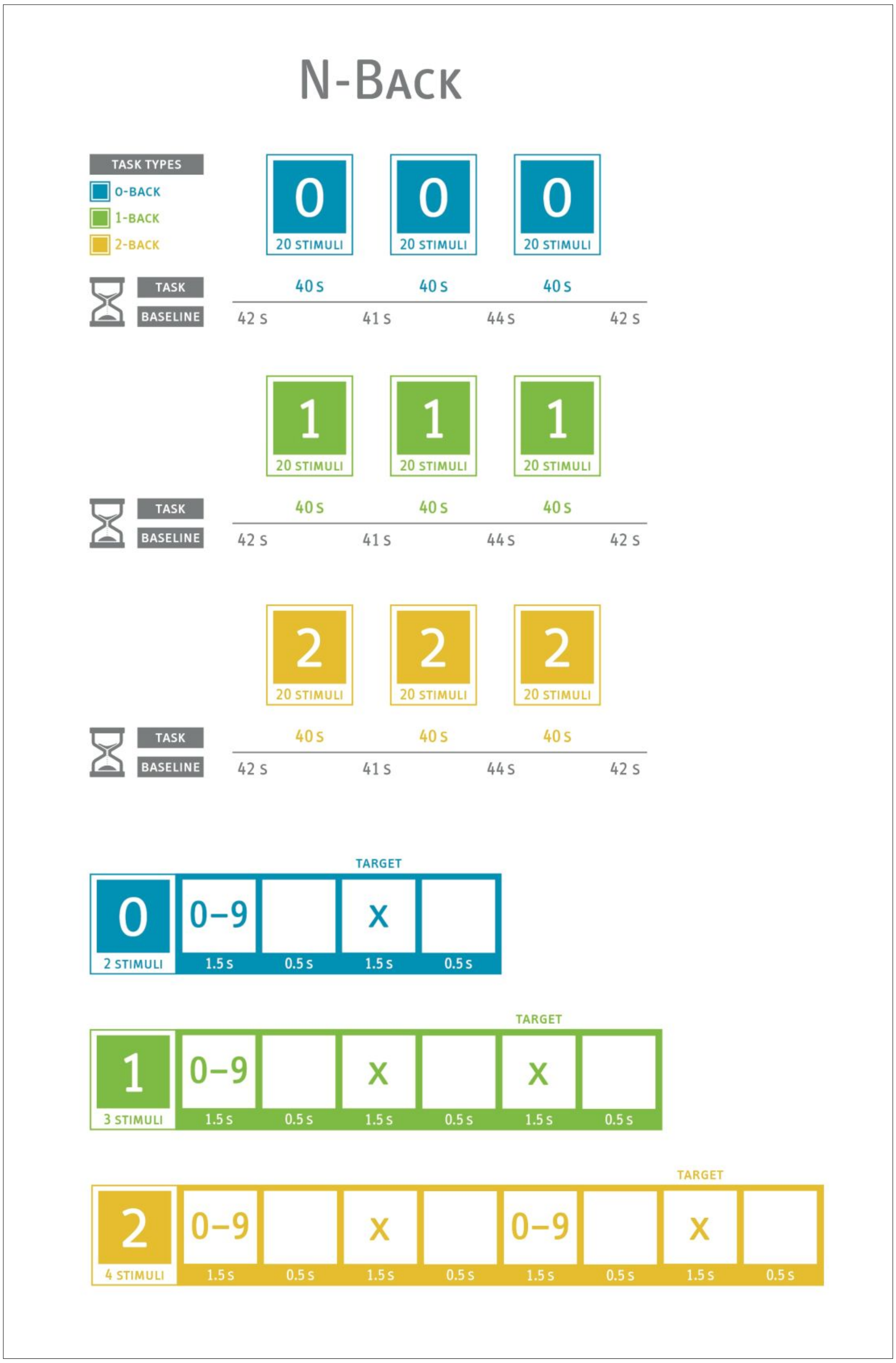

Figure 2: Paradigm of the N-back test.

In the 0-back condition, the participants are asked to press the target button only when the number "7" appears. All other numbers must be ignored. In the 1-back condition, the participants are asked to press the target button only when two identical numbers appear in succession. In the 2-back condition, the participants are asked to press the target button only when the current number matches the second-last number displayed before. In all three conditions, 25 % of stimuli are targets. As in the Stroop test, participants will be instructed to react as fast and as correct as possible. Prior to task, the participants will be adequately familiarized with the test by completing a sufficient number of practice trials.

2.4.1.3. Verbal fluency test

Verbal fluency is a cognitive domain that worsens with the development of Alzheimer's disease[104]. Therefore, we plan to use a verbal fluency test (VFT) which is based on the "Regensburger Wortflüssigkeitstest"[105]. This test is frequently used in fNIRS studies investigating neurological diseases[26, 32, 104, 106]. Based on these studies, our test consists of two conditions. Both conditions will be presented three times in a row for 30 s with a resting block for 31–34 s between each block (see Figure 3). During the resting blocks, the participants are requested to sit still and avoid speaking. We use a phonological (letter) and a semantic (category) condition. The instructions will be displayed on a screen to avoid instruction bias. Participants are requested to avoid movements during this test.

In the phonological condition, the participants are instructed to pronounce as many German words (nouns, verbs, adjectives) as possible beginning with a specific letter (“A”, “F”, “P”) without using proper names, numbers, repetitions or words in different forms or with different endings. In the semantic condition, participants are instructed to pronounce as many words as possible belonging to a specific category (“forenames”, “fruits”, “flowers”).

For peer review only - http://bmjopen.bmj.com/site/about/guidelines.xhtml

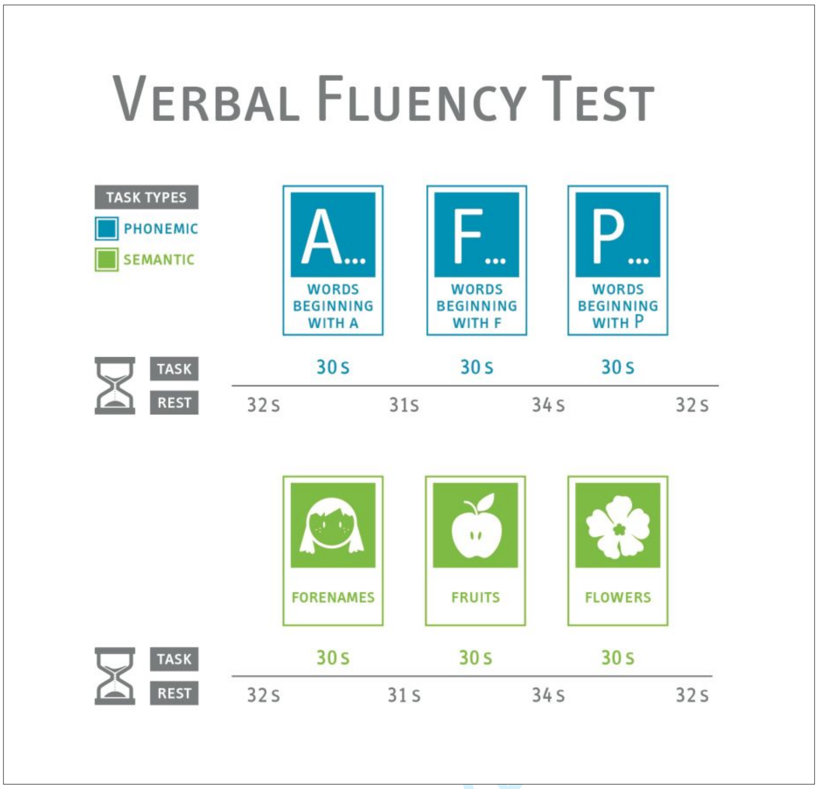

Figure 3: Paradigm of the verbal fluency test.

2.4.2. Neurophysiological assessment

2.4.2.1. fNIRS

In this study, a portable continuous wave fNIRS system (NIRSport™, NIRx Medical Technologies, Glen Head, New York, USA) will be used to record cortical hemodynamics with a frequency of 10.2 Hz. The fNIRS-system consists of the following: eight light sources which emit light at wavelengths of 760 and 850 nm, eight light detectors, and a short-distance detector bundle (NIRx Medical Technologies, Glen Head, New York, USA) which allows to quantify changes in extracerebral layer (i.e., blood flow in the scalp). As shown in Figure 4, the fNIRS optodes will be positioned according to the 10–20 EEG system[107] by using a standardized cap (EasyCap GmbH, Herrsching, Germany). We will perform a virtual and probabilistic spatial registration using the software fOLD (fNIRS Optodes' Location Decider)[108] and the Broadmann atlas (BA)[109] to assign the fNIRS measurement channels with long source-detector separation to specific brain regions (see Additional file 1 for detailed overview).

For peer review only - http://bmjopen.bmj.com/site/about/guidelines.xhtml

The data processing of fNIRS follows recent recommendations[18, 22, 110] and will be conducted using the latest version of the "Homer" software package[111]. In brief, we will conduct the following processing steps: (i) exclude noisy channels by using enPruneChannels function, (ii) convert raw light intensity changes into changes in optical density by using hmrIntensity2OD function, (iii) perform motion artefact correction by using sophisticated filter methods (e.g., wavlet filter - hmrMotionCorrectWavelet filtering function)[112], (iv) perform a correction for physiological artefacts such as heart beat and instrumental noise by using hmrBandpassFilt function, (iv) convert optical density data of both wavelengths via the modified Beer–Lambert law (MBLL) into concentration changes of oxyHb and deoxyHb by using the hmrOD2Conc function and an individually calculated differential path length factor[113], (v) correct for extracerebral blood flow by using hmrDeconvHRRF_DriftSS function[114-115], and (vi) perform a baseline correction and calculation of block averages for oxyHb and deoxyHb changes over all trials and for each measurement channel by using hmrBlockAvg function. In the final step, the pre-processed time-series of oxyHb and deoxyHb are exported and the cognition-related changes in these two chromophores are used for further statistical analysis.

For peer review only

For peer review only - http://bmjopen.bmj.com/site/about/guidelines.xhtml

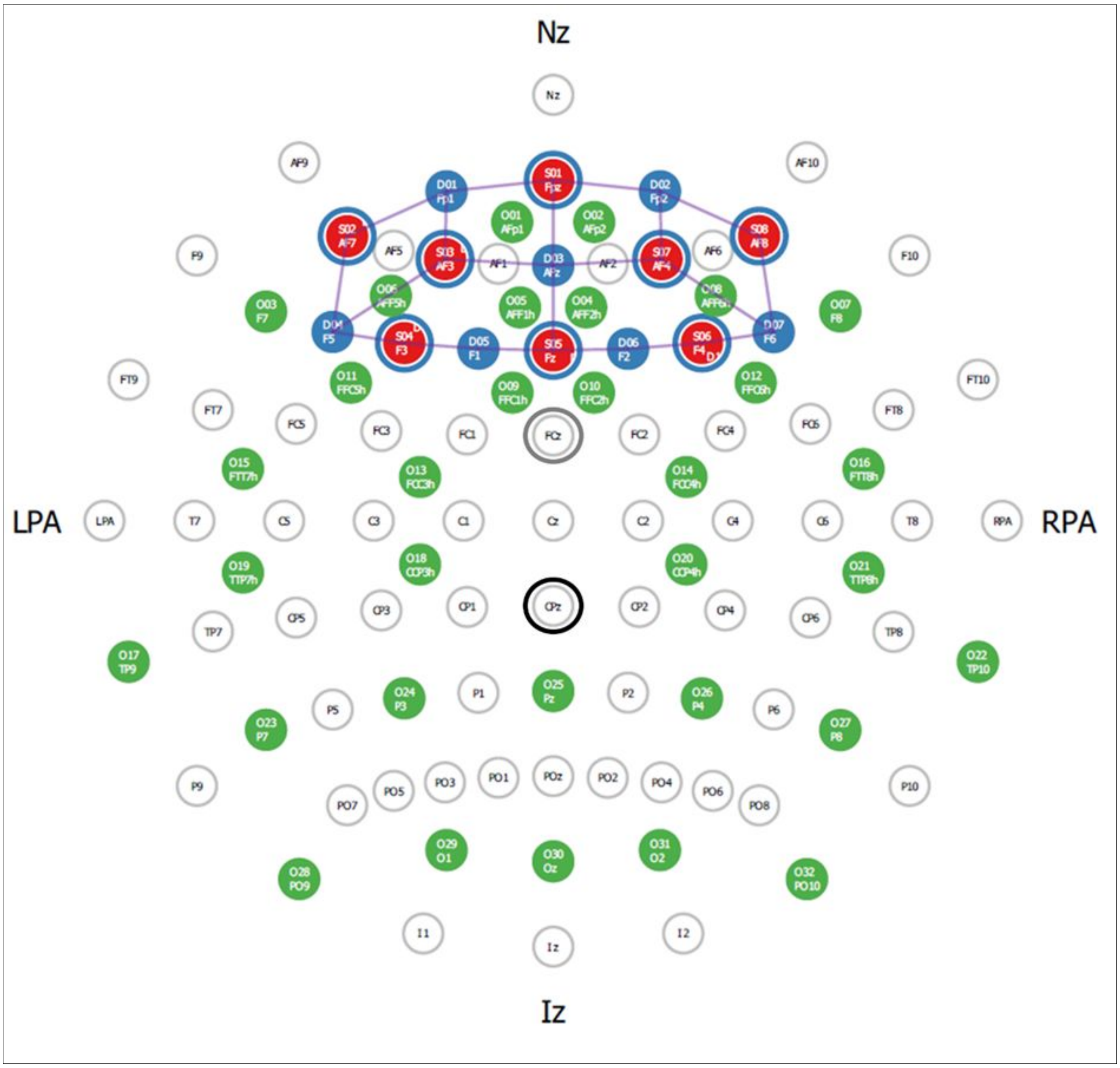

Figure 4: schematic illustration of the fNIRS optode positions and EEG electrode positions. The 'red dots' represent sources whereas the 'blue dots' represent detectors. A 'blue circle' around a detector indicates the position of a short-separation channel. The measurements channels, which are displayed in 'purple', are combinations of distinct sources and detectors. The 'green dots' illustrate the position of the EEG electrode. The reference electrode and ground electrode are indicated by a 'grey circle' and 'black circle' respectively.

2.4.2.2. EEG

Electroencephalography data will be acquired by the Brain Vision wireless MOVE system (Brain Products GmbH, Munich, Germany) along with Brain Vision Recorder Version 1.21.0102 (Brain Products GmbH, Munich, Germany). The system will be triggered by the computer running the Presentation software. The EEG/fNIRS cap will be customized according to the 10–20 system with the

For peer review only - http://bmjopen.bmj.com/site/about/guidelines.xhtml

provision of fNIRS optodes and 34 EEG channel slim active electrodes [AFp1, AFp2, AFF5h, AFF1h, AFF2h, AFF6h, F7 , F8, FFC5h, FFC1h, FFC2h, FFC6h, FTT7h, FCC3h, FCC4h, FTT8h, TTP7h, CCP3h, CCP4h, TTP8h, TP9, TP10, P7, P3, Pz, P4, P8, Po9, O1, Oz, PO10, Fz (reference) and ground][82]. The electrode location will be customized in the Brain Vision Recorder workspace according to the EEG elastic cap channel position in consideration of Theta and Phi values[116]. The impedance will be kept below 5 KΩ before EEG data recording. The cognitive tasks will be recorded following a baseline measurement of ten minutes. The EEG data will be recorded at 1.000 Hz. The outcome of the results will be focused on time and frequency domain of ERPs, power spectrum analysis, source localization and connectivity.

The EEG recorded data will be analysed by the Brain Vision Analyzer 2.2.0 (Brain Products GmbH, Munich, Germany). The EEG post-processing will be divided into two blocks whereas P100, P200, P300, P600, N100 and N400 ERPs[2], PSD (power spectral density; frequency analysis), connectivity and source localisation will be done for the N-back and Stroop, and PSD will be done for the VFT. The pre-recorded EEG data will be resampled at 256 Hz and will be re-referenced to mean mastoids (MM)[117]. The referenced data will be filtered for ERPs at high pass 0.1 Hz and low pass at 40 Hz with 50 Hz notch filter. For PSD, connectivity and source localisation a low pass filter of 85 Hz will be used. The EEG data processing pipeline after re-referencing and filtering is shown in Figure 5.

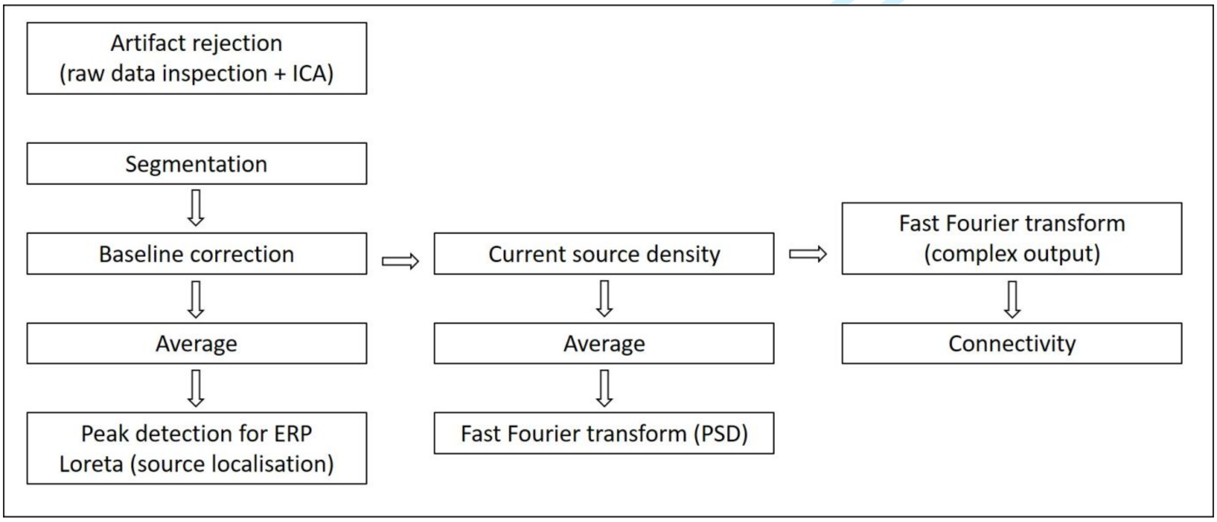

Figure 5: EEG data post-processing pipeline after re-referencing and filtering.

For peer review only - http://bmjopen.bmj.com/site/about/guidelines.xhtml

2.4.2.3. ECG

We will record the ECG of the participants during the experimental session with an ECG medilog AR12 plus (Schiller, Baar, Switzerland). This device consists of three channels with a sampling rate of 1.000 Hz. By using this device, we can record the NN intervals in millisecond range which enables us to accurately calculate the HRV and to precisely identify cardiac arrhythmia. The raw ECG data will be uploaded to the Medilog Darwin Analysis Software package and analysed using the Kubios premium 3.3 software package (University of Kuopio, Finland). The HRV analysis will focus on the following parameters: root mean square of successive differences adjacent normal NN intervals (RMSSD), percentage of successive normal sinus NN intervals differing more than 50 ms (pNN50) and power in high (0.15–0.4 Hz) frequencies (HF). Further time and frequency domain parameters[118-119] will be analysed as well. In addition, we will also consider non-linear HRV parameters to study the non-linear dynamic properties that influence heart rate[68]. Due to the relatively long latency of the cardiac system[55], the HRV (as the fNIRS parameters) will be averaged over each block of the three cognitive tasks. Following the recommendations of Laborde et al.[51], we plan to record and analyse the HRV at three timepoints:

- at resting state before the cognitive tasks,
- during the cognitive tasks for each block and
- after the cognitive tasks at resting state in the recovery phase.

The baseline measurement before the cognitive tasks will serve as assessment of the vagal tone of the participants. The assessment of the vagal tone is important because “vagally mediated HRV may serve to index the functional capacity of a set of brain structures that support the effective and efficient performance of cognitive executive-function tasks including working memory and inhibitory control”[60]. The “phasic HRV” will be evaluated through comparing the HRV at rest with the values recorded during the cognitive tasks[51]. It shows how the ANS reacts and how the participant adapts

For peer review only

For peer review only - http://bmjopen.bmj.com/site/about/guidelines.xhtml

to cognitive demands. Finally, the HRV will be recorded after the cognitive tasks at resting state (recovery phase). This experimental design allows for investigation of tonic HRV for each of the three measurement points (baseline, during and after the cognitive tasks). Furthermore, we can assess the phasic HRV because we will be able to detect the change between baseline and event ("reactivity"), the change between task and post-event ("recovery") and the change between baseline and post-event[51].

2.5. Statistical analysis

The current study is designed to investigate biomarkers that can identify participants with an increased risk for cognitive deterioration. Thereto, several biomarkers will be evaluated, as possible candidates, for an early identification of MCI. We are interested in the transition state between healthy aging and MCI. Consequently, our statistical analysis will include:

- analysis of the differences between HC and MCI with respect to cognitive performance and physiological parameters,
- correlational analysis between cognitive performance and physiological parameters and
- machine-learning based logistic regression approach for classification into HC and MCI.

To investigate effects of group, t test for normal distributed variables will be used, in other cases the Mann Whitney U test. Correction for multiple comparisons will be taken into account with False Discovery Rate[120]. To investigate the correlations, we will use Pearson or Spearman correlation analysis. To control for effects of age, gender and education as confounders, partial correlation will be applied. Table 1 shows the measured physiological parameters of all three cognitive tasks. Concerning ECG analysis, we will also measure the HRV in resting state before (baseline) and after the cognitive tasks (recovery).

For peer review only

The main aim of our study is to develop EEG/fNIRS/HRV measures that discriminate among HC and MCI and show that this multimodal measuring approach is promising and accurate for identification of MCI. For that purpose, we will use and adapt the machine-learning algorithm already used for bimodal approaches[79-81, 83-84, 86]. In a first step, after pre-processing and artefact correction, parameters from the three modalities will be extracted and the most discriminating ones playing a role in the development of neurodegenerative diseases will be selected. Principal Component Analysis (PCA) can help to reduce parameters to a manageable amount by removing components with the highest variance[121]. Following parameter extraction, multimodal combining algorithm will be used for classification. To classify the signals, support vector machine (SVM) will be applied. SVM is one of the most commonly used supervised classifiers in the field of pattern recognition and has been widely adopted in many brain signal studies[74, 79-81, 83, 122]. SVM can define two or more classes by constructing an optimal hyperplane maximizing the margin of separation between the closest data points belonging to different classes. SVM can be used in linear as well as in non-linear classification scenarios based on the kernel trick[123].

Another aim of the study is to compare the performance of uni-, bi- and multimodal systems. For that reason, classification should be performed separately using different kinds of parameter sets for comparison: EEG-only, fNIRS-only, HRV-only, bimodal (EEG + fNIRS, EEG + HRV, fNIRS + HRV) and a multimodal parameter set (EEG + fNIRS + HRV).

Table 1: Physiological parameters of each device in all three cognitive tasks.

Cognitive task	Physiological measurement device	Main parameters
Stroop (congruent and incongruent)	ECG	RMSSD, pNN50, HF (absolute and nu), non-linear parameters (SD1, SD2, ApEn, SampEn, DFA, D2, RPA, MSE)

For peer review only

For peer review only - http://bmjopen.bmj.com/site/about/guidelines.xhtml

	fNIRS	oxyHb, deoxyHb, totHb
	EEG	ERPs, PSD, connectivity and source localisation
N-back (0-, 1- and 2-back)	ECG	RMSSD, pNN50, HF (absolute and nu), non-linear parameters (SD1, SD2, ApEn, SampEn, DFA, D2, RPA, MSE)
	fNIRS	oxyHb, deoxyHb, totHb
	EEG	ERPs, PSD, connectivity and source localisation
VFT (semantic and category)	ECG	RMSSD, pNN50, HF (absolute and nu), non-linear parameters (SD1, SD2, ApEn, SampEn, DFA, D2, RPAn, MSE)
	fNIRS	oxyHb, deoxyHb, totHb
	EEG	PSD

RMSSD: root mean square of the successive differences between adjacent normal RR intervals; pNN50: NN50 divided by the total number of NN intervals; HF: high frequency power in absolute and normalized units (nu) [0.15–0.4 Hz]; SD1: in Poincaré plot, the standard deviation perpendicular to the line-of-identity; SD2: in Poincaré plot, the standard deviation along the line-of-identity; ApEn: approximate entropy; SampEn: sample entropy; DFA: detrended fluctuation analysis; D2: correlation dimension; RPAn: recurrence plot analysis; MSE: mutliscale entropy; oxyHb: oxygenated haemoglobin; deoxyHb: deoxygenated haemoglobin; totoHb: total haemoglobin; ERPs: event related potentials; PSD: power spectral density.

2.6. Data management and safety

All participants will be assigned a code independently from their group allocation ensuring their anonymity. In order to guarantee the security of all data, the personal information and the data of this study will be collected and handled exclusively by the involved researchers. All data and participants` information will be handled according to the institutional data management policy of the University of Magdeburg. The original documents will be kept by the main investigators of the study at the Otto von Guericke University Magdeburg and the German Center for Neurodegenerative Diseases.

For peer review only

For peer review only - http://bmjopen.bmj.com/site/about/guidelines.xhtml

2.7. Patient and public involvement

Subjects will be involved in the study as we will evaluate their acceptability for our measurement procedure. We will collect feedback on the study procedure from the participants. We will continue to work with stakeholders including the medical faculty and companies working in the field of neurodegeneration and dementia.

3. SUMMARY

Due to the demographic change and the increase of the individual life expectancy, the worldwide economic health care costs to treat individuals suffering from dementia will increase considerably. Currently, there is no treatment available which would allow to heal this neurological disease. Consequently, researchers now focus more on an early diagnosis of preclinical stages of AD (e.g., MCI) in order to initiate preventive actions timely. In this regard, neurophysiological signals could be promising biomarkers of such preclinical AD stages because they are, unlike behavioural performance, deemed to be less affected by learning or practice effects and provide insights into possible compensatory processes being not readily observable at the behavioural level. Hence, physiological signals can be helpful to detect changes in cognitive performance more precisely[124]. In this context, it is assumed that the investigation of new neurophysiological biomarkers, which allows to identify MCI more easily and accurately, are necessary to better understand and monitor the disease progression[12].

In line with the previous mentioned assumptions, the aim of this study is threefold. At first, this investigation aims to determine the differences in neurophysiological responses of HC and MCI participants. The second aim is to investigate possible neurobehavioral relationships between measures of cognitive performance and neurophysiological responses. The third aim is to elucidate whether a multimodal measurement approach can help to identify individuals with MCI more

For peer review only

accurately and reliable than a uni- or bimodal approach. Thereto, three complementary measuring modalities, namely fNIRS, EEG and ECG/HRV, will be used simultaneously to assess different neurophysiological responses which are associated with cognitive processes. To the best of our knowledge, this is the first study using these three modalities simultaneously in MCI patients and cognitive healthy controls. Given the explorative character of our study, our sample consists of 20 HC and 20 MCI patients whose neurophysiological signals will be recorded at a resting state and while performing three established cognitive tasks (Stroop, N-back and a verbal fluency test). We are aiming to detect certain neurophysiological parameters which are promising for an early identification of people which are at a higher risk of an overly age-related decline in cognitive performance (i.e., MCI detection).

In this regard, we hypothesize that the multimodal approach improves the classification accuracy between healthy controls and MCI patients as compared to a unimodal or a bimodal approach. If our hypothesis is verified, this study will pave the way for further research on multimodal measurement approaches for dementia research. Such upcoming research will use a larger sample size to examine the non-invasive biomarkers characterizing an early detection of non-physiological decline in cognitive performance in more detail.

Ethics and dissemination

Ethics approval was obtained for the study from the Ethics Committee of the Otto von Guericke University (reference: 83/19), and informed consent for participation will be obtained from all participants. Data will be shared with the scientific community no more than 1 year following completion of study and data assembly.

For peer review only

Additional file

Additional file 1: spatial registration for fNIRS optodes for assignment of fNIRS measurement channels.

Abbreviations

AD: Alzheimer's disease; ANS: autonomic nervous system; ApEn: approximate entropy; BA: Broadmann atlas; BDNF: brain-derived neurotrophic factor; BMI: body mass index; CSF: cerebrospinal fluid; deoxyHb: deoxygenated haemoglobin; DFA: detrended fluctuation analysis; ECG: electrocardiography; EEG: electroencephalography; ERP: event-related potential; ES: effect size; fNIRS: functional near-infrared spectroscopy; HAROLD: Hemispheric Asymmetric Reduction in OLD adults; HC: healthy controls; HF: power in high (0.15–0.40 Hz) frequencies; HRV: heart rate variability; ICA: independent component analysis; IZ: inion; Loreta: low-resolution electromagnetic tomography analysis; LPA: left preauricular point; MCI: mild cognitive impairment; MM: mean mastoid; MMSE: Mini-Mental State Exam; MRI: magnetic resonance imaging; MSE: mutliscale entropy; NZ: nasion; oxyHb: oxygenated haemoglobin; PCA: Principal Component Analysis; PET: positron emission tomography; pNN50: percentage of successive normal sinus NN intervals differing more than 50 ms; PSD: power spectral density; RMSSD: root mean square of the successive differences between adjacent normal NN intervals; RPA: right preauricular point; RPAn: recurrence plot analysis; SampEn: sample entropy; SD: standard deviation; SVM: support vector machine; VFT: verbal fluency test

For peer review only

References

1 2020 Alzheimer's disease facts and figures. Alzheimers Dement 2020.

2 WHO. Risk reduction of cognitive decline and dementia: WHO guidelines. Geneva: World Health Organization; 2019.

For peer review only - http://bmjopen.bmj.com/site/about/guidelines.xhtml

3 Dilling H, editor. Internationale Klassifikation psychischer Störungen: ICD-10 Kapitel V (F); klinisch-diagnostische Leitlinien. 6., vollst. überarb. Aufl. unter Berücksichtigung der Änderungen entsprechend ICD-10-GM 2004/2008. Bern: Huber; 2008. Available from: URL: http://d-nb.info/988424061/04.

4 Leicht H, Heinrich S, Heider D, et al. Net costs of dementia by disease stage. *Acta Psychiatr Scand* 2011;124(5):384–95.

5 Wallesch C-W, Förstl H, Bartels C. Demenzen. 3., unveränderte Auflage. Stuttgart, New York: Georg Thieme Verlag; 2017. (RRN, Referenz-Reihe Neurologie).

6 Wimo A, Guerchet M, Ali G-C, et al. The worldwide costs of dementia 2015 and comparisons with 2010. *Alzheimers Dement* 2017;13(1):1–7.

7 Etgen T, Sander D, Bickel H, et al. Mild cognitive impairment and dementia: the importance of modifiable risk factors. *Dtsch Arztebl Int* 2011;108(44):743–50.

8 Petersen RC, Smith GE, Waring SC, et al. Mild Cognitive Impairment: Clinical Characterization and Outcome. *Arch Neurol* 1999;56(3):303–8. Available from: URL: https://jamanetwork.com/journals/jamaneurology/fullarticle/774828.

9 Petersen RC. Mild cognitive impairment as a diagnostic entity. *J Intern Med* 2004;256(3):183–94.

10 Mitchell AJ, Shiri-Feshki M. Rate of progression of mild cognitive impairment to dementia--meta-analysis of 41 robust inception cohort studies. *Acta Psychiatr Scand* 2009;119(4):252–65.

11 Dubois B, Hampel H, Feldman HH, et al. Preclinical Alzheimer's disease: Definition, natural history, and diagnostic criteria. *Alzheimers Dement* 2016;12(3):292–323.

12 Jongsiriyanyong S, Limpawattana P. Mild Cognitive Impairment in Clinical Practice: A Review Article. *Am J Alzheimers Dis Other Demen* 2018;33(8):500–7.

13 Kivipelto M, Mangialasche F, Ngandu T. Lifestyle interventions to prevent cognitive impairment, dementia and Alzheimer disease. *Nat Rev Neurol* 2018;14(11):653–66.

For peer review only

For peer review only - http://bmjopen.bmj.com/site/about/guidelines.xhtml

14 Yiannopoulou KG, Papageorgiou SG. Current and Future Treatments in Alzheimer Disease: An Update. *J Cent Nerv Syst Dis* 2020;12:1179573520907397.

15 Atri A. The Alzheimer's Disease Clinical Spectrum: Diagnosis and Management. *Med Clin North Am* 2019;103(2):263–93.

16 Annavarapu RN, Kathi S, Vadla VK. Non-invasive imaging modalities to study neurodegenerative diseases of aging brain. *J Chem Neuroanat* 2019;95:54–69.

17 Pinti P, Tachtsidis I, Hamilton A, Hirsch J, et al. The present and future use of functional near-infrared spectroscopy (fNIRS) for cognitive neuroscience. *Annals of the New York Academy of Sciences* 2020;1464;5–29.

18 Herold F, Wiegel P, Scholkmann F, et al. Applications of Functional Near-Infrared Spectroscopy (fNIRS) Neuroimaging in Exercise–Cognition Science: A Systematic, Methodology-Focused Review. *J Clin Med* 2018;7(12).

19 Agbangla NF, Audiffren M, Albinet CT. Use of near-infrared spectroscopy in the investigation of brain activation during cognitive aging: A systematic review of an emerging area of research. *Ageing Research Reviews* 2017;38:52–66.

20 Fantini S, Ruesch A, Kainerstorfer JM. Noninvasive Optical Studies of the Brain. In: Shi L, Alfano RR, editors. Neurophotonics and biomedical spectroscopy. Amsterdam: Elsevier; 2018:25–52.

21 Scholkmann F, Kleiser S, Metz AJ, et al. A review on continuous wave functional near-infrared spectroscopy and imaging instrumentation and methodology. *NeuroImage* 2014;85;1;6–27.

22 Tachtsidis I, Scholkmann F. False positives and false negatives in functional near-infrared spectroscopy: issues, challenges, and the way forward. *Neurophotonics* 2016;3(3):30401.

23 Chou PH, Lan TH. The role of near-infrared spectroscopy in Alzheimer's disease. *Journal of Clinical Gerontology and Geriatrics* 2013;4(2):33–6.

For peer review only

For peer review only - http://bmjopen.bmj.com/site/about/guidelines.xhtml

24 Obrig H. NIRS in clinical neurology - a 'promising' tool? *NeuroImage* 2014;85;1:535–46.

25 Yeung MK, Chan AS. Functional near-infrared spectroscopy reveals decreased resting oxygenation levels and task-related oxygenation changes in mild cognitive impairment and dementia: A systematic review. *Journal of Psychiatric Research* 2020;124:58–76.

26 Katzorke A, Zeller JBM, Müller LD, et al. Decreased hemodynamic response in inferior frontotemporal regions in elderly with mild cognitive impairment. *Psychiatry Res Neuroimaging* 2018;274:11–8.

27 Yeung MK, Sze SL, Woo J, et al. Altered Frontal Lateralization Underlies the Category Fluency Deficits in Older Adults with Mild Cognitive Impairment: A Near-Infrared Spectroscopy Study. *Front Aging Neurosci* 2016;8:59.

28 Vermeij A, van Beek AHEA, Olde Rikkert MGM, et al. Effects of aging on cerebral oxygenation during working-memory performance: a functional near-infrared spectroscopy study. *PLoS ONE* 2012;7(9):e46210.

29 Nguyen T, Kim M, Gwak J, et al. Investigation of brain functional connectivity in patients with mild cognitive impairment: A functional near-infrared spectroscopy (fNIRS) study. *J Biophotonics* 2019;12(9):e201800298.

30 Niu H-J, Li X, Chen Y-J, et al. Reduced frontal activation during a working memory task in mild cognitive impairment: a non-invasive near-infrared spectroscopy study. *CNS Neurosci Ther* 2013;19(2):125–31.

31 Yang D, Hong K-S, Yoo S-H, et al. Evaluation of Neural Degeneration Biomarkers in the Prefrontal Cortex for Early Identification of Patients With Mild Cognitive Impairment: An fNIRS Study. *Front Hum Neurosci* 2019;13:317.

For peer review only

For peer review only - http://bmjopen.bmj.com/site/about/guidelines.xhtml

32 Yap KH, Ung WC, Ebenezer EGM, et al. Visualizing Hyperactivation in Neurodegeneration Based on Prefrontal Oxygenation: A Comparative Study of Mild Alzheimer's Disease, Mild Cognitive Impairment, and Healthy Controls. *Front Aging Neurosci* 2017;9:287.

33 Yoo S-H, Hong K-S. Hemodynamics Analysis of Patients With Mild Cognitive Impairment During Working Memory Tasks. Conf Proc IEEE Eng Med Biol Soc 2019;4470–3.

34 Cabeza R. Hemispheric asymmetry reduction in older adults: The HAROLD model. *Psychol Aging* 2002;17(1):85–100.

35 Michel CM, Murray MM. Towards the utilization of EEG as a brain imaging tool. *NeuroImage* 2012;61(2):371–85.

36 Hampel H, Lista S, Teipel SJ, et al. Perspective on future role of biological markers in clinical therapy trials of Alzheimer's disease: a long-range point of view beyond 2020. *Biochem Pharmacol* 2014;88(4):426–49.

37 Haroutunian V, Hoffman LB, Been MS. Is there a neuropathology difference between mild cognitive impairment and dementia? *Dialogues Clin Neurosci* 2009;11(2):171–9.

38 Jelic V, Kowalski J. Evidence-based evaluation of diagnostic accuracy of resting EEG in dementia and mild cognitive impairment. *Clin EEG Neurosci* 2009;40(2):129–42.

39 Scheff SW, Price DA, Schmitt FA, et al. Hippocampal synaptic loss in early Alzheimer's disease and mild cognitive impairment. *Neurobiol Aging* 2006;27(10):1372–84.

40 Al-Qazzaz NK, Ali SHBM, Ahmad SA, et al. Role of EEG as biomarker in the early detection and classification of dementia. *Scientific World Journal* 2014;2014:906038.

41 Choi J, Ku B, You YG, et al. Resting-state prefrontal EEG biomarkers in correlation with MMSE scores in elderly individuals. *Scientific Reports* 2019;9(1):10468.

For peer review only

For peer review only - http://bmjopen.bmj.com/site/about/guidelines.xhtml

42 Gouw AA, Stam CJ. Electroencephalography in the differential diagnosis of dementia. Epileptologie 2016; 33:173. Available from: URL: https://research.vumc.nl/en/publications/electroencephalography-in-the-differential-diagnosis-of-dementia.

43 Horvath A, Szucs A, Csukly G, et al. EEG and ERP biomarkers of Alzheimer's disease: a critical review. *Front Biosci (Landmark Ed)* 2018;23:183–220.

44 Yang S, Bornot JMS, Wong-Lin K, et al. M/EEG-Based Bio-Markers to Predict the MCI and Alzheimer's Disease: A Review From the ML Perspective. *IEEE Trans Biomed Eng* 2019;66(10):2924–35.

45 Sur S, Sinha VK. Event-related potential: An overview. *Ind Psychiatry J* 2009;18(1):70–3.

46 Sakkalis V. Review of advanced techniques for the estimation of brain connectivity measured with EEG/MEG. *Computers in Biology and Medicine* 2011;41(12):1110–7.

47 Sarmukadam K, Sharpley CF, Bitsika V, et al. A review of the use of EEG connectivity to measure the neurological characteristics of the sensory features in young people with autism. *Rev Neurosci* 2019;30(5):497–510.

48 Schoffelen J-M, Gross J. Source connectivity analysis with MEG and EEG. *Hum Brain Mapp* 2009;30(6):1857–65.

49 Schapkin SA, Raggatz J, Hillmert M, et al. EEG correlates of cognitive load in a multiple choice reaction task. *Acta Neurobiol Exp (Wars)* 2020;80(1):76–89.

50 Schomer DL, Lopes da Silva FH, editors. Niedermeyer's electroencephalography: Basic principles, clinical applications, and related fields. 7 ed. New York: Oxford University Press; 2017.

51 Laborde S, Mosley E, Thayer JF. Heart rate variability and cardiac vagal tone in psychophysiological research - Recommendations for experiment planning, data analysis, and data reporting. *Frontiers in Psychology* 2017;8(FEB).

For peer review only

For peer review only - http://bmjopen.bmj.com/site/about/guidelines.xhtml

52 McCraty R, Shaffer F. Heart rate variability: New perspectives on physiological mechanisms, assessment of self-regulatory capacity, and health risk. *Global Advances In Health and Medicine* 2015;4(1):46–61.

53 Billman GE. The LF/HF ratio does not accurately measure cardiac sympatho-vagal balance. *Frontiers in Physiology* 2013;4(FEB).

54 Draghici AE, Taylor JA. The physiological basis and measurement of heart rate variability in humans. *J Physiol Anthropol* 2016;35(1):22.

55 Shaffer F, McCraty R, Zerr CL. A healthy heart is not a metronome: an integrative review of the heart's anatomy and heart rate variability. *Frontiers in Psychology* 2014;5.

56 Fatisson J, Oswald V, Lalonde F. Influence diagram of physiological and environmental factors affecting heart rate variability: An extended literature overview. *Heart International* 2016;11(1):e32-e40.

57 Sammito S, Böckelmann I. Factors influencing heart rate variability. *ICFJ* 2016;6.

58 Thayer JF, Yamamoto SS, Brosschot JF. The relationship of autonomic imbalance, heart rate variability and cardiovascular disease risk factors. *International Journal of Cardiology* 2010;141(2):122–31.

59 Thayer JF, Lane RD. A model of neurovisceral integration in emotion regulation and dysregulation. *Journal of Affective Disorders* 2000;61(3):201–16.

60 Thayer JF, Hansen AL, Saus-Rose E, et al. Heart rate variability, prefrontal neural function, and cognitive performance: The neurovisceral integration perspective on self-regulation, adaptation, and health. *Annals of Behavioral Medicine* 2009;37(2):141–53.

61 Thayer JF, Lane RD. Claude Bernard and the heart-brain connection: Further elaboration of a model of neurovisceral integration. *Neuroscience and Biobehavioral Reviews* 2009;33(2):81–8.

For peer review only

For peer review only - http://bmjopen.bmj.com/site/about/guidelines.xhtml

62 Forte G, Favieri F, Casagrande M. Heart Rate Variability and Cognitive Function: A Systematic Review. *Front Neurosci* 2019;13:710.

63 Eggenberger P, Annaheim S, Kündig KA, et al. Heart Rate Variability Mainly Relates to Cognitive Executive Functions and Improves Through Exergame Training in Older Adults: A Secondary Analysis of a 6-Month Randomized Controlled Trial. *Front Aging Neurosci* 2020;12:197.

64 Grässler B, Hökelmann A, Cabral RH. Resting heart rate variability as a possible marker of cognitive decline. *Kinesiology (Zagreb, Online)* 2020;52(1):72–84.

65 Zeki Al Hazzouri A, Haan MN, Deng Y, et al. Reduced heart rate variability is associated with worse cognitive performance in elderly Mexican Americans. *Hypertension* 2014;63(1):181–7.

66 Kim DH, Lipsitz LA, Ferrucci L, et al. Association between reduced heart rate variability and cognitive impairment in older disabled women in the community: Women's Health and Aging Study I. *Journal of the American Geriatrics Society* 2006;54(11):1751–7.

67 da Silva VP, Ramalho Oliveira BR, Tavares Mello RG, et al. Heart Rate Variability Indexes in Dementia: A Systematic Review with a Quantitative Analysis. *Curr Alzheimer Res* 2018;15(1):80–8.

68 Delliaux S, Delaforge A, Deharo J-C, et al. Mental Workload Alters Heart Rate Variability, Lowering Non-linear Dynamics. *Front Physiol* 2019;10:565.

69 Mukherjee S, Yadav R, Yung I, et al. Sensitivity to mental effort and test-retest reliability of heart rate variability measures in healthy seniors. *Clin Neurophysiol* 2011;122(10):2059–66.

70 Sassi R, Cerutti S, Lombardi F, et al. Advances in heart rate variability signal analysis: Joint position statement by the e-Cardiology ESC Working Group and the European Heart Rhythm Association co-endorsed by the Asia Pacific Heart Rhythm Society. *Europace* 2015;17(9):1341–53.

71 Young H, Benton D. We should be using nonlinear indices when relating heart-rate dynamics to cognition and mood. *Scientific Reports* 2015;5:16619.

For peer review only

For peer review only - http://bmjopen.bmj.com/site/about/guidelines.xhtml

72 Fernandes de Godoy M. Nonlinear Analysis of Heart Rate Variability: A Comprehensive Review. *Journal of Cardiology and Therapy* 2016;3(3):528–33.

73 Bari V, Calcagnile P, Molteni E, et al. From neurovascular coupling to neurovascular cascade: a study on neural, autonomic and vascular transients in attention. *Physiol Meas* 2012;33(8):1379–97.

74 Ha U, Lee Y, Kim H, et al. A Wearable EEG-HEG-HRV Multimodal System With Simultaneous Monitoring of tES for Mental Health Management. *IEEE Trans Biomed Circuits Syst* 2015;9(6):758–66.

75 Chiarelli AM, Zappasodi F, Di Pompeo F, et al. Simultaneous functional near-infrared spectroscopy and electroencephalography for monitoring of human brain activity and oxygenation: a review. *Neurophotonics* 2017;4(4):41411.

76 Alba G, Vila J, Rey B, et al. The Relationship Between Heart Rate Variability and Electroencephalography Functional Connectivity Variability Is Associated With Cognitive Flexibility. *Front Hum Neurosci* 2019;13:64.

77 Hillmert M, Bergmüller A, Minow A, et al. Psychophysiologische Beanspruchungskorrelate während kognitiver Belastung. *Zbl Arbeitsmed* 2020:1–15. Available from: URL: https://link.springer.com/article/10.1007/s40664-020-00384-9.

78 Brugnera A, Adorni R, Compare A, et al. Cortical and Autonomic Patterns of Emotion Experiencing During a Recall Task. *Journal of Psychophysiology* 2018;32(2):53–63.

79 Aghajani H, Garbey M, Omurtag A. Measuring Mental Workload with EEG+fNIRS. *Front Hum Neurosci* 2017;11:359.

80 Ahn JW, Ku Y, Kim HC. A Novel Wearable EEG and ECG Recording System for Stress Assessment. *Sensors (Basel)* 2019;19(9).

81 Al-Shargie F, Tang TB, Kiguchi M. Stress Assessment Based on Decision Fusion of EEG and fNIRS Signals. *IEEE Access* 2017;5:19889–96.

For peer review only

For peer review only - http://bmjopen.bmj.com/site/about/guidelines.xhtml

802 82 Li R, Nguyen T, Potter T, et al. Dynamic cortical connectivity alterations associated with Alzheimer's
803 disease: An EEG and fNIRS integration study. *Neuroimage Clin* 2019;21:101622.

804 83 Li R, Potter T, Huang W, et al. Enhancing Performance of a Hybrid EEG-fNIRS System Using Channel
805 Selection and Early Temporal Features. *Front Hum Neurosci* 2017;11:462.

806 84 Liu Y, Ayaz H, Shewokis PA. Multisubject "Learning" for Mental Workload Classification Using
807 Concurrent EEG, fNIRS, and Physiological Measures. *Front Hum Neurosci* 2017;11:389.

808 85 Morioka H, Kanemura A, Morimoto S, et al. Decoding spatial attention by using cortical currents
809 estimated from electroencephalography with near-infrared spectroscopy prior information.
810 *NeuroImage* 2014;90:128–39.

811 86 Shin J, Kwon J, Im C-H. A Ternary Hybrid EEG-NIRS Brain-Computer Interface for the Classification
812 of Brain Activation Patterns during Mental Arithmetic, Motor Imagery, and Idle State. Front
813 *Neuroinform* 2018;12:5.

814 87 Faul F, Erdfelder E, Lang A-G, et al. G*Power 3: a flexible statistical power analysis program for the
815 social, behavioral, and biomedical sciences. *Behav Res Methods* 2007;39(2):175–91.

816 88 Sperling RA, Aisen PS, Beckett LA, et al. Toward defining the preclinical stages of Alzheimer's
817 disease: Recommendations from the National Institute on Aging-Alzheimer's Association workgroups
818 on diagnostic guidelines for Alzheimer's disease. *Alzheimers Dement* 2011;7(3):280–92.

819 89 Morris JC, Mohs RC, Rogers H, et al. Consortium to establish a registry for Alzheimer's disease
820 (CERAD) clinical and neuropsychological assessment of Alzheimer's disease. *Psychopharmacol Bull*
821 1988;24(4):641–52.

822 90 Barth S, Schönknecht P, Pantel J, et al. Neuropsychologische Profile in der Demenzdiagnostik: Eine
823 Untersuchung mit der CERAD-NP-Testbatterie. *Fortschr Neurol Psychiatr* 2005;73(10):568–76.

824 91 Fahrenberg J, Schönpflug W. Methoden der Aktivierungsforschung. Bern, Stuttgart, Wien: Hans
825 Huber; 1969. (Methoden der Aktivierungsforschung; vol 6).

For peer review only - http://bmjopen.bmj.com/site/about/guidelines.xhtml

92 Hancock PA, editor. Human mental workload. Amsterdam: North-Holland; 1988. (Advances in psychology; vol 52).

93 Longo L. On the Reliability, Validity and Sensitivity of Three Mental Workload Assessment Techniques for the Evaluation of Instructional Designs: A Case Study in a Third-level Course. In: Proceedings of the 10th International Conference on Computer Supported Education. *SCITEPRESS - Science and Technology Publications*;2018;166–78.

94 Stroop JR. Studies of interference in serial verbal reactions. *Journal of Experimental Psychology* 1935;18(6):643–62.

95 Bélanger S, Belleville S, Gauthier S. Inhibition impairments in Alzheimer's disease, mild cognitive impairment and healthy aging: effect of congruency proportion in a Stroop task. *Neuropsychologia* 2010;48(2):581–90.

96 Sun J, Sun B, Zhang L, et al. Correlation between hemodynamic and electrophysiological signals dissociates neural correlates of conflict detection and resolution in a Stroop task: a simultaneous near-infrared spectroscopy and event-related potential study. *J Biomed Opt* 2013;18(9):96014.

97 Mansouri C, Kashou NH. Spatial sensitivity of near-infrared spectroscopic brain imaging based on three-dimensional Monte Carlo modeling. *Conf Proc IEEE Eng Med Biol Soc* 2009;1457–60.

98 Badzakova-Trajkov G, Barnett KJ, Waldie KE, et al. An ERP investigation of the Stroop task: the role of the cingulate in attentional allocation and conflict resolution. *Brain Res* 2009;1253:139–48.

99 Egner T, Hirsch J. The neural correlates and functional integration of cognitive control in a Stroop task. *NeuroImage* 2005;24(2):539–47.

100 Compton RJ, Huber E, Levinson AR, et al. Is "conflict adaptation" driven by conflict? Behavioral and EEG evidence for the underappreciated role of congruent trials. *Psychophysiology* 2012;49(5):583–9.

101 Satish P, Muralikrishnan K, Balasubramanian K, et al. Heart rate variability changes during stroop color and word test among genders. *Indian J Physiol Pharmacol* 2015;59(1):9–15.

For peer review only

For peer review only - http://bmjopen.bmj.com/site/about/guidelines.xhtml

102 Baumert A, Buchholz N, Zinkernagel A, et al. Causal underpinnings of working memory and Stroop interference control: Testing the effects of anodal and cathodal tDCS over the left DLPFC. *Cogn Affect Behav Neurosci* 2020;20(1):34–48.

103 Kirchner WK. Age differences in short-term retention of rapidly changing information. *Journal of Experimental Psychology* 1958;55(4):352–8.

104 Herrmann MJ, Langer JBM, Jacob C, et al. Reduced prefrontal oxygenation in Alzheimer disease during verbal fluency tasks. *Am J Geriatr Psychiatry* 2008;16(2):125–35.

105 Aschenbrenner S, Tucha O, Lange KW. Regensburger Wortflüssigkeits-Test: RWT; Handanweisung. Göttingen: Hogrefe Verl. für Psychologie; 2000.

106 Heinzel S, Metzger FG, Ehlis AC, et al. Age and vascular burden determinants of cortical hemodynamics underlying verbal fluency. *PLoS ONE* 2015;10(9).

107 Jurcak V, Tsuzuki D, Dan I. 10/20, 10/10, and 10/5 systems revisited: their validity as relative head-surface-based positioning systems. *NeuroImage* 2007;34(4):1600–11.

108 Zimeo Morais GA, Balardin JB, Sato JR. fNIRS Optodes' Location Decider (fOLD): a toolbox for probe arrangement guided by brain regions-of-interest. *Scientific Reports* 2018;8(1):3341.

109 Rorden C, Brett M. Stereotaxic display of brain lesions. *Behav Neurol* 2000;12(4):191–200.

110 Pinti P, Scholkmann F, Hamilton A, et al. Current Status and Issues Regarding Pre-processing of fNIRS Neuroimaging Data: An Investigation of Diverse Signal Filtering Methods Within a General Linear Model Framework. *Front Hum Neurosci* 2018;12:505.

111 Huppert TJ, Diamond SG, Franceschini MA, et al. HomER: a review of time-series analysis methods for near-infrared spectroscopy of the brain. *Appl Opt* 2009;48(10):D280-98.

112 Brigadoi S, Ceccherini L, Cutini S, et al. Motion artifacts in functional near-infrared spectroscopy: a comparison of motion correction techniques applied to real cognitive data. *NeuroImage* 2014;85(0 1).

For peer review only

For peer review only - http://bmjopen.bmj.com/site/about/guidelines.xhtml

113 Scholkmann F, Wolf M. General equation for the differential pathlength factor of the frontal human head depending on wavelength and age. *Journal of Biomedical Optics* 2013;18(10):105004.

114 Jahani S, Fantana AL, Harper D, et al. fNIRS can robustly measure brain activity during memory encoding and retrieval in healthy subjects. *Scientific Reports* 2017;7(1):9533.

115 Lühmann A von, Li X, Müller K-R, et al. Improved physiological noise regression in fNIRS: A multimodal extension of the General Linear Model using temporally embedded Canonical Correlation Analysis. *NeuroImage* 2020;208:116472.

116 Kropotov ID. Quantitative EEG, event-related potentials and neurotherapy. 1st ed. Amsterdam, Boston, London: Elsevier/Academic; 2009. Available from: URL: http://site.ebrary.com/lib/alltitles/docDetail.action?docID=10254769.

117 Lei X, Liao K. Understanding the Influences of EEG Reference: A Large-Scale Brain Network Perspective. *Front Neurosci* 2017;11:205.

118 Malik M. Heart rate variability: Standards of measurement, physiological interpretation, and clinical use. *Circulation* 1996;93(5):1043–65.

119 Sammito S, Thielmann B, Seibt R, et al. Guideline for the application of heart rate and heart rate variability in occupational medicine and occupational science. *ASUI* 2015;2015(06).

120 Chen S-Y, Feng Z, Yi X. A general introduction to adjustment for multiple comparisons. *J Thorac Dis* 2017 9(6):1725–9.

121 Dahne S, Bieszmann F, Samek W, et al. Multivariate Machine Learning Methods for Fusing Multimodal Functional Neuroimaging Data. Proc. *IEEE* 2015;103(9):1507–30.

122 Hong K-S, Khan MJ, Hong MJ. Feature Extraction and Classification Methods for Hybrid fNIRS-EEG Brain-Computer Interfaces. *Front Hum Neurosci* 2018;12:246.

For peer review only

For peer review only - http://bmjopen.bmj.com/site/about/guidelines.xhtml

123 Murty MN, Raghava R. Support Vector Machines and Perceptrons. Cham: Springer International Publishing; 2016.

124 Ranchet M, Morgan JC, Akinwuntan AE, et al. Cognitive workload across the spectrum of cognitive impairments: A systematic review of physiological measures. *Neuroscience and Biobehavioral Reviews* 2017;80:516–37.

Authors' contributions

BG, FH, MD and TAG designed the study protocol. AH and NGM conceptualised and secured funding for the research study. BG, FH and MD contributed to the application to the local Ethics Committee. BG wrote the first draft of this publication with contributions from FH (fNIRS) and TAG (EEG). FH, MD, SD, NGM, IB and AH revised the initial draft. FH, TAG and SD settled the trial materials. IB, NGM and AH were responsible for the final study protocol. BG, FH, MD and TAG will participate in data collection. All authors read and approved the final manuscript.

Funding statement

This work is supported by the joint project "MyFit" funded by the European Regional Development Fund (grant number: ZS/2018/08/94206). The funder has no role in the design or conduct of the study.

Competing interests statement

The authors declare that they have no competing interests.

For peer review only

916 ## Legends of figures

917 Figure 4: IZ: inion; LPA: left preauricular point; NZ: nasion; RPA: right preauricular point.

918 Figure 5: ICA: independent component analysis. Loreta: Low-resolution electromagnetic tomography
919 analysis.

For peer review only

For peer review only - http://bmjopen.bmj.com/site/about/guidelines.xhtml

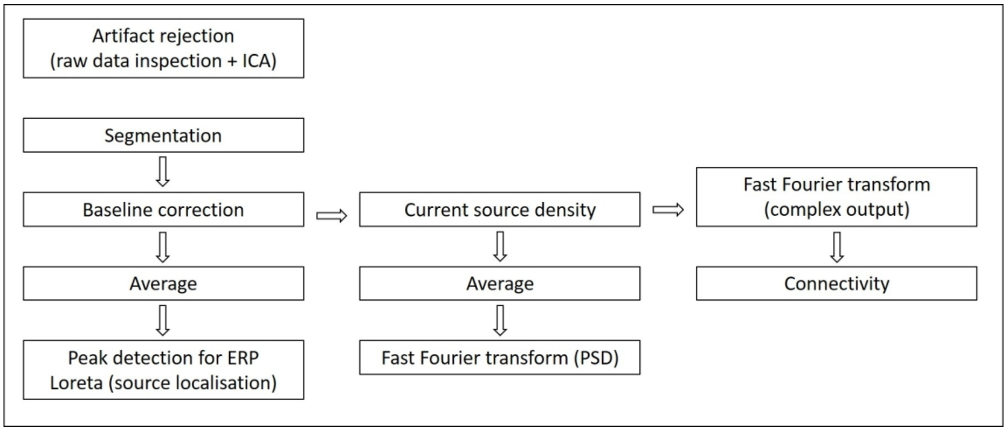

305x129mm (150 x 150 DPI)

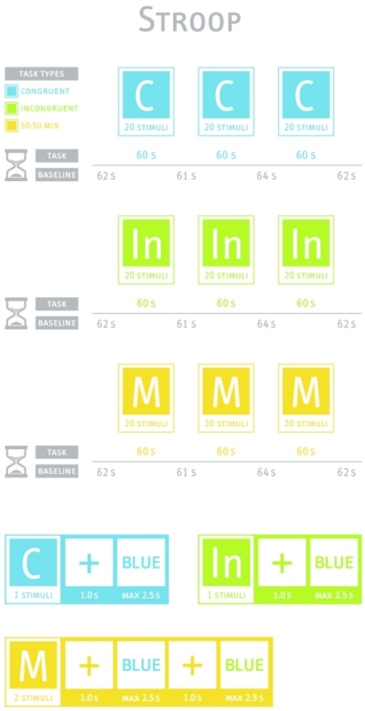

309x450mm (300 x 300 DPI)

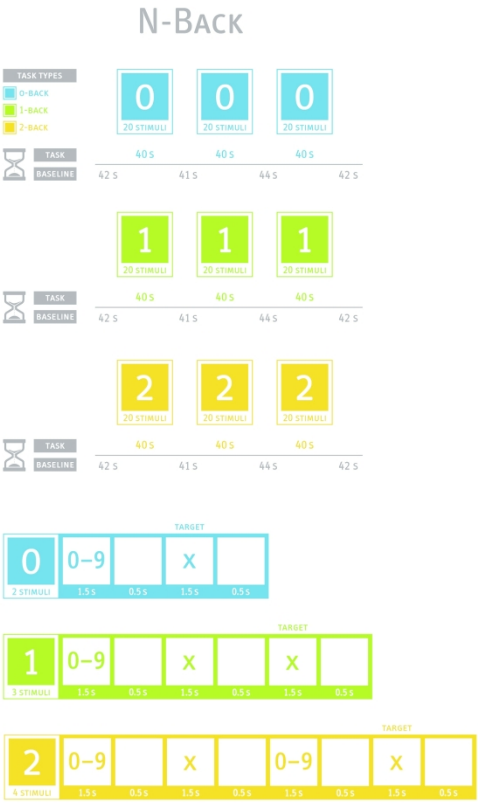

309x450mm (300 x 300 DPI)

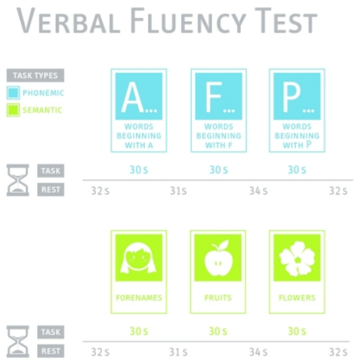

309x450mm (300 x 300 DPI)

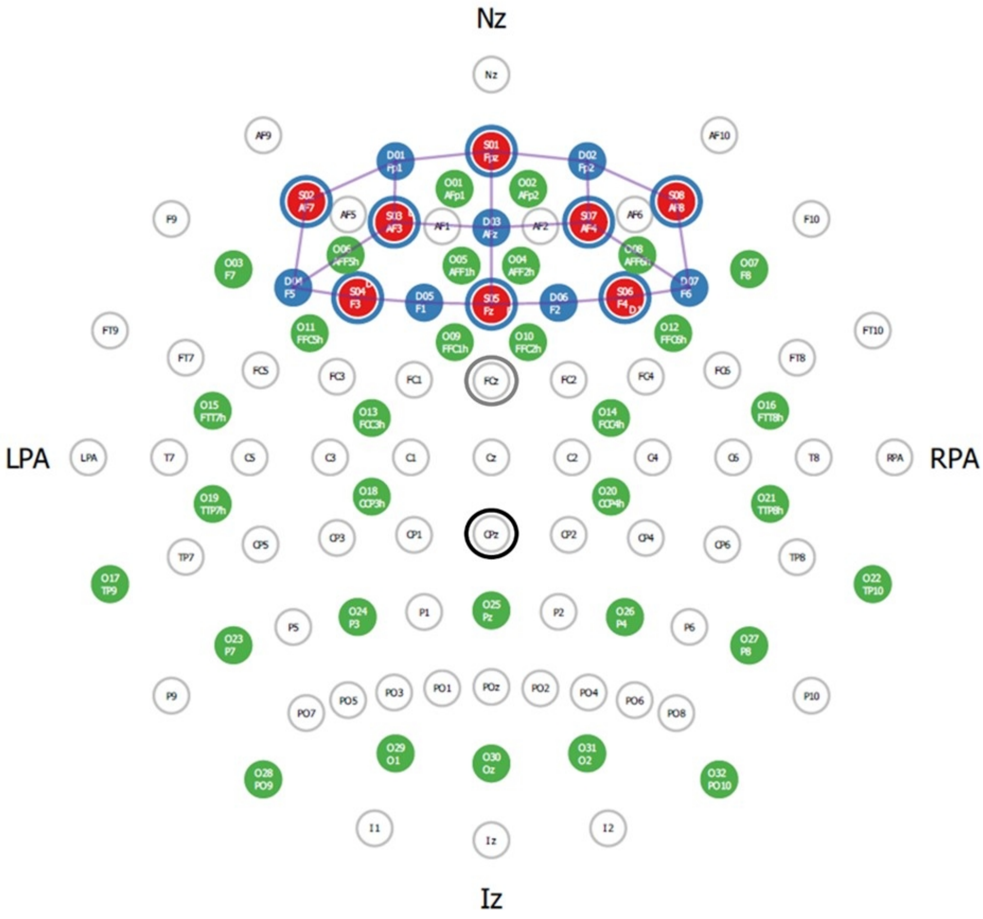

159x152mm (150 x 150 DPI)

Position	Source	Detector	X (mm)	Y (mm)	Z (mm)	Landmark	Specificity (%)	Distance (mm)
F4-F2	F4	F2	30	40	41	9 - Dorsolateral prefrontal cortex	68,36664315	30
						46 - Dorsolateral prefrontal cortex	22,39597886	
						8 - Includes Frontal eye fields	5,568764573	
F4-F6	F4	F6	46	38	24	45 - pars triangularis Broca's area	70,67253504	30
						46 - Dorsolateral prefrontal cortex	23,4516285	
AF8-F6	AF8	F6	48	46	5	45 - pars triangularis Broca's area	43,87699256	33
						46 - Dorsolateral prefrontal cortex	43,17605645	
						47 - Inferior prefrontal gyrus	5,07367444	
AF8-Fp2	AF8	Fp2	34	59	-2	10 - Frontopolar area	31,0841122	30
						11 - Orbitofrontal area	30,472822	
						46 - Dorsolateral prefrontal cortex	20,4016318	
AF4-F2	AF4	F2	22	52	33	9 - Dorsolateral prefrontal cortex	51,5248078	44
						46 - Dorsolateral prefrontal cortex	26,41541438	
						10 - Frontopolar area	18,40538603	
AF4-F6	AF4	F6	40	50	16	46 - Dorsolateral prefrontal cortex	47,36091434	45
						45 - pars triangularis Broca's area	30,60393436	
						10 - Frontopolar area	18,97467131	
AF4-Fp2	AF4	Fp2	25	63	9	10 - Frontopolar area	68,78124608	28
						11 - Orbitofrontal area	21,56708753	

For peer review only

						46 - Dorsolateral prefrontal cortex	6,609548215	
AF4-AFz	AF4	AFz	13	61	24	10 - Frontopolar area	72,46582734	36
						9 - Dorsolateral prefrontal cortex	16,97337784	
						46 - Dorsolateral prefrontal cortex	7,97503285	
Fpz-Fp2	Fpz	Fp2	13	67	0	10 - Frontopolar area	54,45817975	31
						11 - Orbitofrontal area	44,84702617	
Fpz-Afz	Fpz	AFz	1	64	14	10 - Frontopolar area	87,47712753	40
						9 - Dorsolateral prefrontal cortex	5,080149043	
Fpz-Fp1	Fpz	Fp1	-12	67	0	10 - Frontopolar area	54,49683736	30
						11 - Orbitofrontal area	44,89777168	
AF3-AFz	AF3	AFz	-12	62	23	10 - Frontopolar area	75,76225222	36
						9 - Dorsolateral prefrontal cortex	14,5484687	
						46 - Dorsolateral prefrontal cortex	8,053792322	
AF3-Fp1	AF3	Fp1	-24	63	9	10 - Frontopolar area	69,63399373	27
						11 - Orbitofrontal area	20,11557888	
						46 - Dorsolateral prefrontal cortex	8,793079404	
AF3-F5	AF3	F5	-39	50	17	46 - Dorsolateral prefrontal cortex	49,33992409	44
						45 - pars triangularis Broca's area	32,11689876	
						10 - Frontopolar area	16,03613047	

For peer review only

AF3-F1	AF3	F1	-23	52	32	9 - Dorsolateral prefrontal cortex	48,43964622	44
						46 - Dorsolateral prefrontal cortex	32,04248075	
						10 - Frontopolar area	16,88767141	
AF7-Fp1	AF7	Fp1	-33	59	-2	11 - Orbitofrontal area	32,71174196	30
						46 - Dorsolateral prefrontal cortex	25,27140585	
						10 - Frontopolar area	25,12699295	
						47 - Inferior prefrontal gyrus	13,10460546	
AF7-F5	AF7	F5	-47	46	6	45 - pars triangularis Broca's area	48,7862379	33
						46 - Dorsolateral prefrontal cortex	43,20372761	
F3-F5	F3	F5	-46	39	26	45 - pars triangularis Broca's area	72,56408005	29
						46 - Dorsolateral prefrontal cortex	21,83837451	
F3-F1	F3	F1	-31	39	41	9 - Dorsolateral prefrontal cortex	66,60628263	29
						46 - Dorsolateral prefrontal cortex	24,84615413	
Fz-F2	Fz	F2	10	41	50	9 - Dorsolateral prefrontal cortex	68,93306313	29
						8 - Includes Frontal eye fields	28,89129929	
Fz-AFz	Fz	AFz	2	50	39	9 - Dorsolateral prefrontal cortex	61,76828691	40
						10 - Frontopolar area	20,26301996	
						8 - Includes Frontal eye fields	12,14994939	
						32 - Dorsal anterior cingulate cortex	5,083608014	

For peer review only

Fz-F1	Fz	F1	-9	41	50	9 - Dorsolateral prefrontal cortex	63,16113601	29
						8 - Includes Frontal eye fields	34,73195285	

Please note that this output was generated using FOLD-software (G. A. Zimeo Morais, J. B. Balardin, and J. R. Sato, “fNIRS Optodes' Location Decider (fOLD): A toolbox for probe arrangement guided by brain regions-of-interest,” Scientific reports, vol. 8, no. 1, p. 3341, 2018.).

For peer review only

BMJ Open

Multimodal measurement approach to identify individuals with mild cognitive impairment: study protocol for a cross-sectional trial

Journal:	*BMJ Open*
Manuscript ID	bmjopen-2020-046879.R1
Article Type:	Protocol
Date Submitted by the Author:	09-Mar-2021
Complete List of Authors:	Grässler, Bernhard; Otto von Guericke Universitat Magdeburg, Institute for Sport Science<br>Herold, Fabian; German Centre for Neurodegenerative Diseases Site Magdeburg, Research Group Neuroprotection<br>Dordevic, Milos; German Centre for Neurodegenerative Diseases Site Magdeburg, Research Group Neuroprotection<br>Gujar, Tariq; Otto von Guericke Universitat Magdeburg, Institute for Sport Science<br>Darius, Sabine; Otto von Guericke University Medical Faculty, Occupational Medicine<br>Böckelmann, Irina; Otto von Guericke University Medical Faculty, Occupational Medicine<br>Müller, Notger; German Centre for Neurodegenerative Diseases Site Magdeburg, Research Group Neuroprotection; Otto von Guericke University Medical Faculty, Department of Neurology<br>Hökelmann, Anita; Otto von Guericke Universitat Magdeburg, Institute for Sport Science
**Primary Subject Heading**:	Neurology
Secondary Subject Heading:	Cardiovascular medicine, Complementary medicine, Diagnostics, Mental health, Research methods
Keywords:	CARDIOLOGY, MENTAL HEALTH, Physiology < NATURAL SCIENCE DISCIPLINES, Dementia < NEUROLOGY, Neurophysiology < NEUROLOGY

SCHOLARONE™
Manuscripts

I, the Submitting Author has the right to grant and does grant on behalf of all authors of the Work (as defined in the below author licence), an exclusive licence and/or a non-exclusive licence for contributions from authors who are: i) UK Crown employees; ii) where BMJ has agreed a CC-BY licence shall apply, and/or iii) in accordance with the terms applicable for US Federal Government officers or employees acting as part of their official duties; on a worldwide, perpetual, irrevocable, royalty-free basis to BMJ Publishing Group Ltd ("BMJ") its licensees and where the relevant Journal is co-owned by BMJ to the co-owners of the Journal, to publish the Work in this journal and any other BMJ products and to exploit all rights, as set out in our licence.

The Submitting Author accepts and understands that any supply made under these terms is made by BMJ to the Submitting Author unless you are acting as an employee on behalf of your employer or a postgraduate student of an affiliated institution which is paying any applicable article publishing charge ("APC") for Open Access articles. Where the Submitting Author wishes to make the Work available on an Open Access basis (and intends to pay the relevant APC), the terms of reuse of such Open Access shall be governed by a Creative Commons licence – details of these licences and which Creative Commons licence will apply to this Work are set out in our licence referred to above.

Other than as permitted in any relevant BMJ Author's Self Archiving Policies, I confirm this Work has not been accepted for publication elsewhere, is not being considered for publication elsewhere and does not duplicate material already published. I confirm all authors consent to publication of this Work and authorise the granting of this licence.

For peer review only

Multimodal measurement approach to identify individuals with mild cognitive impairment: study protocol for a cross-sectional trial

Bernhard Grässler (corresponding author)

Institute for Sport Science, Otto von Guericke University

bernhard.graessler@ovgu.de

Fabian Herold

Research Group Neuroprotection, German Center for Neurodegenerative Diseases (DZNE), Magdeburg, Germany

Milos Dordevic

Research Group Neuroprotection, German Center for Neurodegenerative Diseases (DZNE), Magdeburg, Germany

Tariq Ali Gujar

Institute for Sport Science, Otto von Guericke University

For peer review only - http://bmjopen.bmj.com/site/about/guidelines.xhtml

Magdeburg, Germany

Sabine Darius

Occupational Medicine, Faculty of Medicine, Otto von Guericke University

Magdeburg, Germany

Irina Böckelmann

Occupational Medicine, Faculty of Medicine, Otto von Guericke University

Magdeburg, Germany

Notger G. Müller

Research Group Neuroprotection, German Center for Neurodegenerative Diseases (DZNE), Magdeburg, Germany

Department of Neurology, Medical Faculty, Otto von Guericke University

Center for Behavioral Brain Sciences, Magdeburg, Germany

Magdeburg, Germany

Anita Hökelmann

Institute for Sport Science, Otto-von-Guericke University

Magdeburg, Germany

For peer review only

Word count

6519

ABSTRACT

Introduction

The diagnosis of mild cognitive impairment (MCI), i.e., the transitory phase between normal age-related cognitive decline and dementia, remains a challenging task. It was observed that a multimodal approach (simultaneous analysis of several complementary modalities) can improve the classification accuracy. We will combine three non-invasive measurement modalities: functional near-infrared spectroscopy (fNIRS), electroencephalography (EEG) and heart rate variability (HRV) via electrocardiography (ECG). Our aim is to explore neurophysiological correlates of cognitive performance and whether our multimodal approach can aid in early identification of individuals with MCI.

Methods and analysis

This study will be a cross-sectional with MCI patients (MCI) and healthy controls (HC). The neurophysiological signals will be measured during rest and while performing cognitive tasks: (i) Stroop, (ii) N-back and (iii) verbal fluency test. Main aims of statistical analysis are to (1) determine the differences in neurophysiological responses of HC and MCI, (2) investigate relationships between measures of cognitive performance and neurophysiological responses and (3) investigate whether the

For peer review only

classification accuracy can be improved by using our multimodal approach. To meet these targets, statistical analysis will include machine learning approaches.

This is, to the best of our knowledge, the first study that applies simultaneously these three modalities in MCI and HC. We hypothesize that the multimodal approach improves the classification accuracy between HC and MCI as compared to a unimodal approach. If our hypothesis is verified, this study paves the way for additional research on multimodal approaches for dementia research and fosters the exploration of new biomarkers for an early detection of non-physiological age-related cognitive decline.

Ethics and dissemination

Ethics approval was obtained from the local Ethics Committee (reference: 83/19). Data will be shared with the scientific community no more than 1 year following completion of study and data assembly.

Trial registration

ClinicalTrials.gov, NCT04427436, registered on 10 June 2020, https://clinicaltrials.gov/ct2/show/study/NCT04427436.

Keywords

Multimodal measurement approach, mild cognitive impairment (MCI) detection, neurodegenerative diseases, electroencephalography (EEG), functional near-infrared spectroscopy (fNIRS), heart rate variability (HRV), electrocardiography (ECG)

For peer review only

For peer review only - http://bmjopen.bmj.com/site/about/guidelines.xhtml

Strengths and limitations of this study

- This study will be the first to use a multimodal measuring approach to determine neurophysiological responses of elderly with mild cognitive impairment and healthy controls.
- Differences between these two groups will be investigated with electroencephalography, functional near-infrared spectroscopy and heart rate variability at resting state and while performing cognitive tasks.
- This study will provide valuable information about certain neurophysiological parameters which are promising for an early identification of people which are at a higher risk of an overly age-related decline in cognitive performance.
- We hypothesize that this multimodal approach improves the classification accuracy between elderly with mild cognitive impairment and healthy controls as compared to a unimodal or a bimodal approach.
- Since this study is the first of its kind, it has an exploratory character with a relatively small sample size, but it should provide a basic concept for large-scale studies.

1. INTRODUCTION

Mild cognitive impairment is considered as a prodromal stage of (or transition phase to) dementia. The most common cause of dementia is Alzheimer's disease (AD)[1]. It is estimated that approximately 50 million people are currently suffering from AD worldwide[2]. AD is a neurological disease with specific neuropathological (e.g., amyloid-plaques, neurofibrillary tangles) and neurochemical features (e.g., neurotransmitter deficits). On the behavioural level, AD is characterized by deficits of higher cortical functions such as memory, decision-making, visuospatial abilities, executive functioning, and language[3]. Individuals who suffer from dementia require a high level of care and support, and thus the disease entails a substantial burden for the health care systems[4]. For instance, in Germany, the annual costs are estimated at 42.6 billion Euros[5] whereas the global costs are considered to have

For peer review only

For peer review only - http://bmjopen.bmj.com/site/about/guidelines.xhtml

crossed the $1 trillion threshold in 2018[6]. Given that the number of individuals with dementia will increase further as a consequence of the demographic change, these annual health care expenditures are expected to grow, too.

In contrast to patients with dementia, patients with mild cognitive impairment (MCI) have only marginal limitations in activities of daily living, but show some deteriorations in specific cognitive domains[7]. A person is considered to suffer from MCI if the following criteria are fulfilled: memory complaint, normal activities of daily functioning, normal general cognitive function, abnormal memory for age, and not demented[8]. There are four types of MCI: amnestic MCI single domain, amnestic MCI multiple domain, non-amnestic MCI single domain and non-amnestic MCI multiple domain[9]. Patients with amnestic MCI show impairments in the performance of neuropsychological tests of episodic memory. Patients with non-amnestic MCI show impairments in cognitive domains other than memory, such as executive functions, language or visuospatial abilities[8]. For a correct detection of MCI, a careful and comprehensive neuropsychological test battery covering multiple cognitive domains is an important criterion[8]. Therefore, a correct detection of MCI by clinical data, regardless of whether single domain and multiple domain MCI are present, is relevant for our investigation. Hence, individuals with amnestic and non-amnestic MCI, based on a comprehensive clinical and neuropsychological assessment, without differentiating between single and multiple domain MCI, will be considered in our investigation. As these mild cognitive impairments are often not evident on a behavioural level, individuals with MCI are difficult to identify. During the stage of MCI, reduction of lifestyle-related risk factors, such as physical inactivity, may slow down or postpone the progression of this neurological syndrome[10-12]. So far, no drug has been approved for the symptomatic or causal treatment of MCI[13]. Nevertheless, an early diagnosis of MCI is indispensable for a better understanding of the neurobiological mechanisms mediating the transition of the inexorable age-related cognitive decline to dementia. This, in turn, may allow for a timely initiation of preventive strategies slowing, postponing or, at best, counteracting the transition to a serious manifestation of dementia.

For peer review only

For peer review only - http://bmjopen.bmj.com/site/about/guidelines.xhtml

The diagnosis of MCI based on clinical features alone is often challenging and relatively unreliable. Therefore, the identification of biomarkers that aid an early identification of neurodegenerative processes has become a focus of current research[14]. Neuroimaging methods such as magnetic resonance imaging (MRI) or positron emission tomography (PET) are popular tools in the investigation of neurodegenerative diseases. However, these methods have several disadvantages. They are expensive, have limited eligibility and the participant is not allowed to move. Additionally, the use of radioisotopes in PET results in exposure to ionizing radiation which involves detrimental health risks[15-16]. Biomarkers in the cerebrospinal fluid (CSF), such as Aβ42, constitute another approach for identifying AD pathology[1]. However, drawing CSF is stressful for the patient, can entail complications and does not necessarily yield unequivocal results. Thus, it is necessary to investigate other measurement methods able to detect a pathological cognitive decline timely while avoiding the above-mentioned limitations.

Functional near-infrared spectroscopy (fNIRS) is an optical neuroimaging technique that is based on the theory of neurovascular coupling and optical spectroscopy[17]. fNIRS allows the non-invasive investigation of cortical hemodynamic changes associated with brain activity[18]. In this regard, it is assumed that neuronal activity enhances the regional cerebral blood flow to the neuronal tissue at work which, in turn, leads to a local increase in the concentration of oxygenated haemoglobin (oxyHb) and decrease of deoxygenated haemoglobin (deoxyHb)[17]. Since oxyHb and deoxyHb have different light absorption spectra, the relative activity-dependent concentration changes of oxyHb and deoxyHb can be determined and allow us to make conclusions about cortical brain activity[16]. More details on physiological and methodological background of fNIRS can be found in the referenced literature[17, 19-20].

The advantages of fNIRS are its portability, tolerance to motion artefacts (compared to EEG and MRI), higher temporal resolution (compared to MRI), low costs (compared to MRI and PET), it is easy to use and more participants are eligible than for MRI (e.g., no restriction due to claustrophobia or metallic implants). The disadvantages are its lower spatial resolution (compared to MRI), low penetration

For peer review only - http://bmjopen.bmj.com/site/about/guidelines.xhtml

depth, lack of standardization in data analysis and, due to the delay in the hemodynamic response, there is a time delay in the signal curve, too[16].

fNIRS has become a frequently used technique for the investigation of neurological diseases such as AD[21-23]. There is evidence in the literature showing that fNIRS offers a great potential to become a valuable tool to identify individuals with a higher risk of developing dementia as changes in cortical hemodynamic obtained during a standardized cognitive test allow to discern between healthy and diseased individuals[24-31]. Young participants showed a stronger activation in the left hemisphere in the more difficult task conditions. Elderly participants showed no lateralization and a decreased activation in the difficult task condition. This phenomenon is called “Hemispheric Asymmetric Reduction in OLD adults” (HAROLD) model[32].

Electroencephalogram (EEG) is another technique for quantifying neurophysiological processes. EEG measures electrical activity provoked by the firing of cortical neurons within the brain. EEG is an optimal tool for recording the complex dynamic neural activity due to the excellent temporal resolution in milliseconds and reasonable spatial resolution[33]. The potential to detect pre-dementia AD/MCI condition has already been shown[34-37].

In addition to the most commonly used method of frequency analysis, it is also recommended to apply other more sophisticated EEG methods, like brain mapping, connectivity analysis or the analysis of event-related potentials, which can lead to a further specification of the diagnosis of dementia[38-42].

The specific neural activity in response to a certain stimulus can be measured by event-related potentials (ERPs). The most common component we can find in response to sensory, cognitive, or motor stimuli are P100, P200, P300, P600, N100 and N400, which are used in cognitive science (see for review, [43]). Importantly, it is not sufficient to study the cortical modulation in isolation. Cortical functions are influenced by functional integration. EEG is an optimal technique to understand the communication between different areas of the brain by functional and effective connectivity analysis. Functional connectivity is defined as the correlation between different areas of the brain. Effective

For peer review only

For peer review only - http://bmjopen.bmj.com/site/about/guidelines.xhtml

connectivity is defined as direct or indirect influence that one neural system exerts on the other neural system. The connectivity can be investigated by sources and EEG channels[44-46]. Beside connectivity, it is also possible to construct the brain map from the recording of electrical potentials with electrode distribution over the skull. The brain mapping can be helpful to identify activated structures of the cortex during task execution[47-48].

Electrocardiography (ECG) will be the third assessment method. In particular, we will determine the heart rate variability (HRV) from the ECG. The HRV describes the beat-to-beat variation of subsequent NN-intervals. These fluctuations in the heart rate are regarded as an indicator of the functional state of the autonomic nervous system (ANS) and is related to psychophysiological aspects such as self-regulation on a cognitive, emotional, social, and health level[49-50]. HRV is the result of the complex interaction between sympathetic and parasympathetic influences[51-52]. A relatively low HRV indicates an increased sympathetic state, an abnormal regulation and inadequate adaptation of the cardiovascular system, and is a sign of depletion or pathology. Hence, HRV is a promising marker to diagnose pathological states[52] and an optimal HRV is critical to health and well-being[53].

HRV measurements provide the advantages that they are easy to use, not expensive, portable, non-invasive and the recordings are relatively easy to interpret. However, on the downside HRV values are strongly influenced by individual differences, there is a relatively large number of influencing factors that have to be controlled for, and there is still no generally accepted consensus concerning data acquisition and data processing[49, 54-55].

Measures of HRV have been used to index vagal activity[56]. Root mean square of successive differences between adjacent normal NN intervals (RMSSD), percentage of successive normal NN intervals differing more than 50 ms (pNN50) and power in high (0.15–0.40 Hz) frequencies (HF) are supposed to reflect vagally mediated HRV[49, 53, 57]. According to the neurovisceral integration model, neural structures responsible for affective, cognitive and physiological regulation are associated with vagally mediated cardiac function[58]. Especially the prefrontal cortex is associated

For peer review only - http://bmjopen.bmj.com/site/about/guidelines.xhtml

with HRV measures as it is connected with the amygdala and cardiovascular system[59]. Vagally mediated HRV is supposed to be linked with “a set of neural structures that have been implicated in cognitive, especially executive function”[58]. A positive connection between resting HRV and cognitive functioning has already been demonstrated[60-64]. In a recent meta-analysis, the effect size (ES) magnitude of HRV measures in the evaluation of autonomic dysfunction in older people with dementia was investigated[65]. Although the small ES does not support the use of HRV as a single biomarker to diagnose dementia, the results suggest autonomic dysfunction in dementia. Since biological processes such as ANS activity are complex and non-linear, several authors suggest non-linear HRV measures[66-70]. In this context, Sample Entropy recorded at rest, was related to a better cognitive performance, but traditional time or frequency indices were not[69]. The sensitivity and reliability of non-linear measures such as Poincaré and detrended fluctuation analysis (DFA) for mental effort tasks could be proved in healthy seniors[67]. In a recent study, D2, the RRI dimension correlation, could be better related to the mental workload than time or frequency indices[66].

Cognition relies on complex neurophysiological processes. In particular, the solving of a cognitive task is associated with task-related changes in cerebral hemodynamics, cerebral electrical activity and changes in the ANS. These task-related neurophysiological changes can be assessed by fNIRS, EEG and HRV[71] separately (unimodal approach) or simultaneously (multimodal approach). A clear advantage of the multimodal assessment approach of task-related neurophysiological changes (e.g., cognition-related brain activity) is its ability to reduce and/or to compensate for inherent limitations of a single measurement modality (e.g., artefacts that are reflected in only one modality)[72]. As single tests, they might be not accurate enough for differentiating healthy controls from cognitive impaired participants. EEG, fNIRS, and HRV provide complementary information about different neurophysiological systems which, in turn, foster an improvement of the classification accuracy. Indeed, cognitive neuroscience research now focuses on the simultaneous acquisition by non-invasive modalities to improve their performance and information content[15]. Combined fNIRS-EEG[73], EEG-HRV[74-75] and fNIRS-HRV[76] measurements were already applied in different fields of brain research. It was observed that

For peer review only - http://bmjopen.bmj.com/site/about/guidelines.xhtml

classification accuracy can be improved when using different measurement modalities simultaneously[77-84]. However, so far and to the best of our knowledge, there is no published study available which uses the above-described multimodal approach to investigate the neurophysiological cognition-related differences between healthy older individuals and older individuals with MCI.

The primary aim of this study is to investigate the difference between healthy controls and cognitively impaired participants in neurophysiological signals. We will use fNIRS, EEG and ECG since they are non-invasive and provide some advantages compared to MRI, PET and measures of cerebrospinal fluid. As the most of the currently available studies used, to the best of our knowledge, only two different measurement modalities (mostly EEG and fNIRS), we hypothesize that our approach improves classification accuracy considerably. The second aim of this study is to investigate possible neurobehavioral relationships between measures of cognitive performance and measures of fNIRS, EEG, and HRV. The third aim of this study is to systematically compare the classification accuracy of uni-, bi- and multimodal measurement approaches. By saying that, we wish to emphasize that this study has an explorative character aiming to identify cognition-related neurophysiological parameters that are promising for MCI detection. This study should provide a basic concept for further studies using a multimodal measurement approach and promote research for a better understanding of the neurobiological mechanisms leading to dementia.

2. METHODS AND ANALYSIS

2.1. Study design

This cross-sectional study will be conducted by a multidisciplinary team of researchers from Department of Sport Science at the Otto von Guericke University Magdeburg, the Medical Faculty of the Otto von Guericke University Magdeburg and the German Center for Neurodegenerative Diseases. The study protocol was approved by the Ethics Committee of the Otto von Guericke University

For peer review only - http://bmjopen.bmj.com/site/about/guidelines.xhtml

259 Magdeburg (reference: 83/19) and is in accordance with the Declaration of Helsinki. This study was registered in ClinicalTrials.gov (NCT04427436) on the 10th June 2020.

2.2. Participants

This study has an explorative character as ther is, to the best of our knowledge, no comparable study available which used measures from multiple modalities (e.g., EEG, fNIRS, HRV) to differentiate between individual suffering from MCI and HC. Thus, we considered studies comparing individuals with MCI and HC using, at least, one measurement modality[29, 85, 86] for our sample size estimation. In this context, the following calculations has been performed.

Based on the means of oxyHb of individuals with MCI and HC during a cognitive task in the study of Niu et al. (2013) [29], a sample size of 15 participants in each group will be needed to achieve a statistical power of 80%. Based on the HF nu values during standing position in one HRV-study[85], a sample size of 49 participants in each group will be needed to achieve a statistical power of 80%. Finally, based on the latency of P300 during an event-related task in one EEG study[85], a sample size of 34 participants in each group will be needed to achieve a statistical power of 80%. All sample sizes were calculated a priori by using G*Power 3.1[87]. Furthermore, studies which had applied EEG, fNIRS and ECG simultaneously in a cohort of healthy subjects[71, 82, 88] used sample sizes varying between 11 and 25 subjects. Given the explorative character of this study and the intention to pave the way for future investigations with a larger sample size, we chose a relatively small sample with 30 MCI subjects and 30 HC constituting a tempered and conservative estimate to detect possible neurophysiological effects and/or trends.

This study will involve MCI patients who have been diagnosed by an experienced neurologist, based on standardized clinical and neuropsychological criteria[89]. Healthy participants will be recruited by advertisements in local newspapers. Interested individuals will be informed about the aim of the study and first be screened by telephone to check for general eligibility according to our criteria. The two groups will be age, gender-, handedness- and education-matched. At the beginning of the study, each

For peer review only - http://bmjopen.bmj.com/site/about/guidelines.xhtml

participant will provide a personally signed and dated informed consent document indicating that the individual has been informed of all pertinent aspects of the study. Participants must be native German-speaking adults who are between 55 and 80 years old. All participants will be financially rewarded to compensate for their participation.

Exclusion criteria are:

- other neurological diseases (i.e., epilepsy, multiple sclerosis)
- known severe cardiac diseases (i.e., history of heart disease, severe cardiac insufficiency, heart failure, cardiac pacemaker, valvular defect, with or without stent implantation, heart attack)
- stroke
- mental diseases (i.e., schizophrenia, depression)
- orthopaedic diseases (i.e., bone fracture in last six months, symptomatic slipped disc)
- muscular diseases (i.e., myositis, tendovaginitis)
- severe endocrinologic diseases (i.e., manifest hypothyroidism or hyperthyroidism, adiposity [BMI >30], juvenile-onset diabetes)
- injury or surgery in last six months
- use of illegal intoxicants or alcohol abuse (more than three times per week)
- uncorrected poor eyesight or hearing
- colour blindness / red-green weakness
- pregnancy or breastfeeding
- using one of the following medications: betablocker, ACE inhibitor, antiarrhythmic drugs, neuroleptics, narcotic analgesics, benzodiazepines and psychoactive medications

At the beginning of the study, all participants will be screened by using the CERAD test battery[90]. This cognitive test battery evaluates the performance in semantic verbal fluency, word retrieval, constructional praxis, visual memory, verbal memory, global cognition (Mini Mental State Examination

For peer review only

For peer review only - http://bmjopen.bmj.com/site/about/guidelines.xhtml

[MMSE]) and motor speed (Trail Making Test A). The CERAD has been shown to be a valid and reliable assessment tool to identify individuals with cognitive impairments[91].

2.3. Assessments

Participants will attend three visits within one week and undergo the following assessments:

1. clinical assessment: CERAD and medical examination, taking blood sample [i.a. Apolipoprotein 4 (ApoE4) and BDNF (brain-derived neurotrophic factor)]
2. sociodemographic assessment: questionnaires
3. neurophysiological and neuropsychological assessment: EEG/fNIRS/ECG simultaneously at resting state and during cognitive tasks

For peer review only

2.4. Sociodemographic assessment

Sociodemographic data, family history of AD/dementia, and lifestyle-factors of the participants will be obtained before the measurements via several questionnaires. We will record medication, together with measurements of height, weight, educational and physical activity level. The level of physical activity will be assessed via the questionnaire German-PAQ50+[92]. It will allow for this factor to be considered as a covariate. The health-related quality of life will be measured by the 36-Item Short Form Health Survey[93]. Sleep quality will be assessed via the Pittsburgh Sleep Quality Index[94]. Finally, the Food Frequency Questionnaire will be applied to assess the dietary habit of the participants[95].

2.5. Neurophysiological and neuropsychological assessment

Research staff collecting data are blinded concerning the cognitive status (MCI or HC) of the participant to avoid bias. EEG, fNIRS and ECG will be recorded simultaneously at resting state for ten minutes. The

For peer review only - http://bmjopen.bmj.com/site/about/guidelines.xhtml

measurement standards for the resting state measurement follows the recommendations of Laborde et al.[49]. Accordingly, the participants are asked to sit on a comfortable chair with their knees bent at a 90° angle and their hands on their thighs. Furthermore, they are advised to relax, breath normally and move as little as possible. As recommended in the literature, the participants will rest in the above-described position for at least five minutes before the baseline recordings are obtained[49]. Subsequently, the participants complete three cognitive tasks while their neurophysiological signals are measured. The cognitive tasks are Stroop, N-back and a verbal fluency test (VFT). This procedure is comparable to the study of Yang et al.[29] but in addition to this study (i) we will conduct the assessment of EEG and ECG and (ii) we will use modified versions of the cognitive tasks. Afterwards, a second resting-state measurement will be conducted (recovery phase)[49].

According to the multistage concept in psychophysiology of Fahrenberg, objective data of the cognitive performance, objective physiological data and subjective data of the personal feeling will be collected[96]. The latter will be recorded by the NASA-TLX questionnaire. The NASA-TLX provides an overall workload score with six dimensions: mental demands, physical demands, temporal demands, own performance, effort, and frustration[97]. The overall workload is the weighted average of these dimensions. After the cognitive tests, participants will be instructed to rate each dimension on a visual scale from 1 to 20 points. It is an easy to use tool and its results can be compared with participants` performance and their neurophysiological responses. The NASA-TLX is a widely used tool with a high reliability and validity[98].

2.5.1. Cognitive tasks

During the whole assessment, the participants are sitting in front of a computer screen on a comfortable chair and are asked to avoid head movements as much as possible. Before the experiment, the participants will be briefed on the task instructions and experimental design. The instructions are presented on a printed paper and explained verbally by the investigator. Each task

For peer review only

block begins with a 5 s instruction cue that informs the participant about the task condition. All tasks will be administered via computer which allows to measure reaction times and responses of the participants exactly. To synchronize all signals, temporal triggers are delivered simultaneously to EEG and fNIRS systems via the software Presentation (Neurobehavioral Systems Inc, USA).

2.5.1.1. Stroop

A computerized version of the Stroop test will be applied[99]. This widely used test requires the executive functions inhibition and cognitive control. These are crucial for completion of complex cognitive tasks and everyday activities[100]. The classic Stroop test elicits a conflict situation since the meaning of a colour-word and the ink colour do not match. Behavioural responses to these incongruent stimuli are usually slower and less accurate than responses to congruent stimuli. The behavioural difference between incongruent and congruent stimuli is called the Stroop effect, which has been used as an index of cognitive control[101]. Since the prefrontal cortex plays a predominant role in cognitive control[102] and have shown to be affected by MCI[23], fNIRS optodes will be placed on the prefrontal cortex.

Our Stroop test includes three experimental conditions (pure congruent, pure incongruent and mixed congruent and incongruent stimuli) with three blocks in each condition (see Figure 1). Prior to each block, a baseline measurement having nearly the same length as the task blocks will be applied in order to assess a baseline for the hemodynamic activity. During the baseline measurement, participants are requested to sit still and relax. According to recent recommendations[17], the duration of the baseline measurement should not be a multiple of 10 s to avoid the overlap with the Mayer waves. In each block, colour-words are consecutively presented in the middle of the computer screen. A black background was chosen to avoid overstraining the eyes. Each colour has a corresponding button. Four different colour-words will appear: “RED”, “GREEN”, “BLUE” or “YELLOW” in German language. In the congruent condition, the meaning of the colour-word and the ink colour match. In the incongruent condition, the colour-word is printed in an incongruent ink colour, e.g., the word "RED" is presented

For peer review only - http://bmjopen.bmj.com/site/about/guidelines.xhtml

in blue colour. Participants are instructed to identify the colour of the word by pressing the appropriate button and not reacting on the meaning of the word. In this example, the participant must press the button for the ink colour (blue). Participants should react as fast and as correct as possible. In the third block, congruent and incongruent stimuli are presented. Participants are instructed to react on the colour of the word. Although the typical clinical version of the Stroop tests include a pure block of neutral (congruent) stimuli and a pure block of incongruent stimuli, some authors criticise this pure-block design since it has some limitations[100]. Instead, a mixed block design with congruent stimuli within an incongruent condition challenge goal-maintenance capacities because the congruent stimuli promote the inappropriate but more automatically response of reading the word and produce larger Stroop effects than pure incongruent blocks[100]. This mixed design was already used in some studies[103-107]. Hence, we will use a pure incongruent and a mixed block. The mixed block consists of 50 % congruent and 50 % incongruent stimuli. The participants should react in both variants to the colour of the word. Prior to task, the participants will be adequately familiarized with the test by performing a sufficient number of practice trials.

2.5.1.2. N-back

The N-back was first introduced by Kirchner[108] and is a frequently used task to measure working memory capacity. In our study, three conditions (0-, 1- und 2-back) will be used (see Figure 2). Single-digit numbers with a presentation time of 1500 ms are presented consecutively in three blocks in the middle of the screen. The interstimulus interval is 500 ms. As in the Stroop test, a baseline measurement will be included prior to each task block and after the last block.

In the 0-back condition, the participants are asked to press the target button only when the number "7" appears. All other numbers must be ignored. In the 1-back condition, the participants are asked to press the target button only when two identical numbers appear in succession. In the 2-back condition, the participants are asked to press the target button only when the current number matches the second-last number displayed before. In all three conditions, 25 % of stimuli are targets. As in the

For peer review only - http://bmjopen.bmj.com/site/about/guidelines.xhtml

406 Stroop test, participants will be instructed to react as fast and as correct as possible. Prior to task, the participants will be adequately familiarized with the test by completing a sufficient number of practice trials.

2.5.1.3. *Verbal fluency test*

Verbal fluency is a cognitive domain that worsens with the development of Alzheimer's disease[109]. Therefore, we plan to use a verbal fluency test (VFT) which is based on the "Regensburger Wortflüssigkeitstest"[110]. This test is frequently used in fNIRS studies investigating neurological diseases[24, 30, 109, 111]. Based on these studies, our test consists of two conditions. Both conditions will be presented three times in a row for 30 s with a resting block for 31–34 s between each block (see Figure 3). During the resting blocks, the participants are requested to sit still and avoid speaking. We use a phonological (letter) and a semantic (category) condition. The instructions will be displayed on a screen to avoid instruction bias. Participants are requested to avoid movements during this test.

In the phonological condition, the participants are instructed to pronounce as many German words (nouns, verbs, adjectives) as possible beginning with a specific letter ("A", "F", "P") without using proper names, numbers, repetitions or words in different forms or with different endings. In the semantic condition, participants are instructed to pronounce as many words as possible belonging to a specific category ("forenames", "fruits", "flowers").

2.5.2. Neurophysiological assessment

2.5.2.1. *fNIRS*

In this study, a portable continuous wave fNIRS system (NIRSport™, NIRx Medical Technologies, Glen Head, New York, USA) will be used to record cortical hemodynamics with a frequency of 10.2 Hz. The fNIRS-system consists of the following: eight light sources which emit light at wavelengths of 760 and 850 nm, eight light detectors, and a short-distance detector bundle (NIRx Medical Technologies, Glen Head, New York, USA) which allows to quantify changes in extracerebral layer (i.e., blood flow in the

For peer review only - http://bmjopen.bmj.com/site/about/guidelines.xhtml

scalp). As shown in Figure 4, the fNIRS optodes will be positioned according to the 10–20 EEG system[112] by using a standardized cap (EasyCap GmbH, Herrsching, Germany). We will perform a virtual and probabilistic spatial registration using the software fOLD (fNIRS Optodes' Location Decider)[113] and the Broadmann atlas (BA)[114] to assign the fNIRS measurement channels with long source-detector separation to specific brain regions (see Additional file 1 for detailed overview).

The data processing of fNIRS follows recent recommendations[17, 115, 116] and will be conducted using the latest version of the "Homer" software package[117]. In brief, we will conduct the following processing steps: (i) exclude noisy channels by using enPruneChannels function, (ii) convert raw light intensity changes into changes in optical density by using hmrIntensity2OD function, (iii) perform motion artefact correction by using sophisticated filter methods (e.g., wavlet filter - hmrMotionCorrectWavelet filtering function)[118], (iv) perform a correction for physiological artefacts such as heart beat and instrumental noise by using hmrBandpassFilt function, (iv) convert optical density data of both wavelengths via the modified Beer–Lambert law (MBLL) into concentration changes of oxyHb and deoxyHb by using the hmrOD2Conc function and an individually calculated differential path length factor[119], (v) correct for extracerebral blood flow by using hmrDeconvHRRF_DriftSS function[120-121], and (vi) perform a baseline correction and calculation of block averages for oxyHb and deoxyHb changes over all trials and for each measurement channel by using hmrBlockAvg function. In the final step, the pre-processed time-series of oxyHb and deoxyHb are exported and the cognition-related changes in these two chromophores are used for further statistical analysis.

For peer review only

2.5.2.2. EEG

Electroencephalography data will be acquired by the Brain Vision wireless MOVE system (Brain Products GmbH, Munich, Germany) along with Brain Vision Recorder Version 1.21.0102 (Brain Products GmbH, Munich, Germany). The system will be triggered by the computer running the Presentation software. The EEG/fNIRS cap will be customized according to the 10–20 system with the

For peer review only - http://bmjopen.bmj.com/site/about/guidelines.xhtml

provision of fNIRS optodes and 34 EEG channel slim active electrodes [AFp1, AFp2, AFF5h, AFF1h, AFF2h, AFF6h, F7 , F8, FFC5h, FFC1h, FFC2h, FFC6h, FTT7h, FCC3h, FCC4h, FTT8h, TTP7h, CCP3h, CCP4h, TTP8h, TP9, TP10, P7, P3, Pz, P4, P8, Po9, O1, Oz, PO10, Fz (reference) and ground][81]. The electrode location will be customized in the Brain Vision Recorder workspace according to the EEG elastic cap channel position in consideration of Theta and Phi values[122]. The impedance will be kept below 5 KΩ before EEG data recording. The cognitive tasks will be recorded following a baseline measurement of ten minutes. The EEG data will be recorded at 1.000 Hz. The outcome of the results will be focused on time and frequency domain of ERPs, power spectrum analysis, source localization and connectivity.

The EEG recorded data will be analysed by the Brain Vision Analyzer 2.2.0 (Brain Products GmbH, Munich, Germany). The EEG post-processing will be divided into two blocks whereas P100, P200, P300, P600, N100 and N400 ERPs[2], PSD (power spectral density; frequency analysis), connectivity and source localisation will be done for the N-back and Stroop, and PSD will be done for the VFT. The pre-recorded EEG data will be resampled at 256 Hz and will be re-referenced to mean mastoids (MM)[123]. The referenced data will be filtered for ERPs at high pass 0.1 Hz and low pass at 40 Hz with 50 Hz notch filter. For PSD, connectivity and source localisation a low pass filter of 85 Hz will be used. The EEG data processing pipeline after re-referencing and filtering is shown in Figure 5.

2.5.2.3. ECG

We will record the ECG of the participants during the experimental session with an ECG medilog AR12 plus (Schiller, Baar, Switzerland). This device consists of three channels with a sampling rate of 1.000 Hz. By using this device, we can record the NN intervals in millisecond range which enables us to accurately calculate the HRV and to precisely identify cardiac arrhythmia. The raw ECG data will be uploaded to the Medilog Darwin Analysis Software package and analysed using the Kubios premium 3.3 software package (University of Kuopio, Finland). The HRV analysis will focus on the following parameters: root mean square of successive differences adjacent normal NN intervals (RMSSD), percentage of successive normal sinus NN intervals differing more than 50 ms (pNN50) and power in

For peer review only

For peer review only - http://bmjopen.bmj.com/site/about/guidelines.xhtml

high (0.15–0.4 Hz) frequencies (HF). Further time and frequency domain parameters[124-125] will be analysed as well. In addition, we will also consider non-linear HRV parameters to study the non-linear dynamic properties that influence heart rate[67]. Due to the relatively long latency of the cardiac system[54], the HRV (as the fNIRS parameters) will be averaged over each block of the three cognitive tasks. Following the recommendations of Laborde et al.[50], we plan to record and analyse the HRV at three timepoints:

- at resting state before the cognitive tasks,
- during the cognitive tasks for each block and
- after the cognitive tasks at resting state in the recovery phase.

The baseline measurement before the cognitive tasks will serve as assessment of the vagal tone of the participants. The assessment of the vagal tone is important because “vagally mediated HRV may serve to index the functional capacity of a set of brain structures that support the effective and efficient performance of cognitive executive-function tasks including working memory and inhibitory control”[59]. The “phasic HRV” will be evaluated through comparing the HRV at rest with the values recorded during the cognitive tasks[50]. It shows how the ANS reacts and how the participant adapts to cognitive demands. Finally, the HRV will be recorded after the cognitive tasks at resting state (recovery phase). This experimental design allows for investigation of tonic HRV for each of the three measurement points (baseline, during and after the cognitive tasks). Furthermore, we can assess the phasic HRV because we will be able to detect the change between baseline and event (“reactivity”), the change between task and post-event (“recovery”) and the change between baseline and post-event[50].

For peer review only

2.6. Statistical analysis

The current study is designed to investigate biomarkers that can identify participants with an increased risk for cognitive deterioration. Thereto, several biomarkers will be evaluated, as possible candidates,

For peer review only - http://bmjopen.bmj.com/site/about/guidelines.xhtml

for an early identification of MCI. We are interested in the transition state between healthy aging and MCI. Consequently, our statistical analysis will include:

- analysis of the differences between HC and MCI with respect to cognitive performance and physiological parameters,
- correlational analysis between cognitive performance and physiological parameters and
- machine-learning based logistic regression approach for classification into HC and MCI.

To investigate effects of group, t test for normal distributed variables will be used, in other cases the Mann Whitney U test. Correction for multiple comparisons will be taken into account with False Discovery Rate[126]. To investigate the correlations, we will use Pearson or Spearman correlation analysis. To control for effects of age, gender, regular physical activity and education as confounders, partial correlation will be applied. Table 1 shows the measured physiological parameters of all three cognitive tasks. Concerning ECG analysis, we will also measure the HRV in resting state before (baseline) and after the cognitive tasks (recovery).

The main aim of our study is to develop EEG/fNIRS/HRV measures that discriminate among HC and MCI and show that this multimodal measuring approach is promising and accurate for identification of MCI. For that purpose, we will use and adapt the machine-learning algorithm already used for bimodal approaches[78-80, 82-83, 85]. In a first step, after pre-processing and artefact correction, parameters from the three modalities will be extracted and the most discriminating ones playing a role in the development of neurodegenerative diseases will be selected. Principal Component Analysis (PCA) can help to reduce parameters to a manageable amount by removing components with the highest variance[127]. Following parameter extraction, multimodal combining algorithm will be used for classification. To classify the signals, support vector machine (SVM) will be applied. SVM is one of the most commonly used supervised classifiers in the field of pattern recognition and has been widely adopted in many brain signal studies[73, 78-80, 82, 128]. SVM can define two or more classes by constructing an optimal hyperplane maximizing the margin of separation between the closest data

For peer review only

points belonging to different classes. SVM can be used in linear as well as in non-linear classification scenarios based on the kernel trick[129].

Another aim of the study is to compare the performance of uni-, bi- and multimodal systems. For that reason, classification should be performed separately using different kinds of parameter sets for comparison: EEG-only, fNIRS-only, HRV-only, bimodal (EEG + fNIRS, EEG + HRV, fNIRS + HRV) and a multimodal parameter set (EEG + fNIRS + HRV).

Table 1: Physiological parameters of each device in all three cognitive tasks.

Cognitive task	Physiological measurement device	Main parameters
Stroop (congruent and incongruent)	ECG	RMSSD, pNN50, HF (absolute and nu), non-linear parameters (SD1, SD2, ApEn, SampEn, DFA, D2, RPA, MSE)
	fNIRS	oxyHb, deoxyHb, totHb
	EEG	ERPs, PSD, connectivity and source localisation
N-back (0-, 1- and 2-back)	ECG	RMSSD, pNN50, HF (absolute and nu), non-linear parameters (SD1, SD2, ApEn, SampEn, DFA, D2, RPA, MSE)
	fNIRS	oxyHb, deoxyHb, totHb
	EEG	ERPs, PSD, connectivity and source localisation
VFT (semantic and category)	ECG	RMSSD, pNN50, HF (absolute and nu), non-linear parameters (SD1, SD2, ApEn, SampEn, DFA, D2, RPAn, MSE)
	fNIRS	oxyHb, deoxyHb, totHb
	EEG	PSD

For peer review only

538 RMSSD: root mean square of the successive differences between adjacent normal RR intervals; pNN50: NN50 divided by the total number of NN intervals; HF: high frequency power in absolute and normalized units (nu) [0.15–0.4 Hz]; SD1: in Poincaré plot, the standard deviation perpendicular to the line-of-identity; SD2: in Poincaré plot, the standard deviation along the line-of-identity; ApEn: approximate entropy; SampEn: sample entropy; DFA: detrended fluctuation analysis; D2: correlation dimension; RPAn: recurrence plot analysis; MSE: mutliscale entropy; oxyHb: oxygenated haemoglobin; deoxyHb: deoxygenated haemoglobin; totoHb: total haemoglobin; ERPs: event related potentials; PSD: power spectral density.

2.7. Data management and safety

All participants will be assigned a code independently from their group allocation ensuring their anonymity. In order to guarantee the security of all data, the personal information and the data of this study will be collected and handled exclusively by the involved researchers. All data and participants` information will be handled according to the institutional data management policy of the University of Magdeburg. The original documents will be kept by the main investigators of the study at the Otto von Guericke University Magdeburg and the German Center for Neurodegenerative Diseases.

2.8. Patient and public involvement

Subjects will be involved in the study as we will evaluate their acceptability for our measurement procedure. We will collect feedback on the study procedure from the participants. We will continue to work with stakeholders including the medical faculty and companies working in the field of neurodegeneration and dementia.

3. SUMMARY

Due to the demographic change and the increase of the individual life expectancy, the worldwide economic health care costs to treat individuals suffering from dementia will increase considerably. Currently, there is no treatment available which would allow to heal this neurological disease. Consequently, researchers now focus more on an early diagnosis of preclinical stages of AD (e.g., MCI) in order to initiate preventive actions timely. In this regard, neurophysiological signals could be

For peer review only - http://bmjopen.bmj.com/site/about/guidelines.xhtml

promising biomarkers of such preclinical AD stages because they are, unlike behavioural performance, deemed to be less affected by learning or practice effects and provide insights into possible compensatory processes being not readily observable at the behavioural level. Hence, physiological signals can be helpful to detect changes in cognitive performance more precisely[130]. In this context, it is assumed that the investigation of new neurophysiological biomarkers, which allows to identify MCI more easily and accurately, are necessary to better understand and monitor the disease progression[11].

In line with the previous mentioned assumptions, the aim of this study is threefold. At first, this investigation aims to determine the differences in neurophysiological responses of HC and MCI participants. The second aim is to investigate possible neurobehavioral relationships between measures of cognitive performance and neurophysiological responses. The third aim is to elucidate whether a multimodal measurement approach can help to identify individuals with MCI more accurately and reliable than a uni- or bimodal approach. Thereto, three complementary measuring modalities, namely fNIRS, EEG and ECG/HRV, will be used simultaneously to assess different neurophysiological responses which are associated with cognitive processes. To the best of our knowledge, this is the first study using these three modalities simultaneously in MCI patients and cognitive healthy controls. Given the explorative character of our study, our sample consists of 30 HC and 30 MCI patients whose neurophysiological signals will be recorded at a resting state and while performing three established cognitive tasks (Stroop, N-back and a verbal fluency test). We are aiming to detect certain neurophysiological parameters which are promising for an early identification of people which are at a higher risk of an overly age-related decline in cognitive performance (i.e., MCI detection).

In this regard, we hypothesize that the multimodal approach improves the classification accuracy between healthy controls and MCI patients as compared to a unimodal or a bimodal approach. If our hypothesis is verified, this study will pave the way for further research on multimodal measurement approaches for dementia research. Such upcoming research will use a larger sample size to examine

For peer review only - http://bmjopen.bmj.com/site/about/guidelines.xhtml

the non-invasive biomarkers characterizing an early detection of non-physiological decline in cognitive performance in more detail.

Ethics and dissemination

Ethics approval was obtained for the study from the Ethics Committee of the Otto von Guericke University (reference: 83/19), and informed consent for participation will be obtained from all participants. Data will be shared with the scientific community no more than 1 year following completion of study and data assembly.

For peer review only

Additional file

Additional file 1: spatial registration for fNIRS optodes for assignment of fNIRS measurement channels.

Abbreviations

AD: Alzheimer's disease; ANS: autonomic nervous system; ApEn: approximate entropy; BA: Broadmann atlas; BDNF: brain-derived neurotrophic factor; BMI: body mass index; CSF: cerebrospinal fluid; deoxyHb: deoxygenated haemoglobin; DFA: detrended fluctuation analysis; ECG: electrocardiography; EEG: electroencephalography; ERP: event-related potential; ES: effect size; fNIRS: functional near-infrared spectroscopy; HAROLD: Hemispheric Asymmetric Reduction in OLD adults; HC: healthy controls; HF: power in high (0.15–0.40 Hz) frequencies; HRV: heart rate variability; ICA: independent component analysis; IZ: inion; Loreta: low-resolution electromagnetic tomography analysis; LPA: left preauricular point; MCI: mild cognitive impairment; MM: mean mastoid; MMSE: Mini-Mental State Exam; MRI: magnetic resonance imaging; MSE: mutliscale entropy; NZ: nasion; oxyHb: oxygenated haemoglobin; PCA: Principal Component Analysis; PET: positron emission tomography; pNN50:

For peer review only - http://bmjopen.bmj.com/site/about/guidelines.xhtml

percentage of successive normal sinus NN intervals differing more than 50 ms; PSD: power spectral density; RMSSD: root mean square of the successive differences between adjacent normal NN intervals; RPA: right preauricular point; RPAn: recurrence plot analysis; SampEn: sample entropy; SD: standard deviation; SVM: support vector machine; VFT: verbal fluency test

Authors' contributions

BG, FH, MD and TAG designed the study protocol. AH and NGM conceptualised and secured funding for the research study. BG, FH and MD contributed to the application to the local Ethics Committee. BG wrote the first draft of this publication with contributions from FH (fNIRS) and TAG (EEG). FH, MD, SD, NGM, IB and AH revised the initial draft. FH, TAG and SD settled the trial materials. IB, NGM and AH were responsible for the final study protocol. BG, FH, MD and TAG will participate in data collection. All authors read and approved the final manuscript.

Competing interests statement

The authors declare that they have no competing interests.

Funding statement

This work is supported by the joint project "MyFit" funded by the European Regional Development Fund (grant number: ZS/2018/08/94206). The funder has no role in the design or conduct of the study.

For peer review only

For peer review only - http://bmjopen.bmj.com/site/about/guidelines.xhtml

References

1 2020 Alzheimer's disease facts and figures. Alzheimers Dement 2020.

2 WHO. Risk reduction of cognitive decline and dementia: WHO guidelines. Geneva: World Health Organization; 2019.

3 Dilling H, editor. Internationale Klassifikation psychischer Störungen: ICD-10 Kapitel V (F); klinisch-diagnostische Leitlinien. 6., vollst. überarb. Aufl. unter Berücksichtigung der Änderungen entsprechend ICD-10-GM 2004/2008. Bern: Huber; 2008. Available from: URL: http://d-nb.info/988424061/04.

4 Leicht H, Heinrich S, Heider D, et al. Net costs of dementia by disease stage. *Acta Psychiatr Scand* 2011;124(5):384–95.

5 Wallesch C-W, Förstl H, Bartels C. Demenzen. 3., unveränderte Auflage. Stuttgart, New York: Georg Thieme Verlag; 2017. (RRN, Referenz-Reihe Neurologie).

6 Wimo A, Guerchet M, Ali G-C, et al. The worldwide costs of dementia 2015 and comparisons with 2010. *Alzheimers Dement* 2017;13(1):1–7.

7 Etgen T, Sander D, Bickel H, et al. Mild cognitive impairment and dementia: the importance of modifiable risk factors. *Dtsch Arztebl Int* 2011;108(44):743–50.

8 Petersen RC, Smith GE, Waring SC, et al. Mild Cognitive Impairment: Clinical Characterization and Outcome. *Arch Neurol* 1999;56(3):303–8. Available from: URL: https://jamanetwork.com/journals/jamaneurology/fullarticle/774828.

9 Petersen RC. Mild cognitive impairment as a diagnostic entity. *J Intern Med* 2004;256(3):183–94.

10 Dubois B, Hampel H, Feldman HH, et al. Preclinical Alzheimer's disease: Definition, natural history, and diagnostic criteria. *Alzheimers Dement* 2016;12(3):292–323.

11 Jongsiriyanyong S, Limpawattana P. Mild Cognitive Impairment in Clinical Practice: A Review Article. *Am J Alzheimers Dis Other Demen* 2018;33(8):500–7.

For peer review only - http://bmjopen.bmj.com/site/about/guidelines.xhtml

12 Kivipelto M, Mangialasche F, Ngandu T. Lifestyle interventions to prevent cognitive impairment, dementia and Alzheimer disease. *Nat Rev Neurol* 2018;14(11):653–66.

13 Yiannopoulou KG, Papageorgiou SG. Current and Future Treatments in Alzheimer Disease: An Update. *J Cent Nerv Syst Dis* 2020;12:1179573520907397.

14 Atri A. The Alzheimer's Disease Clinical Spectrum: Diagnosis and Management. *Med Clin North Am* 2019;103(2):263–93.

15 Annavarapu RN, Kathi S, Vadla VK. Non-invasive imaging modalities to study neurodegenerative diseases of aging brain. *J Chem Neuroanat* 2019;95:54–69.

16 Pinti P, Tachtsidis I, Hamilton A, Hirsch J, et al. The present and future use of functional near-infrared spectroscopy (fNIRS) for cognitive neuroscience. *Annals of the New York Academy of Sciences* 2020;1464;5–29.

17 Herold F, Wiegel P, Scholkmann F, et al. Applications of Functional Near-Infrared Spectroscopy (fNIRS) Neuroimaging in Exercise–Cognition Science: A Systematic, Methodology-Focused Review. *J Clin Med* 2018;7(12).

18 Agbangla NF, Audiffren M, Albinet CT. Use of near-infrared spectroscopy in the investigation of brain activation during cognitive aging: A systematic review of an emerging area of research. *Ageing Research Reviews* 2017;38:52–66.

19 Fantini S, Ruesch A, Kainerstorfer JM. Noninvasive Optical Studies of the Brain. In: Shi L, Alfano RR, editors. Neurophotonics and biomedical spectroscopy. Amsterdam: Elsevier; 2018:25–52.

20 Scholkmann F, Kleiser S, Metz AJ, et al. A review on continuous wave functional near-infrared spectroscopy and imaging instrumentation and methodology. *NeuroImage* 2014;85;1;6–27.

21 Chou PH, Lan TH. The role of near-infrared spectroscopy in Alzheimer's disease. *Journal of Clinical Gerontology and Geriatrics* 2013;4(2):33–6.

For peer review only

22 Obrig H. NIRS in clinical neurology - a 'promising' tool? *NeuroImage* 2014;85;1:535–46.

23 Yeung MK, Chan AS. Functional near-infrared spectroscopy reveals decreased resting oxygenation levels and task-related oxygenation changes in mild cognitive impairment and dementia: A systematic review. *Journal of Psychiatric Research* 2020;124:58–76.

24 Katzorke A, Zeller JBM, Müller LD, et al. Decreased hemodynamic response in inferior frontotemporal regions in elderly with mild cognitive impairment. *Psychiatry Res Neuroimaging* 2018;274:11–8.

25 Yeung MK, Sze SL, Woo J, et al. Altered Frontal Lateralization Underlies the Category Fluency Deficits in Older Adults with Mild Cognitive Impairment: A Near-Infrared Spectroscopy Study. *Front Aging Neurosci* 2016;8:59.

26 Vermeij A, van Beek AHEA, Olde Rikkert MGM, et al. Effects of aging on cerebral oxygenation during working-memory performance: a functional near-infrared spectroscopy study. *PLoS ONE* 2012;7(9):e46210.

27 Nguyen T, Kim M, Gwak J, et al. Investigation of brain functional connectivity in patients with mild cognitive impairment: A functional near-infrared spectroscopy (fNIRS) study. *J Biophotonics* 2019;12(9):e201800298.

28 Niu H-J, Li X, Chen Y-J, et al. Reduced frontal activation during a working memory task in mild cognitive impairment: a non-invasive near-infrared spectroscopy study. *CNS Neurosci Ther* 2013;19(2):125–31.

29 Yang D, Hong K-S, Yoo S-H, et al. Evaluation of Neural Degeneration Biomarkers in the Prefrontal Cortex for Early Identification of Patients With Mild Cognitive Impairment: An fNIRS Study. *Front Hum Neurosci* 2019;13:317.

For peer review only

For peer review only - http://bmjopen.bmj.com/site/about/guidelines.xhtml

30 Yap KH, Ung WC, Ebenezer EGM, et al. Visualizing Hyperactivation in Neurodegeneration Based on Prefrontal Oxygenation: A Comparative Study of Mild Alzheimer's Disease, Mild Cognitive Impairment, and Healthy Controls. *Front Aging Neurosci* 2017;9:287.

31 Yoo S-H, Hong K-S. Hemodynamics Analysis of Patients With Mild Cognitive Impairment During Working Memory Tasks. Conf Proc IEEE Eng Med Biol Soc 2019;4470–3.

32 Cabeza R. Hemispheric asymmetry reduction in older adults: The HAROLD model. *Psychol Aging* 2002;17(1):85–100.

33 Michel CM, Murray MM. Towards the utilization of EEG as a brain imaging tool. *NeuroImage* 2012;61(2):371–85.

34 Hampel H, Lista S, Teipel SJ, et al. Perspective on future role of biological markers in clinical therapy trials of Alzheimer's disease: a long-range point of view beyond 2020. *Biochem Pharmacol* 2014;88(4):426–49.

35 Haroutunian V, Hoffman LB, Been MS. Is there a neuropathology difference between mild cognitive impairment and dementia? *Dialogues Clin Neurosci* 2009;11(2):171–9.

36 Jelic V, Kowalski J. Evidence-based evaluation of diagnostic accuracy of resting EEG in dementia and mild cognitive impairment. *Clin EEG Neurosci* 2009;40(2):129–42.

37 Scheff SW, Price DA, Schmitt FA, et al. Hippocampal synaptic loss in early Alzheimer's disease and mild cognitive impairment. *Neurobiol Aging* 2006;27(10):1372–84.

38 Al-Qazzaz NK, Ali SHBM, Ahmad SA, et al. Role of EEG as biomarker in the early detection and classification of dementia. *Scientific World Journal* 2014;2014:906038.

39 Choi J, Ku B, You YG, et al. Resting-state prefrontal EEG biomarkers in correlation with MMSE scores in elderly individuals. *Scientific Reports* 2019;9(1):10468.

For peer review only

For peer review only - http://bmjopen.bmj.com/site/about/guidelines.xhtml

40 Gouw AA, Stam CJ. Electroencephalography in the differential diagnosis of dementia. Epileptologie 2016; 33:173. Available from: URL: https://research.vumc.nl/en/publications/electroencephalography-in-the-differential-diagnosis-of-dementia.

41 Horvath A, Szucs A, Csukly G, et al. EEG and ERP biomarkers of Alzheimer's disease: a critical review. *Front Biosci (Landmark Ed)* 2018;23:183–220.

42 Yang S, Bornot JMS, Wong-Lin K, et al. M/EEG-Based Bio-Markers to Predict the MCI and Alzheimer's Disease: A Review From the ML Perspective. *IEEE Trans Biomed Eng* 2019;66(10):2924–35.

43 Sur S, Sinha VK. Event-related potential: An overview. *Ind Psychiatry J* 2009;18(1):70–3.

44 Sakkalis V. Review of advanced techniques for the estimation of brain connectivity measured with EEG/MEG. *Computers in Biology and Medicine* 2011;41(12):1110–7.

45 Sarmukadam K, Sharpley CF, Bitsika V, et al. A review of the use of EEG connectivity to measure the neurological characteristics of the sensory features in young people with autism. *Rev Neurosci* 2019;30(5):497–510.

46 Schoffelen J-M, Gross J. Source connectivity analysis with MEG and EEG. *Hum Brain Mapp* 2009;30(6):1857–65.

47 Schapkin SA, Raggatz J, Hillmert M, et al. EEG correlates of cognitive load in a multiple choice reaction task. *Acta Neurobiol Exp (Wars)* 2020;80(1):76–89.

48 Schomer DL, Lopes da Silva FH, editors. Niedermeyer's electroencephalography: Basic principles, clinical applications, and related fields. 7 ed. New York: Oxford University Press; 2017.

49 Laborde S, Mosley E, Thayer JF. Heart rate variability and cardiac vagal tone in psychophysiological research - Recommendations for experiment planning, data analysis, and data reporting. *Frontiers in Psychology* 2017;8(FEB).

For peer review only

For peer review only - http://bmjopen.bmj.com/site/about/guidelines.xhtml

50 McCraty R, Shaffer F. Heart rate variability: New perspectives on physiological mechanisms, assessment of self-regulatory capacity, and health risk. *Global Advances In Health and Medicine* 2015;4(1):46–61.

51 Billman GE. The LF/HF ratio does not accurately measure cardiac sympatho-vagal balance. *Frontiers in Physiology* 2013;4(FEB).

52 Draghici AE, Taylor JA. The physiological basis and measurement of heart rate variability in humans. *J Physiol Anthropol* 2016;35(1):22.

53 Shaffer F, McCraty R, Zerr CL. A healthy heart is not a metronome: an integrative review of the heart's anatomy and heart rate variability. *Frontiers in Psychology* 2014;5.

54 Fatisson J, Oswald V, Lalonde F. Influence diagram of physiological and environmental factors affecting heart rate variability: An extended literature overview. *Heart International* 2016;11(1):e32-e40.

55 Sammito S, Böckelmann I. Factors influencing heart rate variability. *ICFJ* 2016;6.

56 Thayer JF, Yamamoto SS, Brosschot JF. The relationship of autonomic imbalance, heart rate variability and cardiovascular disease risk factors. *International Journal of Cardiology* 2010;141(2):122–31.

57 Thayer JF, Lane RD. A model of neurovisceral integration in emotion regulation and dysregulation. *Journal of Affective Disorders* 2000;61(3):201–16.

58 Thayer JF, Hansen AL, Saus-Rose E, et al. Heart rate variability, prefrontal neural function, and cognitive performance: The neurovisceral integration perspective on self-regulation, adaptation, and health. *Annals of Behavioral Medicine* 2009;37(2):141–53.

59 Thayer JF, Lane RD. Claude Bernard and the heart-brain connection: Further elaboration of a model of neurovisceral integration. *Neuroscience and Biobehavioral Reviews* 2009;33(2):81–8.

For peer review only

For peer review only - http://bmjopen.bmj.com/site/about/guidelines.xhtml

60 Forte G, Favieri F, Casagrande M. Heart Rate Variability and Cognitive Function: A Systematic Review. *Front Neurosci* 2019;13:710.

61 Eggenberger P, Annaheim S, Kündig KA, et al. Heart Rate Variability Mainly Relates to Cognitive Executive Functions and Improves Through Exergame Training in Older Adults: A Secondary Analysis of a 6-Month Randomized Controlled Trial. *Front Aging Neurosci* 2020;12:197.

62 Grässler B, Hökelmann A, Cabral RH. Resting heart rate variability as a possible marker of cognitive decline. *Kinesiology (Zagreb, Online)* 2020;52(1):72–84.

63 Zeki Al Hazzouri A, Haan MN, Deng Y, et al. Reduced heart rate variability is associated with worse cognitive performance in elderly Mexican Americans. *Hypertension* 2014;63(1):181–7.

64 Kim DH, Lipsitz LA, Ferrucci L, et al. Association between reduced heart rate variability and cognitive impairment in older disabled women in the community: Women's Health and Aging Study I. *Journal of the American Geriatrics Society* 2006;54(11):1751–7.

65 da Silva VP, Ramalho Oliveira BR, Tavares Mello RG, et al. Heart Rate Variability Indexes in Dementia: A Systematic Review with a Quantitative Analysis. *Curr Alzheimer Res* 2018;15(1):80–8.

66 Delliaux S, Delaforge A, Deharo J-C, et al. Mental Workload Alters Heart Rate Variability, Lowering Non-linear Dynamics. *Front Physiol* 2019;10:565.

67 Mukherjee S, Yadav R, Yung I, et al. Sensitivity to mental effort and test-retest reliability of heart rate variability measures in healthy seniors. *Clin Neurophysiol* 2011;122(10):2059–66.

68 Sassi R, Cerutti S, Lombardi F, et al. Advances in heart rate variability signal analysis: Joint position statement by the e-Cardiology ESC Working Group and the European Heart Rhythm Association co-endorsed by the Asia Pacific Heart Rhythm Society. *Europace* 2015;17(9):1341–53.

69 Young H, Benton D. We should be using nonlinear indices when relating heart-rate dynamics to cognition and mood. *Scientific Reports* 2015;5:16619.

For peer review only

For peer review only - http://bmjopen.bmj.com/site/about/guidelines.xhtml

70 Fernandes de Godoy M. Nonlinear Analysis of Heart Rate Variability: A Comprehensive Review. *Journal of Cardiology and Therapy* 2016;3(3):528–33.

71 Bari V, Calcagnile P, Molteni E, et al. From neurovascular coupling to neurovascular cascade: a study on neural, autonomic and vascular transients in attention. *Physiol Meas* 2012;33(8):1379–97.

72 Ha U, Lee Y, Kim H, et al. A Wearable EEG-HEG-HRV Multimodal System With Simultaneous Monitoring of tES for Mental Health Management. *IEEE Trans Biomed Circuits Syst* 2015;9(6):758–66.

73 Chiarelli AM, Zappasodi F, Di Pompeo F, et al. Simultaneous functional near-infrared spectroscopy and electroencephalography for monitoring of human brain activity and oxygenation: a review. *Neurophotonics* 2017;4(4):41411.

74 Alba G, Vila J, Rey B, et al. The Relationship Between Heart Rate Variability and Electroencephalography Functional Connectivity Variability Is Associated With Cognitive Flexibility. *Front Hum Neurosci* 2019;13:64.

75 Hillmert M, Bergmüller A, Minow A, et al. Psychophysiologische Beanspruchungskorrelate während kognitiver Belastung. *Zbl Arbeitsmed* 2020:1–15. Available from: URL: https://link.springer.com/article/10.1007/s40664-020-00384-9.

76 Brugnera A, Adorni R, Compare A, et al. Cortical and Autonomic Patterns of Emotion Experiencing During a Recall Task. *Journal of Psychophysiology* 2018;32(2):53–63.

77 Aghajani H, Garbey M, Omurtag A. Measuring Mental Workload with EEG+fNIRS. *Front Hum Neurosci* 2017;11:359.

78 Ahn JW, Ku Y, Kim HC. A Novel Wearable EEG and ECG Recording System for Stress Assessment. *Sensors (Basel)* 2019;19(9).

79 Al-Shargie F, Tang TB, Kiguchi M. Stress Assessment Based on Decision Fusion of EEG and fNIRS Signals. *IEEE Access* 2017;5:19889–96.

For peer review only

80 Li R, Nguyen T, Potter T, et al. Dynamic cortical connectivity alterations associated with Alzheimer's disease: An EEG and fNIRS integration study. *Neuroimage Clin* 2019;21:101622.

81 Li R, Potter T, Huang W, et al. Enhancing Performance of a Hybrid EEG-fNIRS System Using Channel Selection and Early Temporal Features. *Front Hum Neurosci* 2017;11:462.

82 Liu Y, Ayaz H, Shewokis PA. Multisubject "Learning" for Mental Workload Classification Using Concurrent EEG, fNIRS, and Physiological Measures. *Front Hum Neurosci* 2017;11:389.

83 Morioka H, Kanemura A, Morimoto S, et al. Decoding spatial attention by using cortical currents estimated from electroencephalography with near-infrared spectroscopy prior information. *NeuroImage* 2014;90:128–39.

84 Shin J, Kwon J, Im C-H. A Ternary Hybrid EEG-NIRS Brain-Computer Interface for the Classification of Brain Activation Patterns during Mental Arithmetic, Motor Imagery, and Idle State. Front *Neuroinform* 2018;12:5.

85 Nicolini P, Ciulla MM, Malfatto G, et al. Autonomic dysfunction in mild cognitive impairment: Evidence from power spectral analysis of heart rate variability in a cross-sectional case-control study. *PLoS ONE* 2014;9(5).

86 Lai C-L, Lin R-T, Liou L-M, et al. The role of event-related potentials in cognitive decline in Alzheimer's disease. *Clin Neurophysiol* 2010, 121(2):194–199.

87 Faul F, Erdfelder E, Lang A-G, et al. G*Power 3: a flexible statistical power analysis program for the social, behavioral, and biomedical sciences. *Behav Res Methods* 2007;39(2):175–91.

88 Ahn S, Nguyen T, Jang H, et al. Exploring Neuro-Physiological Correlates of Drivers' Mental Fatigue Caused by Sleep Deprivation Using Simultaneous EEG, ECG, and fNIRS Data. *Front Hum Neurosci* 2016;10:219.

For peer review only

For peer review only - http://bmjopen.bmj.com/site/about/guidelines.xhtml

89 Sperling RA, Aisen PS, Beckett LA, et al. Toward defining the preclinical stages of Alzheimer's disease: Recommendations from the National Institute on Aging-Alzheimer's Association workgroups on diagnostic guidelines for Alzheimer's disease. *Alzheimers Dement* 2011;7(3):280–92.

90 Morris JC, Mohs RC, Rogers H, et al. Consortium to establish a registry for Alzheimer's disease (CERAD) clinical and neuropsychological assessment of Alzheimer's disease. *Psychopharmacol Bull* 1988;24(4):641–52.

91 Barth S, Schönknecht P, Pantel J, et al. Neuropsychologische Profile in der Demenzdiagnostik: Eine Untersuchung mit der CERAD-NP-Testbatterie. *Fortschr Neurol Psychiatr* 2005;73(10):568–76.

92 Huy C, Schneider S. Instrument für die Erfassung der physischen Aktivität bei Personen im mittleren und höheren Erwachsenenalter. Entwicklung, Prüfung und Anwendung des "German-PAQ-50+". *Z Gerontol Geriatr* 2008;41(3):209-216.

93 Ware JE. Sf-36 health survey update. *Spine* 2000;25(24):3130–3139.

94 Buysse DJ, Reynolds CF, Monk TH, et al. The Pittsburgh sleep quality index: A new instrument for psychiatric practice and research. *Psychiatry Res* 1989;28(2):193–213.

95 Haftenberger M, Heuer T, Heidemann C, et al. Relative validation of a food frequency questionnaire for national health and nutrition monitoring. *Nutr J* 2010:9(36).

96 Fahrenberg J, Schönpflug W. Methoden der Aktivierungsforschung. Bern, Stuttgart, Wien: Hans Huber; 1969. (Methoden der Aktivierungsforschung; vol 6).

97 Hancock PA, editor. Human mental workload. Amsterdam: North-Holland; 1988. (Advances in psychology; vol 52).

98 Longo L. On the Reliability, Validity and Sensitivity of Three Mental Workload Assessment Techniques for the Evaluation of Instructional Designs: A Case Study in a Third-level Course. In: Proceedings of the 10th International Conference on Computer Supported Education. *SCITEPRESS - Science and Technology Publications* 2018;166–78.

For peer review only

For peer review only - http://bmjopen.bmj.com/site/about/guidelines.xhtml

99 Stroop JR. Studies of interference in serial verbal reactions. *Journal of Experimental Psychology* 1935;18(6):643–62.

100 Bélanger S, Belleville S, Gauthier S. Inhibition impairments in Alzheimer's disease, mild cognitive impairment and healthy aging: effect of congruency proportion in a Stroop task. *Neuropsychologia* 2010;48(2):581–90.

101 Sun J, Sun B, Zhang L, et al. Correlation between hemodynamic and electrophysiological signals dissociates neural correlates of conflict detection and resolution in a Stroop task: a simultaneous near-infrared spectroscopy and event-related potential study. *J Biomed Opt* 2013;18(9):96014.

102 Mansouri C, Kashou NH. Spatial sensitivity of near-infrared spectroscopic brain imaging based on three-dimensional Monte Carlo modeling. *Conf Proc IEEE Eng Med Biol Soc* 2009;1457–60.

103 Badzakova-Trajkov G, Barnett KJ, Waldie KE, et al. An ERP investigation of the Stroop task: the role of the cingulate in attentional allocation and conflict resolution. *Brain Res* 2009;1253:139–48.

104 Egner T, Hirsch J. The neural correlates and functional integration of cognitive control in a Stroop task. *NeuroImage* 2005;24(2):539–47.

105 Compton RJ, Huber E, Levinson AR, et al. Is "conflict adaptation" driven by conflict? Behavioral and EEG evidence for the underappreciated role of congruent trials. *Psychophysiology* 2012;49(5):583–9.

106 Satish P, Muralikrishnan K, Balasubramanian K, et al. Heart rate variability changes during stroop color and word test among genders. *Indian J Physiol Pharmacol* 2015;59(1):9–15.

107 Baumert A, Buchholz N, Zinkernagel A, et al. Causal underpinnings of working memory and Stroop interference control: Testing the effects of anodal and cathodal tDCS over the left DLPFC. *Cogn Affect Behav Neurosci* 2020;20(1):34–48.

108 Kirchner WK. Age differences in short-term retention of rapidly changing information. *Journal of Experimental Psychology* 1958;55(4):352–8.

For peer review only

For peer review only - http://bmjopen.bmj.com/site/about/guidelines.xhtml

109 Herrmann MJ, Langer JBM, Jacob C, et al. Reduced prefrontal oxygenation in Alzheimer disease during verbal fluency tasks. *Am J Geriatr Psychiatry* 2008;16(2):125–35.

110 Aschenbrenner S, Tucha O, Lange KW. Regensburger Wortflüssigkeits-Test: RWT; Handanweisung. Göttingen: Hogrefe Verl. für Psychologie; 2000.

111 Heinzel S, Metzger FG, Ehlis AC, et al. Age and vascular burden determinants of cortical hemodynamics underlying verbal fluency. *PLoS ONE* 2015;10(9).

112 Jurcak V, Tsuzuki D, Dan I. 10/20, 10/10, and 10/5 systems revisited: their validity as relative head-surface-based positioning systems. *NeuroImage* 2007;34(4):1600–11.

113 Zimeo Morais GA, Balardin JB, Sato JR. fNIRS Optodes' Location Decider (fOLD): a toolbox for probe arrangement guided by brain regions-of-interest. *Scientific Reports* 2018;8(1):3341.

114 Rorden C, Brett M. Stereotaxic display of brain lesions. *Behav Neurol* 2000;12(4):191–200.

115 Tachtsidis I, Scholkmann F. False positives and false negatives in functional near-infrared spectroscopy: issues, challenges, and the way forward. *Neurophotonics* 2016;3(3):30401.

116 Pinti P, Scholkmann F, Hamilton A, et al. Current Status and Issues Regarding Pre-processing of fNIRS Neuroimaging Data: An Investigation of Diverse Signal Filtering Methods Within a General Linear Model Framework. *Front Hum Neurosci* 2018;12:505.

117 Huppert TJ, Diamond SG, Franceschini MA, et al. HomER: a review of time-series analysis methods for near-infrared spectroscopy of the brain. *Appl Opt* 2009;48(10):D280-98.

118 Brigadoi S, Ceccherini L, Cutini S, et al. Motion artifacts in functional near-infrared spectroscopy: a comparison of motion correction techniques applied to real cognitive data. *NeuroImage* 2014;85(0 1).

119 Scholkmann F, Wolf M. General equation for the differential pathlength factor of the frontal human head depending on wavelength and age. *Journal of Biomedical Optics* 2013;18(10):105004.

For peer review only

For peer review only - http://bmjopen.bmj.com/site/about/guidelines.xhtml

120 Jahani S, Fantana AL, Harper D, et al. fNIRS can robustly measure brain activity during memory encoding and retrieval in healthy subjects. *Scientific Reports* 2017;7(1):9533.

121 Lühmann A von, Li X, Müller K-R, et al. Improved physiological noise regression in fNIRS: A multimodal extension of the General Linear Model using temporally embedded Canonical Correlation Analysis. *NeuroImage* 2020;208:116472.

122 Kropotov ID. Quantitative EEG, event-related potentials and neurotherapy. 1st ed. Amsterdam, Boston, London: Elsevier/Academic; 2009. Available from: URL: http://site.ebrary.com/lib/alltitles/docDetail.action?docID=10254769.

123 Lei X, Liao K. Understanding the Influences of EEG Reference: A Large-Scale Brain Network Perspective. *Front Neurosci* 2017;11:205.

124 Malik M. Heart rate variability: Standards of measurement, physiological interpretation, and clinical use. *Circulation* 1996;93(5):1043–65.

125 Sammito S, Thielmann B, Seibt R, et al. Guideline for the application of heart rate and heart rate variability in occupational medicine and occupational science. *ASUI* 2015;2015(06).

126 Chen S-Y, Feng Z, Yi X. A general introduction to adjustment for multiple comparisons. *J Thorac Dis* 2017 9(6):1725–9.

127 Dahne S, Bieszmann F, Samek W, et al. Multivariate Machine Learning Methods for Fusing Multimodal Functional Neuroimaging Data. Proc. *IEEE* 2015;103(9):1507–30.

128 Hong K-S, Khan MJ, Hong MJ. Feature Extraction and Classification Methods for Hybrid fNIRS-EEG Brain-Computer Interfaces. *Front Hum Neurosci* 2018;12:246.

129 Murty MN, Raghava R. Support Vector Machines and Perceptrons. Cham: Springer International Publishing; 2016.

For peer review only

For peer review only - http://bmjopen.bmj.com/site/about/guidelines.xhtml

130 Ranchet M, Morgan JC, Akinwuntan AE, et al. Cognitive workload across the spectrum of cognitive impairments: A systematic review of physiological measures. *Neuroscience and Biobehavioral Reviews* 2017;80:516–37.

Legends of figures

Figure 1: C: congruent condition; In: incongruent condition; M: mixed condition; s: seconds.

Figure 2: 0: 0-back; 1: 1-back; 2: 2-back; s: seconds.

Figure 3: s: seconds.

Figure 4: IZ: inion; LPA: left preauricular point; NZ: nasion; RPA: right preauricular point.

Figure 5: ICA: independent component analysis. Loreta: Low-resolution electromagnetic tomography analysis.

For peer review only

For peer review only - http://bmjopen.bmj.com/site/about/guidelines.xhtml

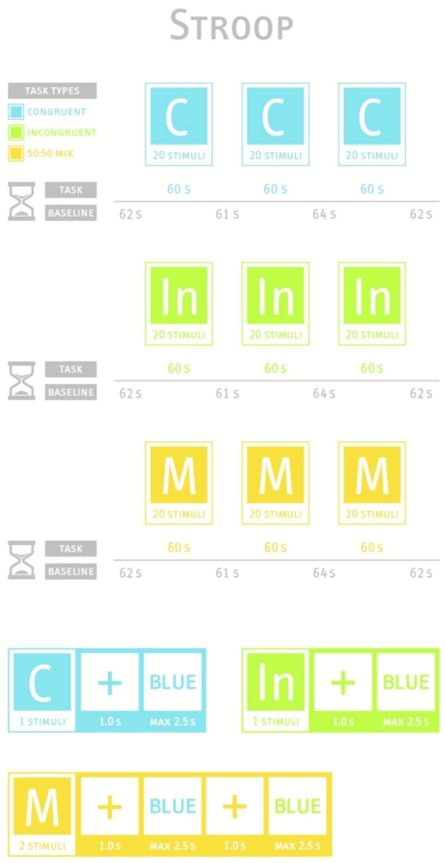

Paradigm of the Stroop test.
C: congruent condition; In: incongruent condition; M: mixed condition; s: seconds.

968x1104mm (96 x 96 DPI)

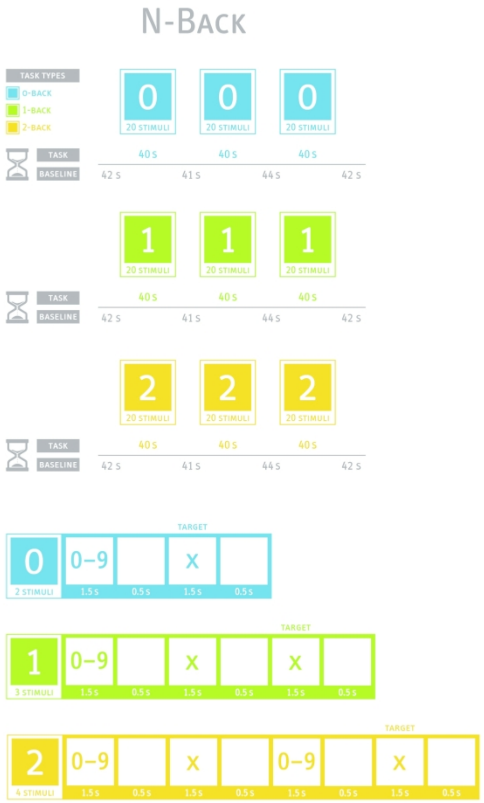

Paradigm of the N-back test.
0-back; 1: 1-back; 2: 2-back; s: seconds.

309x450mm (300 x 300 DPI)

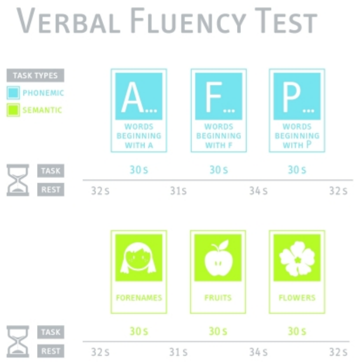

Paradigm of the verbal fluency test.
s: seconds.

309x450mm (300 x 300 DPI)

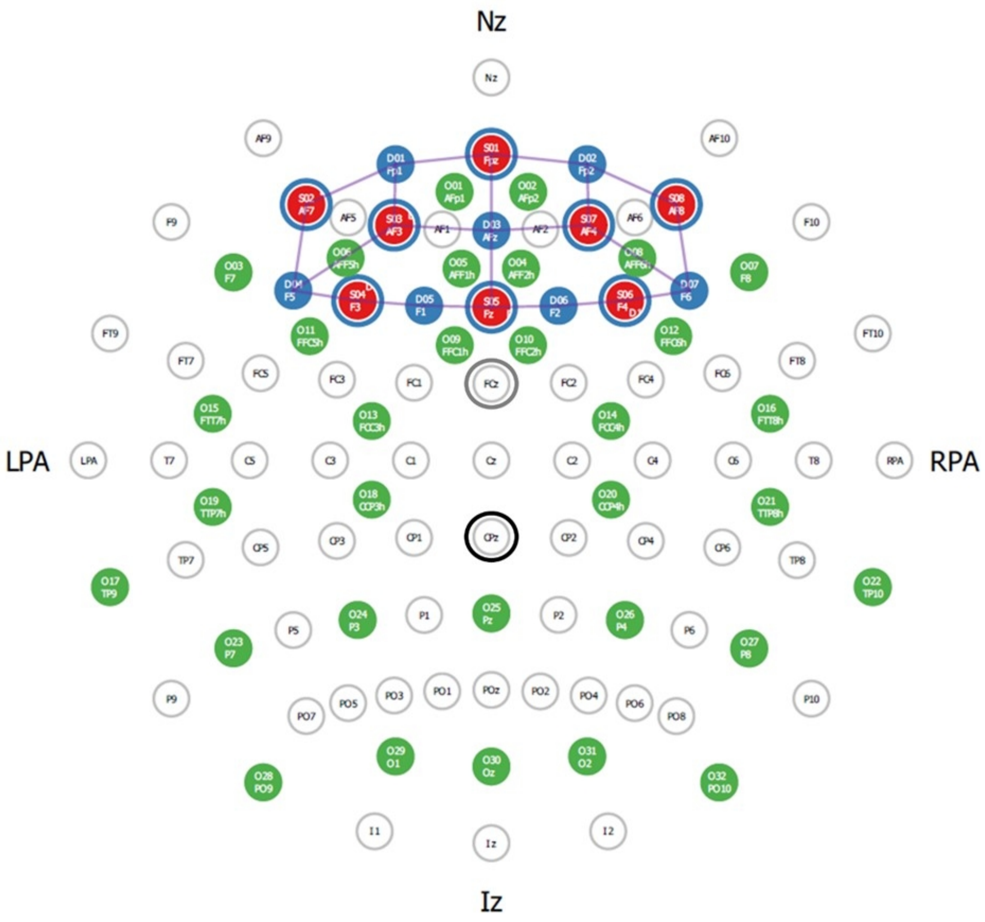

Schematic illustration of the fNIRS optode positions and EEG electrode positions. The ‘red dots’ represent sources whereas the ‘blue dots’ represent detectors. A ‘blue circle’ around a detector indicates the position of a short-separation channel. The measurements channels, which are displayed in ‘purple’, are combinations of distinct sources and detectors. The ‘green dots’ illustrate the position of the EEG electrode. The reference electrode and ground electrode are indicated by a ‘grey circle’ and ‘black circle’ respectively. IZ: inion; LPA: left preauricular point; NZ: nasion; RPA: right preauricular point.

159x152mm (150 x 150 DPI)

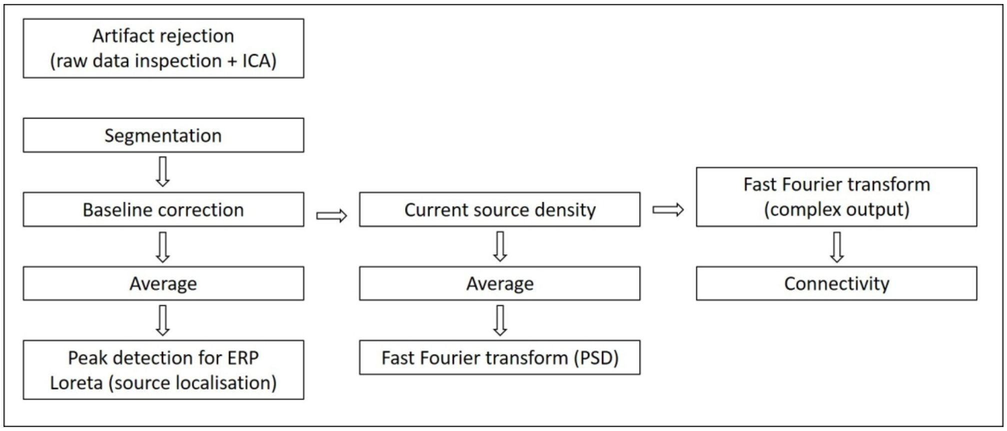

EEG data post-processing pipeline after re-referencing and filtering.
ICA: independent component analysis. Loreta: Low-resolution electromagnetic tomography analysis.

305x129mm (150 x 150 DPI)

Position	Source	Detector	X (mm)	Y (mm)	Z (mm)	Landmark	Specificity (%)	Distance (mm)
F4-F2	F4	F2	30	40	41	9 - Dorsolateral prefrontal cortex	68,36664315	30
						46 - Dorsolateral prefrontal cortex	22,39597886	
						8 - Includes Frontal eye fields	5,568764573	
F4-F6	F4	F6	46	38	24	45 - pars triangularis Broca's area	70,67253504	30
						46 - Dorsolateral prefrontal cortex	23,4516285	
AF8-F6	AF8	F6	48	46	5	45 - pars triangularis Broca's area	43,87699256	33
						46 - Dorsolateral prefrontal cortex	43,17605645	
						47 - Inferior prefrontal gyrus	5,07367444	
AF8-Fp2	AF8	Fp2	34	59	-2	10 - Frontopolar area	31,0841122	30
						11 - Orbitofrontal area	30,472822	
						46 - Dorsolateral prefrontal cortex	20,4016318	
AF4-F2	AF4	F2	22	52	33	9 - Dorsolateral prefrontal cortex	51,5248078	44
						46 - Dorsolateral prefrontal cortex	26,41541438	
						10 - Frontopolar area	18,40538603	
AF4-F6	AF4	F6	40	50	16	46 - Dorsolateral prefrontal cortex	47,36091434	45
						45 - pars triangularis Broca's area	30,60393436	
						10 - Frontopolar area	18,97467131	
AF4-Fp2	AF4	Fp2	25	63	9	10 - Frontopolar area	68,78124608	28
						11 - Orbitofrontal area	21,56708753	

For peer review only

						46 - Dorsolateral prefrontal cortex	6,609548215	
AF4-AFz	AF4	AFz	13	61	24	10 - Frontopolar area	72,46582734	36
						9 - Dorsolateral prefrontal cortex	16,97337784	
						46 - Dorsolateral prefrontal cortex	7,97503285	
Fpz-Fp2	Fpz	Fp2	13	67	0	10 - Frontopolar area	54,45817975	31
						11 - Orbitofrontal area	44,84702617	
Fpz-Afz	Fpz	AFz	1	64	14	10 - Frontopolar area	87,47712753	40
						9 - Dorsolateral prefrontal cortex	5,080149043	
Fpz-Fp1	Fpz	Fp1	-12	67	0	10 - Frontopolar area	54,49683736	30
						11 - Orbitofrontal area	44,89777168	
AF3-AFz	AF3	AFz	-12	62	23	10 - Frontopolar area	75,76225222	36
						9 - Dorsolateral prefrontal cortex	14,5484687	
						46 - Dorsolateral prefrontal cortex	8,053792322	
AF3-Fp1	AF3	Fp1	-24	63	9	10 - Frontopolar area	69,63399373	27
						11 - Orbitofrontal area	20,11557888	
						46 - Dorsolateral prefrontal cortex	8,793079404	
AF3-F5	AF3	F5	-39	50	17	46 - Dorsolateral prefrontal cortex	49,33992409	44
						45 - pars triangularis Broca's area	32,11689876	
						10 - Frontopolar area	16,03613047	

For peer review only

AF3-F1	AF3	F1	-23	52	32	9 - Dorsolateral prefrontal cortex	48,43964622	44
						46 - Dorsolateral prefrontal cortex	32,04248075	
						10 - Frontopolar area	16,88767141	
AF7-Fp1	AF7	Fp1	-33	59	-2	11 - Orbitofrontal area	32,71174196	30
						46 - Dorsolateral prefrontal cortex	25,27140585	
						10 - Frontopolar area	25,12699295	
						47 - Inferior prefrontal gyrus	13,10460546	
AF7-F5	AF7	F5	-47	46	6	45 - pars triangularis Broca's area	48,7862379	33
						46 - Dorsolateral prefrontal cortex	43,20372761	
F3-F5	F3	F5	-46	39	26	45 - pars triangularis Broca's area	72,56408005	29
						46 - Dorsolateral prefrontal cortex	21,83837451	
F3-F1	F3	F1	-31	39	41	9 - Dorsolateral prefrontal cortex	66,60628263	29
						46 - Dorsolateral prefrontal cortex	24,84615413	
Fz-F2	Fz	F2	10	41	50	9 - Dorsolateral prefrontal cortex	68,93306313	29
						8 - Includes Frontal eye fields	28,89129929	
Fz-AFz	Fz	AFz	2	50	39	9 - Dorsolateral prefrontal cortex	61,76828691	40
						10 - Frontopolar area	20,26301996	
						8 - Includes Frontal eye fields	12,14994939	
						32 - Dorsal anterior cingulate cortex	5,083608014	

For peer review only

Fz-F1	Fz	F1	-9	41	50	9 - Dorsolateral prefrontal cortex	63,16113601	29
						8 - Includes Frontal eye fields	34,73195285	

Please note that this output was generated using FOLD-software (G. A. Zimeo Morais, J. B. Balardin, and J. R. Sato, “fNIRS Optodes' Location Decider (fOLD): A toolbox for probe arrangement guided by brain regions-of-interest,” Scientific reports, vol. 8, no. 1, p. 3341, 2018.).

For peer review only
